# Unveiling the Therapeutic Potential of the Second-Generation Incretin Analogs Semaglutide and Tirzepatide in Type 1 Diabetes and Latent Autoimmune Diabetes in Adults

**DOI:** 10.3390/jcm14041303

**Published:** 2025-02-15

**Authors:** Marco Infante, Francesca Silvestri, Nathalia Padilla, Francesca Pacifici, Donatella Pastore, Marcelo Maia Pinheiro, Massimiliano Caprio, Manfredi Tesauro, Andrea Fabbri, Giuseppe Novelli, Rodolfo Alejandro, Antonino De Lorenzo, Camillo Ricordi, David Della-Morte

**Affiliations:** 1 Section of Diabetes & Metabolic Disorders, UniCamillus, Saint Camillus International University of Health Sciences, Via di Sant’Alessandro 8, 00131 Rome, Italy; 2 Division of Cellular Transplantation, Diabetes Research Institute (DRI), Department of Surgery, University of Miami Miller School of Medicine, 1450 NW 10th Ave., Miami, FL 33136, USA; nxp467@med.miami.edu (N.P.); ralejand@med.miami.edu (R.A.); cricordi@med.miami.edu (C.R.); 3 Pediatric Endocrinology Outpatient Clinic, Via dell’Alpinismo 24, 00135 Rome, Italy; francesca.silvestri89@gmail.com; 4 Department of Human Sciences and Promotion of the Quality of Life, San Raffaele Open University, Via di Val Cannuta 247, 00166 Rome, Italy; francesca.pacifici@uniroma2.it (F.P.); donatella.pastore@uniroma5.it (D.P.); massimiliano.caprio@sanraffaele.it (M.C.); david.dellamorte@uniroma2.it (D.D.-M.); 5 Interdisciplinary Center for Advanced Studies on Lab-on-Chip and Organ on-Chip Applications (IC-LOC), University of Rome Tor Vergata, Via Montpellier 1, 00133 Rome, Italy; 6 UNIVAG, Centro Universitário de Várzea Grande, Av. Dom Orlando Chaves, 2655-Cristo Rei, Várzea Grande 78118-000, MT, Brazil; marcelo.pinheiro@univag.edu.br; 7 Department of Systems Medicine, University of Rome Tor Vergata, Via Montpellier 1, 00133 Rome, Italy; manfredi.tesauro@ptvonline.it (M.T.); andrea.fabbri@uniroma2.it (A.F.); 8 Genetics Section, Department of Biomedicine and Prevention, University of Rome Tor Vergata, Via Montpellier 1, 00133 Rome, Italy; novelli@med.uniroma2.it; 9 Department of Pharmacology, Reno School of Medicine, University of Nevada, 1664 N. Virginia Street, Reno, NV 89557, USA; 10 Section of Clinical Nutrition and Nutrigenomics, Department of Biomedicine and Prevention, University of Rome Tor Vergata, Via Montpellier 1, 00133 Rome, Italy; delorenzo@uniroma2.it; 11 Department of Neurology, Evelyn F. McKnight Brain Institute, University of Miami Miller School of Medicine, 1120 NW 14th Street, Miami, FL 33136, USA

**Keywords:** type 1 diabetes, T1D, LADA, autoimmune diabetes, overweight, obesity, GLP-1, GIP, C-peptide, beta-cell function, double diabetes, semaglutide, tirzepatide

## Abstract

Type 1 diabetes mellitus (T1D) is a chronic autoimmune disease caused by the immune-mediated destruction of insulin-producing pancreatic beta cells, resulting in the lifelong need for exogenous insulin. Over the last few years, overweight and obesity have recently emerged as growing health issues also afflicting patients with T1D. In this context, the term “double diabetes” has been coined to indicate patients with T1D who have a family history of type 2 diabetes mellitus (T2D) and/or patients with T1D who are affected by insulin resistance and/or overweight/obesity and/or metabolic syndrome. At the same time, the use of second-generation incretin analogs semaglutide and tirzepatide has substantially increased on a global scale over the last few years, given the remarkable clinical benefits of these drugs (in terms of glucose control and weight loss) in patients with T2D and/or overweight/obesity. Although the glucagon-like peptide-1 (GLP-1) receptor agonists and the novel dual GIP (glucose-dependent insulinotropic polypeptide)/GLP-1 receptor agonist tirzepatide are currently not approved for the treatment of T1D, a growing body of evidence over the last few years has shown that these medications may serve as valid add-on treatments to insulin with substantial efficacy in improving glucose control, promoting weight loss, preserving residual beta-cell function and providing other beneficial metabolic effects in patients with T1D, double diabetes and latent autoimmune diabetes in adults (LADA). This manuscript aims to comprehensively review the currently available literature (mostly consisting of real-world studies) regarding the safety and therapeutic use (for different purposes) of semaglutide and tirzepatide in patients with T1D (at different stages of the disease), double diabetes and LADA.

## 1. Introduction: Incretin Analogs, Autoimmune Diabetes, Overweight and Obesity

Incretin analogs are drugs that mimic the physiologic actions of gut-derived peptide hormones (also referred to as “incretin hormones” or “incretins”) secreted by enteroendocrine cells in response to food ingestion [1,2]. Thanks to their glucose-lowering and weight loss effects, incretin analogs were first approved for the treatment of type 2 diabetes mellitus (T2D) and subsequently for chronic weight management in people with obesity or overweight associated with at least one weight-related comorbidity [3]. Over the last two decades, these drugs have revolutionized the management of T2D and overweight/obesity [3]. However, over the last decade, it has become increasingly evident that these medications should not simply be regarded as glucose-lowering and weight loss drugs, as they have been proven to exert significant cardioprotective and nephroprotective actions in patients with T2D and/or overweight/obesity [3,4,5,6,7,8]. In particular, the pleiotropic effects of incretin analogs are probably mediated, at least in part, by their anti-inflammatory and antioxidant properties. In fact, evidence suggests that incretin hormones and incretin analogs can reduce oxidative stress [9], systemic inflammation [10], gut inflammation [11], adipose tissue inflammation [12] and allergen-induced lung and airway inflammation [13]. This review aims to comprehensively discuss the studies that have examined the therapeutic role of second-generation incretin analogs (semaglutide and tirzepatide) in patients with type 1 diabetes mellitus (T1D) and latent autoimmune diabetes in adults (LADA).

### 1.1. Type 1 Diabetes Mellitus (T1D)

Type 1 diabetes mellitus (T1D or T1DM) is a chronic autoimmune disease caused by the immune-mediated destruction of insulin-producing pancreatic beta cells, resulting in the progressive loss of endogenous insulin secretion, hyperglycemia, and a lifelong need for exogenous insulin [14]. T1D accounts for about 5–10% of all cases of diabetes mellitus [15]. Although the incidence of T1D peaks in puberty and early adulthood, disease onset can occur at any age [16]. Over the last few decades, there has been a gradual rise in the incidence of T1D, which amounts to an annual increase of approximately 3–4% [17,18], and it has mainly been attributed to changes in the environment [19]. Indeed, T1D is considered a complex multifactorial disease in which susceptibility genes and environmental factors interact and cause the triggering of autoimmune destruction of pancreatic beta cells [20].

The natural history of T1D is characterized by four sequential stages, as follows:

- **Stage 1**: Subjects exhibit beta-cell autoimmunity [as evidenced by the presence of at least two pancreatic islet autoantibodies: glutamic acid decarboxylase autoantibodies (GADA), tyrosine phosphatase-related islet antigen 2 autoantibodies (IA-2A), insulin autoantibodies (IAA), zinc transporter 8 autoantibodies (ZnT8A), and pancreatic islet cell antibodies (ICA)], but maintain normoglycemia and remain asymptomatic.
- **Stage 2**: Subjects maintain beta-cell autoimmunity and remain asymptomatic, but exhibit abnormal blood glucose values (dysglycemia), as evidenced by the presence of impaired fasting glucose, an abnormal oral glucose tolerance test, and/or a glycated hemoglobin (HbA1c) value ≥5.7% (≥39 mmol/mol). Therefore, stages 1 and 2 are part of the so-called “presymptomatic T1D”.
- **Stage 3**: Subjects experience the onset of symptomatic disease (clinical onset of T1D), with clinical manifestations of insulin deficiency and overt hyperglycemia, such as polyuria, polydipsia, weight loss, and/or fatigue, which can subsequently lead to the development of diabetic ketoacidosis (DKA).
- **Stage 4**: Stage 4 marks the postdiagnosis period of long-standing disease (long-standing T1D) [21].

About two-thirds of patients with new-onset T1D experience a transient spontaneous partial remission phase (a.k.a. “honeymoon phase”) shortly after the initiation of insulin therapy [22,23]. This phase is classically characterized by a substantial reduction in exogenous insulin requirements (or, in rare instances, by the lack of need for exogenous insulin) associated with near-normoglycemia [22], as a likely consequence of both metabolic and immune factors contributing to temporary beta-cell recovery (i.e., reduction in beta cell glucotoxicity and improvement in insulin sensitivity due to the optimization of glucose control following the initiation of insulin therapy; transient restoration of immune tolerance to beta-cell autoantigens) [23,24]. Moreover, it is important to note that patients with long-standing T1D can exhibit detectable levels of serum C-peptide (a surrogate marker of endogenous insulin secretion), together with the persistence of insulin-producing pancreatic islets, for decades after the disease onset [25,26,27]. This aspect has relevant clinical implications, since the persistence of detectable serum C-peptide levels has been associated with various clinical benefits throughout the course of T1D, including better glucose control, lower total daily insulin requirements, lower risk of hypoglycemia, decreased glycemic variability, reduced risk of chronic complications of diabetes (such as retinopathy and nephropathy), and reduced risk of DKA [28,29,30,31]. Therefore, interventions that aim to halt autoimmune beta-cell destruction and preserve endogenous insulin secretion are highly desirable in patients with T1D [32].

Moreover, it is important to underscore that T1D is characterized by significant interindividual heterogeneity regarding pathophysiological, genetic, immunopathological and metabolic features (e.g., severity of autoimmune responses against pancreatic beta cells, rate of beta-cell loss, proportion of residual insulin-containing pancreatic islets, and degree of C-peptide preservation) [33,34,35]. Hence, T1D patients can be classified into different subtypes based on the existence of distinct etiopathological processes (endotypes), autoimmune responses (immunotypes) and degrees of responsiveness to potential disease-modifying agents, such as immunotherapies (theratypes) [33,34,35]. This heterogeneity influences the clinical course and progression of T1D through its sequential stages [34].

Based on recent estimates, approximately 8.4 million individuals are affected by T1D on a global scale [36]. Although a definitive biological cure for this disease is still not available, a growing body of evidence over the last few years has shown that immunotherapeutic agents (including the FDA-approved humanized anti-CD3 monoclonal antibody teplizumab) and investigational beta-cell replacement strategies based on the use of encapsulated stem cell-derived beta cells can modify the natural history of T1D at different disease stages [32,37,38,39,40,41]. Even though insulin therapy (administered through multiple daily subcutaneous injections via insulin pens or through continuous subcutaneous infusion via insulin pumps) remains the mainstay of T1D management [42], it is associated with an increased risk of hypoglycemia and weight gain [43,44].

Over the last years, overweight and obesity have emerged as growing health issues also afflicting T1D patients, in contrast with the outdated belief that T1D is a disease of lean individuals [45]. In fact, international large-scale registries have estimated that the prevalence of overweight and obesity in T1D patients ranges from 15.3% to 36.8% [45,46] and is therefore comparable to that observed in the general population [47]. In this context, the term “double diabetes” has been coined to indicate T1D patients who have a family history of T2D, and/or T1D patients who are affected by insulin resistance (IR) and/or overweight/obesity and/or metabolic syndrome [48,49]. Indeed, metabolic syndrome is becoming increasingly common even in patients with T1D [50].

Despite the remarkable advances in artificial pancreas systems, continuous glucose monitoring (CGM) sensors and novel insulin analogs over the last years, achieving adequate glucose control remains challenging for many pediatric and adult patients with T1D [51] due to several factors, such as weight gain, hypoglycemia (including exercise-induced hypoglycemia), high glycemic variability, postprandial glucose excursions (early postprandial hyperglycemia and late postprandial hypoglycemia), possible presence of binge eating disorder and disordered eating behaviors, as well as other factors that can negatively affect physical activity routines and adherence to insulin therapy (e.g., fear of hypoglycemia, psychological factors, and poor acceptance of the disease) [45,52,53,54,55,56,57]. The aforementioned factors are major hurdles in improving glucose control in a significant proportion of T1D patients. The concomitant presence of overweight or obesity in T1D patients further complicates the achievement and maintenance of adequate glucose control. Indeed, management of overweight and obesity in patients with T1D (double diabetes) can be particularly challenging, as these subjects need to maintain a balance between adequate caloric intake and increased physical activity while limiting the risk of hypoglycemia, preventing increases in their daily insulin requirements, and avoiding further weight gain [45].

Given the growing obesity pandemic (also afflicting children and adolescents) [58,59,60] and the increasing incidence of T1D [17], the prevalence of double diabetes is likely to concomitantly increase [61]. This epidemiologic trend is particularly alarming, as insulin resistance and overweight/obesity make the achievement or maintenance of adequate glucose control in T1D patients more challenging and concomitantly increase the risk of cardiovascular diseases, hypertension and metabolic disorders, such as dyslipidemia [62,63,64,65,66,67]. Moreover, double diabetes is associated with an increased risk of both macrovascular and microvascular complications of diabetes [48,68,69].

In view of the above, there is an urgent need for non-insulin adjunct therapies that can effectively treat or prevent excess weight and reduce the risk of cardiometabolic diseases in patients with T1D.

### 1.2. Latent Autoimmune Diabetes in Adults (LADA)

Compared to T1D, latent autoimmune diabetes in adults (LADA) represents a distinct type of autoimmune diabetes characterized by an older age of onset and by a slower and less severe immune-mediated destruction and functional deterioration of pancreatic beta cells, resulting in a slower progression to insulin dependence [70]. Key diagnostic criteria for LADA include the following: (a) adult-onset diabetes (age greater than 30 years at the time of diagnosis); (b) presence of islet autoantibodies (with GADA being considered the predominant islet autoantibodies and the most sensitive laboratory marker of LADA); and (c) absence of insulin requirement for at least 6 months after the disease diagnosis [71]. However, defining categorical immunogenetic and phenotypic characteristics of LADA is difficult given that this condition is characterized by marked clinical heterogeneity and shares clinical and metabolic features with both T1D and T2D [70,71]. Indeed, LADA patients exhibit significant variability in the rate of beta-cell destruction, varying degrees of preservation of endogenous insulin secretion, heterogeneous patterns of islet autoimmunity, as well as varying degrees of insulin resistance, probably as a consequence of differences in genetic and immune factors [70,71]. Interestingly, a similar type of slowly progressive autoimmune diabetes has also been described in young-onset cases and is referred to as latent autoimmune diabetes in the young (LADY) [71,72].

LADA accounts for about 2–12% of all cases of adult-onset diabetes [71]. Given its adult-onset and heterogeneous clinical presentation, LADA is often misdiagnosed and treated as T2D; this can delay proper treatment, resulting in poor glucose control, more rapid progression to insulin dependence, and increased risk of chronic complications of diabetes [73]. Therefore, as in the case of T1D patients, therapeutic interventions able to preserve beta-cell function are highly desirable in LADA patients [73].

### 1.3. Novel Incretin Analogs: Semaglutide and Tirzepatide

Incretins [mainly glucose-dependent insulinotropic polypeptide (GIP, a.k.a. gastric inhibitory polypeptide) and glucagon-like peptide-1 (GLP-1)] are gut hormones released by enteroendocrine cells into the bloodstream in response to food intake [1,2]. GIP (secreted by enteroendocrine K cells, which are predominantly located in the duodenum) and GLP-1 (secreted by enteroendocrine L cells, which are located in the distal gut) are rapidly degraded by the ubiquitous enzyme dipeptidyl peptidase-4 (DPP-4) [1,2,74]. Both endogenous GIP and GLP-1 have very short half-lives, which are measured in minutes [75]. Once released into the bloodstream, GIP and GLP-1 potentiate glucose-stimulated insulin secretion (GSIS) by pancreatic beta cells, regulate glucagon secretion from pancreatic alpha cells, and exert anorectic actions by binding to their receptors, which are expressed in different regions of the central nervous system involved in the regulation of appetite [1,2,76,77,78]. GIP stimulates glucagon secretion (in a glucose-dependent manner) and inhibits gastric acid secretion, whereas GLP-1 inhibits glucagon secretion, slows gastric emptying, inhibits gastric acid secretion, and attenuates small bowel motility [1,2,77,79].

Therefore, the insulinotropic, glucoregulatory and anorectic actions of GIP and GLP-1 have supported the development of incretin-based therapies for the treatment of T2D and obesity [1]. In particular, among the incretin analogs, there are drugs acting as GLP-1 receptor agonists (GLP-1 RAs) or dual GIP/GLP-1 receptor agonists (dual GIP/GLP-1 RAs), which are biochemically modified for enhanced potency and prolonged action, as they are not susceptible to DPP-4-mediated inactivation [80,81]. These medications mimic the physiologic actions of endogenous GLP-1 and GIP, thus exerting antihyperglycemic effects, promoting satiety, reducing appetite and inducing weight loss [82].

Importantly, native GLP-1 and GLP-1 RAs have been shown to exert multiple beneficial actions in the cardiovascular system by reducing blood pressure in patients with hypertension, attenuating ischemic cardiac injury, mitigating inflammation in the heart and blood vessels and exerting anti-atherogenic effects [1,83]. GLP-1 RAs have been shown to significantly reduce the risk of major adverse cardiovascular events and to slow the progression of chronic kidney disease in clinical trials involving patients with T2D and/or overweight/obesity [5,6,7,84].

It is also interesting to note that animal studies have shown that GLP-1 and GLP-1 RAs can expand beta-cell mass by reducing beta-cell apoptosis and stimulating beta-cell proliferation and beta-cell neogenesis (differentiation of beta cells from ductal progenitor cells) [85,86,87,88]. Moreover, experimental studies conducted on well-differentiated beta cell lines (INS-1 cells) showed that GIP also promotes beta-cell proliferation and inhibits beta-cell apoptosis [89,90]. Hence, both GLP-1 RAs and dual GIP/GLP-1 RAs have the potential to promote beta-cell proliferation and survival.

The GLP-1 RA semaglutide and the dual GIP/GLP-1 RA tirzepatide are second-generation, long-acting incretin analogs approved for the treatment of T2D and for chronic weight management in patients with obesity or overweight associated with at least one weight-related comorbidity (such as hypertension, T2D and hypercholesterolemia) [3,91]. Of note, tirzepatide is a “first-in-class” and the only commercially available GIP–GLP-1 co-agonist (a.k.a. “twincretin”) at the present time [92], and it received marketing authorization in the US after semaglutide [82]. Furthermore, in 2024, the U.S. Food and Drug Administration (FDA) approved the use of semaglutide to reduce the risk of cardiovascular death, heart attack and stroke in adults with cardiovascular disease and either overweight or obesity [93]. In the same year, the FDA also approved the use of tirzepatide for the treatment of moderate to severe obstructive sleep apnea (OSA) in adults with obesity [94], following the results of two randomized controlled trials demonstrating that this drug provided significant reductions in the apnea–hypopnea index in this population [95].

Retrospective studies have already suggested that the use of GLP-1 RAs (including semaglutide) as non-insulin adjunct therapies may be effective in improving glucose control and reducing insulin requirements and excess weight in T1D patients [96,97]. In two double-blind, randomized, placebo-controlled phase 3 trials (ADJUNCT ONE and ADJUNCT TWO), the GLP-1 RA liraglutide —in addition to insulin therapy— reduced HbA1c levels, insulin requirements and body weight in patients with long-standing T1D; however, reductions in HbA1c levels were modest and occurred at the cost of increased rates of hypoglycemia and hyperglycemia with ketosis [98,99]. Moreover, a multicenter, double-blind, parallel-group trial found that liraglutide treatment leads to significant preservation of residual beta-cell function and reduction in daily insulin requirements during the first year after diagnosis in patients with new-onset T1D [100]. Similarly, a post-hoc analysis performed using data from three randomized phase 3 trials (AWARD-2,-4,-5) documented that the GLP-1 RA dulaglutide is effective in lowering HbA1c values over a 12-month follow-up period in patients with LADA [101]. Furthermore, GLP-1 RAs have been shown to exert beneficial effects in terms of metabolic control in patients with LADA, unless circulating C-peptide levels are markedly reduced [71].

Although GLP-1 RAs and the dual GIP/GLP-1 RA tirzepatide are currently not approved for the treatment of T1D, growing evidence over the last few years has shown that these medications may serve as valid add-on treatments to insulin with substantial efficacy in improving glucose control, promoting weight loss and determining other beneficial effects (including preservation of residual beta-cell function) in patients with autoimmune diabetes. This evidence primarily comes from real-world data on the use of semaglutide and tirzepatide in T1D patients with concomitant overweight/obesity, although a randomized crossover trial investigating the efficacy of semaglutide in adults with T1D using an automated insulin delivery system has recently been published (discussed later in the manuscript). Therefore, this manuscript aims to comprehensively review the currently available literature regarding the use of the new incretin analogs semaglutide and tirzepatide in patients with T1D (at different stages of the disease) and LADA, examining the safety and efficacy of these medications administered for different purposes in real-world settings.

## 2. Materials and Methods

We conducted a narrative review of the literature regarding the use (for different purposes) of the novel incretin analogs semaglutide and tirzepatide in patients with T1D (at different stages of the disease) and LADA within a real-world context. The search strategy consisted of capturing all publications pertaining to the use of semaglutide and tirzepatide in patients with T1D and LADA on the MEDLINE/PubMed and Scopus scientific databases. Only one article (case report) published in a journal not indexed in PubMed and Scopus [102] was identified by reviewing the reference list of another article published in a journal indexed in PubMed [103]. The search was conducted using the following keywords: “type 1 diabetes” or “type 1 diabetes mellitus” or “T1D” or “T1DM” or “Latent autoimmune diabetes in adults” or “Latent autoimmune diabetes of adults” or “LADA” or “Latent autoimmune diabetes in the young” or “LADY” or “autoimmune diabetes” or “autoimmune diabetes mellitus” AND “semaglutide” or “tirzepatide”. Only original articles and case reports written in English and published between 5 December 2017 (date of the first global semaglutide approval in the US as an adjunct to diet and exercise to improve glucose control in adults with T2D) [104] and 23 January 2025 were considered for inclusion in the present review. We describe the retrieved articles related to the topic of the present review starting from the highest level of evidence (in this case: randomized controlled trials) to the lowest level of evidence (case reports).

## 3. Clinical Studies on the Use of Semaglutide in T1D

### 3.1. Randomized Controlled Trials (RCTs)

At the time of the present writing, only one randomized controlled trial investigating the use of semaglutide in T1D patients has been published [105]. This study currently represents the highest level of evidence regarding the therapeutic role of semaglutide in patients with T1D. Of note, Pasqua et al. [105] conducted a randomized, double-blind crossover trial at the Research Institute of the McGill University Health Centre in Montreal (Quebec, Canada) to evaluate whether once-weekly subcutaneous semaglutide, compared to placebo, improves glucose control and other non-glycemic outcomes in adults with T1D while using an automated insulin delivery (AID) system. AID systems (a.k.a. closed-loop systems or artificial pancreas systems) currently represent the most advanced form of insulin therapy for T1D and consist of a control algorithm that automatically and dynamically drives insulin delivery from a subcutaneous infusion pump based on real-time and predicted interstitial glucose levels measured through a CGM sensor [106].

Among the inclusion criteria of the study published by Pasqua et al. [105], there was the diagnosis of T1D for 1 year or longer. Each arm was 15 weeks (with 2 weeks of washout) and the total study duration was 32 weeks. After the initial visit, at each intervention, participants were titrated up to 1 mg or the maximum tolerated dose of semaglutide or placebo over a 11-week dose titration period. Intervention doses were de-escalated in case of participants’ intolerance of side effects. Participants remained on their usual pump therapy during the entire dose titration period. The titration period was followed by the use of a research-based AID system for 4 weeks. The AID system included a Dexcom G6 CGM sensor, an Ypsomed insulin pump, and a Pixel 2 smartphone with an application running the McGill insulin dosing algorithm, as previously described [107]. At the end of the 4-week AID use, anthropometric measurements and laboratory testing were performed [105]. Participants returned to their usual insulin for a 2-week washout period. After the washout period, the second study drug was started, with identical procedures to the first intervention. The primary endpoint was the percentage of time spent in the target glucose range (3.9–10.0 mmol/L; 70–180 mg/dL) between semaglutide (at the maximum tolerated dose) and placebo, during the 4 weeks of AID use. Secondary endpoints included the mean glucose level, the standard deviation (SD), and the coefficient of variation (CV) of glucose levels (which are markers of glycemic variability) [108], time spent in hyperglycemia (above 10.0 mmol/L and above 13.9 mmol/L; above 180 mg/dL and above 250 mg/dL), and time spent in hypoglycemia (below 3.0 mmol/L and below 3.9 mmol/L; below 54 mg/dL and below 70 mg/dL) [105].

Of the 113 adults with T1D who were initially screened, 28 participants (female participants: *n* = 17; 61%) were recruited and randomized. The mean age of the study participants was 45 years, whereas the mean diabetes duration was 28 years. At baseline, 22 participants (79%) were using commercial AID systems, while 7 participants (25%) were affected by overweight, and 18 participants (64%) were affected by obesity. Moreover, less than one-third of participants (*n* = 8; 29%) met the HbA1c target (<7%) at baseline. Of the 28 participants who were randomized, 24 completed the trial.

The primary endpoint was met. Indeed, compared to the placebo, semaglutide significantly increased the time in range (TIR) without increasing the time below range (TBR). For the last 28 days of each intervention, during the use of the study’s AID system, the mean TIR of 3.9–10.0 mmol/L was 74.2% for semaglutide vs. 69.4% for placebo, a significant paired difference of 4.8 percentage points. Moreover, during the last 28 days of AID use, there was no difference in mean values of TBR < 3.9 mmol/L (TBR < 70 mg/dL) between the interventions, indicating that the increase in TIR was entirely due to a decrease in time spent in hyperglycemia [a.k.a. TAR, time above range]. Indeed, there was a significant difference between the interventions in mean values of TAR > 10.0 mmol/L (TAR > 180 mg/dL) (semaglutide 24.1% vs. placebo 29.1%; paired difference of 5.0%) and in median values of TAR > 13.9 mmol/L (TAR > 250 mg/dL) (semaglutide 5.4% vs. placebo 8.4%; paired difference of 1.7%).

Mean glucose levels and SD of glucose levels were also significantly reduced with semaglutide use (8.4 mmol/L and 3.0 mmol/L, respectively) compared to placebo (8.8 mmol/L and 3.2 mmol/L, respectively), although the CV of glucose levels was not. In addition, there was a significant reduction with semaglutide, compared to placebo, in the median HbA1c and fructosamine values (placebo-adjusted change: HbA1c, −0.5% [interquartile range (IQR): −0.7, −0.2]); fructosamine, −15 μmol/L [IQR: −39, 2]). The proportion of participants who achieved an HbA1c value <7% was also significantly higher with semaglutide use compared to placebo [semaglutide, *n* = 17 (71%); placebo, *n* = 10 (42%)].

The mean high-density lipoprotein (HDL) cholesterol value was also significantly lower with semaglutide than placebo (1.29 mmol/L vs. 1.47 mmol/L, respectively; placebo-adjusted change: −0.17 mmol/L), whereas other blood lipid levels [total cholesterol, triglycerides, low-density lipoprotein (LDL) cholesterol, non-HDL cholesterol] did not significantly change. However, the reduction in HDL cholesterol observed with semaglutide use was not considered clinically meaningful by the researchers.

The mean body weight and the body mass index (BMI) were significantly reduced by 5.3 kg and 1.9 kg/m^2^ with semaglutide compared to placebo. The mean percent body weight reduction was −6.7% with semaglutide and −2.1% with placebo, constituting a significant −5.1% relative change from baseline. The mean waist circumference and hip circumference values were both significantly reduced with semaglutide compared to placebo (placebo-adjusted changes of −5.2 cm and −4.5 cm, respectively). Of note, participants who experienced the greatest weight reduction tended to exhibit the highest glycemic benefits, as supported by the Pearson correlation documenting that the reduction in body weight was significantly correlated with the increase in TIR and with the reduction in HbA1c values.

The median total daily dose (TDD) of insulin was significantly reduced by 11.3 units with semaglutide compared to placebo. This reduction was mediated by significant reductions in median values of both basal and bolus insulin doses. Furthermore, mean weight-based daily insulin requirements (which serve as a marker of insulin sensitivity) were significantly reduced with semaglutide (0.64 U/kg/day) compared to placebo (0.77 U/kg/day) [paired difference: −0.13]. The median value of total daily carbohydrate intake (as entered into the participants’ bolus calculator) was also significantly reduced by 36 g with semaglutide use compared to placebo. Interestingly, 6 out of the 24 participants who completed the trial (25%) had detectable random plasma C-peptide levels at baseline (defined as random plasma C-peptide levels greater than 0.003 nmol/L). Placebo-adjusted changes in mean TIR 3.9–10.0 mmol/L (TIR 70–180 mg/dL), body weight and HbA1c were 6.2 percentage points, −6.3 kg and −0.7%, respectively, in participants with detectable C-peptide levels, compared to 4.4 percentage points, −6.4 kg and −0.4%, respectively, in participants with undetectable C-peptide levels.

Moreover, in order to evaluate the generalizability of the study findings, the researchers performed a post-hoc analysis in 12 participants who were on the commercial Control-IQ technology during the last 14 days of the titration period. Placebo-adjusted outcomes of insulin use, TIR, TBR and carbohydrate intake were all comparable to the findings observed with the research-based AID system.

The most common adverse events reported during this trial were gastrointestinal in nature and mostly mild. Severe hypoglycemic events or DKA were not reported during semaglutide use. Although DKA did not occur, two participants in the study experienced overt episodes of euglycemic ketosis during semaglutide use. However, these episodes of euglycemic ketosis did not lead to acidosis (euglycemic ketosis without acidosis) and were resolved with proper changes in carbohydrate intake and insulin therapy. One of the two euglycemic ketosis cases was thought to be related to semaglutide use, while the other case was suspected to be unrelated to semaglutide use (as it occurred during concomitant foodborne illness and insulin pump malfunction). In two study participants, progression of clinically stable or regressed retinopathy was incidentally found: one episode of proliferative retinopathy during placebo use, and one non-proliferative sequela during semaglutide therapy. Yet, such events were not considered sight-threatening, as per ophthalmology consultation. There was only one serious adverse event during semaglutide use (a recurrent tibial fracture after a prior surgery), although this was deemed to be unrelated to the study drug.

In summary, the aforementioned study represents the first randomized controlled trial demonstrating the efficacy of semaglutide as an adjunct to AID in adults with T1D. Indeed, this trial showed that once-weekly subcutaneous semaglutide (at the maximum tolerated dose) improves glucose control (as assessed by TIR and HbA1c) —without increasing the time spent in hypoglycemia— and reduces insulin requirements as well as carbohydrate intake, body weight, BMI, and waist and hip circumferences in adults with T1D using an AID system.

The correlation between greater reductions in body weight and greater glycemic benefits observed in the study may be explained by the fact that weight loss can improve insulin sensitivity [109], which, in turn, leads to a reduction of insulin requirements and an improvement of glucose control in T1D. Another interesting finding of the aforementioned trial [105] is the evidence of greater HbA1c reductions in patients with detectable plasma C-peptide levels compared to those with undetectable plasma C-peptide levels. This finding suggests that combining semaglutide with agents that preserve residual beta-cell function may maximize the glycemic benefits of this novel incretin analog. Besides the evident glycemic and anthropometric benefits observed with semaglutide therapy in this trial, there were minimal changes in blood pressure and in non-glycemic laboratory outcomes. However, this may be due to the lack of blood pressure and non-glycemic laboratory abnormalities at baseline in the study participants.

This study has unquestionable strengths, such as the double-blind, randomized and controlled design. Nevertheless, it also has some limitations, including the small sample size and the short study duration [105]. Therefore, further RCTs are required to better assess the long-term efficacy and safety profile of semaglutide in patients with T1D.

### 3.2. Prospective Cohort Studies

Navodnik et al. [110] conducted the ENDIS study (Endothelium dysfunction assessment study), a 12-week intervention, prospective, randomized, single-center, controlled clinical study that enrolled 91 adult patients with long-standing T1D on insulin therapy [either with multiple daily injection (MDI) insulin therapy or continuous subcutaneous insulin infusion (CSII)], all using CGM systems. The study aimed to assess and compare the metabolic and endothelial-function-related effects of empagliflozin —a sodium-glucose cotransporter-2 (SGLT2) inhibitor— and subcutaneous semaglutide as add-on treatments to insulin. Of the 91 participants who were initially enrolled in the study, 89 participants completed the study. Two patients withdrew from the study: one due to the side effects related to semaglutide (nausea and persistent vomiting) and one in the control group due to personal reasons. Participants were randomized to receive empagliflozin (10 mg/day) as add-on treatment to insulin, or once-weekly subcutaneous semaglutide as add-on treatment to insulin [semaglutide dose titration from 0.25 to 1 mg according to the recommendations from the SmPC (Summary of Product Characteristics) or to the maximum tolerated dose], or insulin therapy alone (control group).

Demographic and baseline characteristics (age, sex, diabetes duration, smoking status, BMI, body weight, waist circumference, and estimated glomerular filtration rate based on cystatin C) were similar between groups, except for baseline mean HbA1c values, which were significantly (but slightly) higher in the two treatment groups: empagliflozin group, 7.88%; semaglutide group, 7.42%; control group, 7.04%.

After 12 weeks, mean body weight values (empagliflozin group: −2.49 kg; semaglutide group: −4.3 kg; control group: −0.10 kg) and mean waist circumference values (empagliflozin group: −4.0 cm; semaglutide group: −4.4 cm; control group: −0.97 cm) decreased from baseline in all groups, and this reduction was statistically significant in both treatment groups compared to baseline values and compared to 12-week values in the control group. Moreover, the reduction in mean body weight values was significantly greater in the semaglutide group compared to the empagliflozin group.

The reduction in mean HbA1c values from baseline to week 12 was significant in the semaglutide group (−0.29%), but non-significant in the empagliflozin group (−0.24%). Pairwise comparisons revealed a significant reduction in HbA1c in both the treatment groups compared to the control group. However, there was no significant difference regarding changes in TIR 3.9–10.0 mmol/L (70–180 mg/dL) between the empagliflozin group and the semaglutide group. Participants in both treatment groups experienced a significant reduction in mean TDD of insulin compared to controls (−1.1 IU/day) [semaglutide group, −8.5 IU/day; empagliflozin group, −5.1 IU/day], but there was no significant difference in mean TDD of insulin between the two treatment groups.

There was also a statistically significant reduction in mean LDL cholesterol values in the semaglutide group from baseline (−0.30 mmol/L) and compared to controls (−0.15 mmol/L) and the empagliflozin group (0.03 mmol/L).

There was a significant improvement in FMD (brachial artery flow-mediated dilation) in both intervention groups after 12 weeks, as compared to the baseline, with no significant changes observed between those two groups. In the empagliflozin group, the mean FMD increased from 5.39% to 10.6% (2.0-fold), while in the semaglutide group the mean FMD increased from 5.81% to 11.1% (1.9-fold). Mean peripheral resistance (PR) decreased in both intervention groups, although this reduction was statistically significant only in the semaglutide group (−0.07 mmHg/L/min).

With regard to the safety and tolerability of the therapeutic interventions, the authors reported only mild side effects and only one case of semaglutide withdrawal due to persistent vomiting, with no cases of severe hypoglycemia or DKA being documented [110]. These findings suggest that both empagliflozin and semaglutide (as add-on treatments to insulin) positively impact endothelial function in patients with T1D.

### 3.3. Retrospective Cohort Studies

Mohandas et al. [111] conducted a real-world retrospective chart review study on a cohort of 54 adult patients with long-standing T1D who had been on long-acting GLP-1 RAs (including semaglutide, exenatide extended-release, dulaglutide, albiglutide) for at least 6 months. The retrospective chart review included data from 2 years before GLP-1 RA therapy initiation and data from 6 or more months after GLP-1 RA therapy initiation. At baseline, patients had a mean age of 41.5 years. The majority of patients (*n* = 34; 63.0%) were using once-weekly subcutaneous semaglutide as GLP-1 RA, whereas the remaining patients were using dulaglutide (*n* = 19; 35.2%), exenatide extended-release (*n* = 2; 3.7%) and albiglutide (*n* = 2; 3.7%). However, specific data regarding the medication dose and the changes in body weight and markers of glucose homeostasis in semaglutide-treated group were not available. Baseline and post-GLP-1 RA initiation values were calculated for each parameter by averaging values over a 2-year period before starting the GLP-1 RAs and values collected starting from 6 months after the initiation of GLP-1 RA therapy (and ending at the time of chart review). The mean duration of GLP-1 RA therapy was 23.85 months. The authors found that mean HbA1c values decreased significantly from a baseline of 7.76% to 7.05% (mean difference = −0.71%) and that mean body weight decreased significantly from a baseline of 86.66 kg to 83.50 kg (mean difference = −3.16 kg). Mean TIR increased significantly from a baseline of 54.59% to 66.74% (mean difference = +12.15%), while mean TAR decreased significantly from a baseline of 42.20% to 30.23% (mean difference = −11.97%). The mean SD of glucose decreased significantly from a baseline of 56.09 mg/dL to 47.64 mg/dL (mean difference = −8.45 mg/dL). Mean 14-day CGM glucose values decreased significantly from a baseline of 182 mg/dL to 163 mg/dL (mean difference = −19 mg/dL). Mean TBR decreased, although not significantly, from a baseline of 2.32% to 1.78% (mean difference = −0.54%). There was also a non-significant decrease in mean daily insulin requirements, from 0.553 units/kg/day to 0.547 units/kg/day (mean difference = −0.0061 units/kg/day). There was no statistically significant difference between baseline and post-GLP-1 RA initiation values of serum creatinine, total cholesterol, LDL cholesterol, systolic blood pressure, or diastolic blood pressure.

Only 15 patients (27.8%) discontinued GLP-1 RA therapy over a 2-year period. The most common reasons for GLP-1 RA discontinuation were gastrointestinal side effects, such as nausea and vomiting (40.0%), minimal or negative impact of GLP-1 RAs on glucose control (20.0%), and lack of insurance coverage (6.7%). The remaining 33.3% of patients discontinued the GLP-1 RAs for unknown reasons. There was no difference in the occurrence of hypoglycemia or DKA after the initiation of GLP-1 RA therapy, even considering that many participants (*n* = 23 out of 36 participants for which C-peptide values were available; 63.9%) had C-peptide values lower than 0.2 nmol/L. Therefore, this retrospective study suggested that long-acting GLP-1 RAs used for at least 6 months and up to 2 years can provide significant benefits (such as weight loss and improvement in glucose control) in patients with long-standing T1D, without being associated with an increased incidence of hypoglycemia or DKA [111].

Dandona et al. [112] published a retrospective study including 10 patients (aged 21 to 39 years) who started once-weekly subcutaneous semaglutide treatment within 3 months after the diagnosis of T1D. At the time of T1D diagnosis, the mean HbA1c level was 11.7%, whereas the mean fasting C-peptide level was 0.65 ng/mL. The entire cohort of patients followed dietary carbohydrate restriction and was treated with standard prandial and basal insulin. The weekly semaglutide dose was gradually titrated up to a maximum of 0.5 mg to avoid hypoglycemia and to monitor other side effects. Semaglutide treatment led to the withdrawal of prandial insulin in all patients (within 3 months) and to the withdrawal of basal insulin in the majority of patients (in 7 out of 10 patients; within 6 months). Remarkably, semaglutide treatment was associated with better glucose control and increased fasting C-peptide values during the 12-month observation period. The mean HbA1c fell to 5.9% and 5.7% at 6 months and at 12 months, respectively. At 12 months, the mean fasting C-peptide level increased to 1.05 ng/mL (+0.4 ng/mL vs. baseline), while the mean TIR from CGM was 89% [112,113]. Based on the substantial reduction in total daily insulin dose, near-normoglycemia (as documented by the achieved HbA1c values and CGM metrics) and increased fasting C-peptide levels observed at the end of the observation period, it is plausible that semaglutide treatment may have contributed to prolonging the honeymoon phase of T1D. In addition, the authors did not observe hypoglycemic episodes, DKA or other serious side effects after semaglutide dose stabilization [112]. However, it is important to highlight that the aforementioned study has limitations that prevent an accurate clinical interpretation, such as the retrospective design, the fact that data were uncontrolled, and the lack of details about dietary restriction and weight change in the analyzed cohort [114].

Grassi et al. [115] retrospectively extracted clinical and 30-day CGM data before and after the initiation of low-dose once-weekly subcutaneous semaglutide (0.5 mg/week) in a cohort of 11 adult patients with long-standing T1D and excess body weight (overweight or obesity) who were on PLGM (predictive low-glucose management) with sensor-augmented insulin pump therapy and adhered to structured lifestyle modifications and education for weight management. In particular, clinical and 30-day CGM data were extracted before the initiation of semaglutide therapy (T0) and at 3 (T3) and 6 months (T6) after the initiation of semaglutide therapy. At 6 months after the initiation of semaglutide therapy, there was a statistically significant body weight reduction amounting to 10.6% (−8.8 kg). Moreover, the proportion of patients with a BMI value greater than 30 kg/m^2^ at 6 months significantly decreased from 6/11 to 0/11, with two patients reaching a BMI value less than 25 kg/m^2^. There was also a concomitant significant reduction in mean carbohydrate intake from 137 to 94 g/day from baseline to T3, with a slight significant increase in mean carbohydrate intake to 109 g/day at T6. The mean number of daily meals significantly decreased from 4.5 at T0 to 3.7 at T3, without significant changes observed at T6. Mean TDD of insulin decreased significantly from T0 to T3 (from 45.6 U/day to 38.7 U/day) and remained stable at T6 (38.5 U/day), with significant reductions observed only in the prandial insulin dose (and not in the basal insulin dose). However, CGM metrics (TIR, TBR and TAR) remained statistically unchanged throughout the 6-month observation period. With regard to side effects, 4 out of 11 subjects experienced transitory nausea when initiating or up-titrating the semaglutide dose, with none of the patients experiencing vomiting or any other semaglutide-related side effect [115].

Garg et al. [116] conducted a 12-month retrospective case-control study on adult patients with long-standing T1D and comorbid overweight or obesity who were prescribed once-weekly subcutaneous semaglutide for at least 3 months. The study cohort included 50 patients on semaglutide who were compared with 50 computer-matched patients (for age, gender, weight, BMI, ethnicity and diabetes duration) who were not on semaglutide or any other GLP-1 RA or weight loss medication. All patients included in this study used a CGM device (with or without an insulin pump), thus allowing the researchers to evaluate the CGM metrics in both the semaglutide and control groups.

The semaglutide dose was gradually titrated during the first 3 months to induce tolerability. The mean weekly dose of semaglutide was 0.63 mg at 3 months, which increased to 0.78 mg, 0.86 mg, and 0.92 mg at 6, 9, and 12 months, respectively. Compared to the controls, semaglutide-treated patients experienced significantly greater reductions in body weight and BMI values, as well as significantly greater percent body weight loss. Notably, the group comparison at 12 months documented that semaglutide-treated patients experienced a significant mean body weight loss of 15.9 lbs. (−7.6% of the baseline body weight and −7.9% of the baseline BMI), whereas controls showed a mean body weight gain of 2.1 lbs. (+1.1% of the baseline body weight). There was a significantly higher overall decline in mean HbA1c values in the semaglutide group compared to the control group throughout the study period (−0.60% vs. −0.17%). However, there was no group difference in terms of overall changes in insulin doses. With regard to CGM metrics, the semaglutide group, as compared to the control group, showed significantly greater overall increases in mean TIR values (+6.2% vs. +0.7%), as well as significantly greater overall reductions in measures of glycemic variability [SD of glucose: −5.9 mg/dL vs. −0.8 mg/dL; CV of glucose: −1.4% vs. −0.8%], although no significant group difference was observed for overall changes in mean TAR and TBR values. None of the study participants reported any episode of severe hypoglycemia or DKA [116].

Almohareb et al. [117] conducted a multicenter retrospective study that aimed to assess the safety and efficacy of GLP-1 RAs in T1D patients (older than 16 years) based on real-world data. The study included 144 T1D patients who started GLP-1 RA therapy as an add-on treatment to insulin. The mean patient age was 33.0 years, with a mean diabetes duration of 16.5 years. At baseline, the mean patient body weight was 89.9 kg, the mean BMI was 34.0 kg/m^2^, the mean HbA1c was 8.9%, the mean fasting blood glucose was 214.5 mg/dL, and the mean TDD of insulin was 81.2 units/day (corresponding to 0.9 units/kg/day). The majority of patients (*n* = 133; 92.4%) were using MDI insulin therapy, whereas only 11 patients (7.6%) were on insulin pump therapy. Moreover, there was a limited number of patients using CGM systems (*n* = 27; 18.8%). Patients were followed until GLP-1 RA therapy was discontinued, the GLP-1 RA was replaced with another medication of the same class, or until 18 months after the initiation of GLP-1 RA therapy.

Among the study cohort, the majority of patients (*n* = 92; 63.9%) were using semaglutide (once-weekly subcutaneous semaglutide). Of the remaining 52 patients, 49 were using liraglutide and 3 were using dulaglutide. About one-fourth of semaglutide-treated patients decided to discontinue the medication (*n* = 21; 22.8%) before the end of the 18-month follow-up period. The mean semaglutide treatment duration was 7.9 months.

Among patients who underwent a follow-up visit within 4–6 months from GLP-1 RA therapy initiation (*n* = 74 patients), the mean HbA1c value significantly declined from 9.0% (at baseline) to 8.4% (mean ± SD [standard deviation] reduction: −0.5 ± 1.0%), and this was accompanied by significant mean reductions in the values of body weight (−3.6 kg), BMI (−1.4 kg/m^2^) and daily basal insulin dose (−2.4 units/day). Among patients who underwent a follow-up visit within 12–18 months (*n* = 38 patients) from GLP-1 RA therapy initiation, there was a significant mean reduction in the HbA1c value from baseline of −0.5%, and this was accompanied by significant mean reductions in the values of body weight (−5.2 kg), BMI (−1.9 kg/m^2^) and daily basal insulin dose (−4.1 units). Unfortunately, specific data on changes in HbA1c, body weight, BMI and TDD of insulin among the semaglutide-treated patients were not available. Yet, the authors demonstrated that the use of GLP-1 RAs (mostly semaglutide or liraglutide) as an add-on treatment to insulin resulted in a statistically significant reduction in HbA1c, body weight, BMI and basal insulin dose in T1D patients during an 18-month follow-up period. With regard to safety, only three patients (3.2%) in the semaglutide group discontinued the drug due to gastrointestinal side effects (nausea, vomiting or diarrhea), which represented the most frequently cited reasons for GLP-1 RA discontinuation in this subset of patients. Overall, GLP-1 RA therapy did not result in a higher occurrence of severe hypoglycemia or DKA [117]. The main limitations of this study, besides its retrospective design, are represented by the lack of a control group and the limited number of patients using CGM systems, which prevented an accurate analysis of the impact of GLP-1 RA therapy on CGM metrics [117].

Orrange et al. [118] recently conducted a retrospective chart review of 23 T1D patients (86% of whom were affected by overweight or obesity) in order to evaluate the impact of once-weekly subcutaneous semaglutide on glucose control and weight loss. The study cohort was predominantly composed of female participants (83%) and had a mean age and a mean diabetes duration of 45 years and 25 years, respectively. Study participants were on semaglutide therapy for at least 6 months and received primarily telemedicine-based care during and after the Coronavirus disease 2019 (COVID-19) pandemic over a follow-up period of 7 to 50 months. Semaglutide therapy led to a significant weight loss, with patients losing an average of 5% of their baseline body weight (approximately 3.75 kg) during a 22-month follow-up period. At baseline, 43% of participants were classified as affected by obesity (BMI ≥ 30 kg/m^2^). At the end of the follow-up period (after 9+ months), two participants had transitioned from obesity to overweight, while one participant had transitioned from overweight to normal weight. The majority of participants achieved weight loss with lower weekly semaglutide doses: 6 participants on 0.25 mg, 5 participants on 0.5 mg, 8 participants on 1.0 mg, and only 4 participants on 2.0 mg.

However, semaglutide did not lead to significant improvements in glucose control, as evidenced by the lack of statistically significant changes in the glucose management indicator (GMI) and TIR. TDD of insulin remained relatively unchanged, decreasing slightly from a mean value of 47.7 units/day (0.56 units/kg/day) at baseline to a mean value of 46.9 units/day (0.61 units/kg/day) at the end of the observation period, with no significant changes being observed during the follow-up period. These results suggest that the primary therapeutic benefit of semaglutide in patients with long-standing T1D affected by overweight/obesity is a relatively modest weight loss rather than significant improvements in glucose control and reductions in TDD of insulin. Nevertheless, the small sample size and the observational design of the study prevent definitive conclusions [118].

Cohen et al. [119] performed an ethics committee (Alfred Hospital, Melbourne, Australia) retrospective audit of their tertiary referral clinic to evaluate real-world outcomes in adult patients with T1D who were using adjunctive therapy with GLP-1 RA for two or more visits. Electronic medical records (from the Baker Heart and Diabetes Institute, Melbourne, Australia) were audited from 2012 onwards, with follow-up ending before the national GLP-1 RA shortage (February 2022). The authors used the estimated glucose disposal rate (eGDR) as a biomarker for insulin sensitivity in T1D (eGDR was expressed as the natural log: elnGDR = 3.091 − 0.141 × HbA1c) [120]. Among 1480 adults with T1D, 30 subjects (2%) were prescribed semaglutide and had one or more follow-up visits while they were still using the medication [119]. However, information regarding semaglutide formulations and dosages was not available. The mean follow-up duration was 255 days. The mean body weight decreased significantly by 8.2 kg (8.4% body weight reduction), whereas the mean HbA1c decreased significantly by 0.5% (to 8.0%). Five subjects achieved an HbA1c value < 7.0% (<53 mmol/mol). Moreover, there was a significant increase in mean eGDR by 0.05 (to 2.09). Blood pressure values, serum lipid profile and CGM metrics did not change significantly. There were no reported cases of DKA. Thus, real-world use of semaglutide was associated with statistically and clinically significant reductions in body weight and HbA1c, along with a significant improvement in insulin sensitivity (as assessed by eGDR) [119].

### 3.4. Case Series and Case Reports

Sofrà and Beer [102] first reported the case of a 32-year-old woman with overweight and a 20-year history of T1D, who was treated with a patch insulin pump and was prescribed semaglutide as non-insulin adjunct therapy to improve glucose control, reduce glycemic variability and counteract weight gain. At the time of semaglutide prescription, the patient had poor glucose control (HbA1c: 8.7%). The patient also suffered from non-proliferative diabetic retinopathy and maculopathy. Once-weekly subcutaneous semaglutide was prescribed at a starting dose of 0.25 mg/week for the first 4 weeks. The weekly semaglutide dose was then increased to 0.5 mg. At 3 months from the initiation of semaglutide therapy, flash glucose monitoring (FGM) already showed a substantial decrease in the average glucose level [8.3 mmol/L (149 mg/dL) vs. 10.8 mmol/L (194 mg/dL), −2.5 mmol/L (−45 mg/dL)], an increase in TIR 3.9–8.9 mmol/L (70–160 mg/dL) (63% vs. 38%), and a reduction in CV of glucose (which came closer to the desired therapeutic goal of ≤36%), even though these changes were accompanied by an increase in the percentage of time spent in hypoglycemia (6% vs. 2%). At 6 months from the initiation of semaglutide therapy, the patient experienced a 1.9% reduction in HbA1c (6.8%), a reduction in fasting plasma glucose [7.2 mmol/L (130 mg/dL) vs. 13.0 mmol/L (234 mg/dL), −5.8 mmol/L (−104 mg/dL)], a reduction in TDD of insulin (−5.2 U/day) and a body weight loss of 5 kg (corresponding to 8% body weight reduction), the latter being accompanied by a normalization of the BMI (24.8 kg/m^2^ vs. 26.8 kg/m^2^ at baseline). Moderate gastrointestinal side effects (mostly nausea) were reported by the patient, particularly during the semaglutide titration phase, although such side effects did not require any treatment [102].

Raven et al. [103] described the case of a 36-year-old woman with a 27-year history of T1D and undetectable fasting circulating C-peptide levels who needed better weight management due to overweight (BMI: 29.3 kg/m^2^). The patient was on MDI insulin therapy (with prandial insulin lispro and basal insulin glargine) plus metformin (1000 mg twice daily) and an oral contraceptive pill for polycystic ovary syndrome (PCOS). She also had stable and inactive proliferative diabetic retinopathy and was closely monitored through a FGM system. Subcutaneous semaglutide was initiated at a starting weekly dose of 0.25 mg, which was then up-titrated to 0.5 mg/week. The latter dose led to reduced appetite without significant nausea or gastrointestinal side effects. At 6 months from the initiation of semaglutide therapy, the patient’s body weight had decreased (−16 kg), together with systolic blood pressure (−18 mmHg) and TDD of insulin (−43%; with a predominant reduction in prandial insulin doses). Additionally, the HbA1c value decreased from 7.2% to 6.3% (−0.9%), with no increase in hypoglycemia. Overall, 6-month subcutaneous semaglutide therapy (in addition to insulin and metformin) was well-tolerated by the patient [103].

Wong et al. [121] presented the clinical cases of three patients with T1D and comorbid obesity who exhibited reduced insulin requirements and remarkable weight loss after the initiation of GLP-1 RA and pramlintide (amylin analog) combination therapy (in addition to intensive lifestyle modifications). With regard to GLP-1 RA therapy, two of these patients were treated with semaglutide, whereas one patient was treated with dulaglutide. The dulaglutide-treated patient was not considered, as this clinical case was out of the scope of this review.

A 32-year-old man with T1D (treated with an insulin pump), microalbuminuria, OSA, restless legs syndrome and class 3 obesity (BMI: 47.43 kg/m^2^) experienced a body weight loss of 20.9 kg (−16.1% of total body weight; −15.2% of body fat percentage) over 10 months, while he was on combination therapy with once-weekly subcutaneous semaglutide (at a dose of 0.25 mg/week, increased to 1 mg/week during a 2-month period) and pramlintide (15 mcg pre-meal subcutaneous injections, three times a day). The other case concerned a 49-year-old woman with T1D (treated with an insulin pump), hypertension, hypothyroidism, depression and class 1 obesity (BMI: 30.9 kg/m^2^; body fat percentage: 42.3%), who experienced a body weight loss of 14.6 kg (−17.9% of total body weight; −5.0% of body fat percentage) over 6 months, while she was on once-weekly subcutaneous semaglutide (at a dose of 0.5 mg/week) and pramlintide (15 mcg pre-meal subcutaneous injections, three times a day; after 6 months, the pramlintide dose was increased to 30 mcg pre-meal injections, three times a day). Both patients reported decreased insulin requirements and reductions in HbA1c values compared to baseline (HbA1c reductions: −0.2% and −0.9%, respectively) after combination therapy with semaglutide and pramlintide. No significant side effects were reported in both patients after the initiation of GLP-1 RA and pramlintide combination therapy [121]. These findings may suggest the possible existence of synergistic weight loss and glucose-lowering effects of semaglutide and pramlintide, although prospective studies are needed to confirm this hypothesis.

Gad and Malik [122] reported the case of a 34-year-old woman with a 23-year history of T1D who presented with weight gain (about 10 kg) over 3 to 4 years, overweight (BMI: 26.9 kg/m^2^), an HbA1c value of 8.3%, along with increased glycemic variability (as evidenced by a CV of glucose value of 48.2%). At the time of presentation, the patient was on MDI insulin therapy based on the use of basal insulin glargine U300 (at a daily dose of 14 U) plus prandial insulin lispro (the latter administered at each meal, at a dose depending on carbohydrate counting, insulin-to-carbohydrate ratio and insulin sensitivity factor). The patient had no evidence of microalbuminuria, retinopathy or neuropathy. Once-weekly subcutaneous semaglutide was prescribed at a starting dose of 0.25 mg/week for the initial 2 weeks. The weekly dose of semaglutide was then increased to 0.5 mg for the subsequent 2 weeks. Afterwards, the weekly dose of semaglutide was up-titrated to a maintenance dose of 1.0 mg. Semaglutide therapy for 2 months resulted in a body weight reduction of 12 kg, a percent body fat reduction of 15%, and a visceral fat reduction of 7%. After 2 months of semaglutide therapy, TAR > 250 mg/dL was reduced from 11% to 4%, although this reduction was accompanied by a slight increase in TAR 181–250 mg/dL (from 19% to 22%) and occurred partly at the expense of increases in TBR 54–69 mg/dL (from 8% to 10%) and TBR < 54 mg/dL (from 3% to 5%). TIR 70–180 mg/dL remained unchanged after 2 months of semaglutide therapy (59%). GMI, average glucose and CV of glucose decreased from 6.9% to 6.6%, from 150 mg/dL to 139 mg/dL, and from 48.2% to 44.6%, respectively. These changes were accompanied by a reduction in the doses of insulin glargine (−2 U) and insulin lispro (about −2 U based on carbohydrate counting). Overall, 2-month semaglutide therapy was well-tolerated by the patient [122].

Interestingly, Gong and Wentworth [123] reported the resolution of symptoms of severe binge eating disorder after semaglutide therapy in a 27-year-old woman with class 1 obesity (BMI: 30.1 kg/m^2^) and long-standing T1D, who was treated with basal-bolus insulin therapy starting from disease diagnosis (at 10 years of age). The patient suffered from anxiety (treated with monthly psychological counselling in combination with vortioxetine therapy) and severe binge eating disorder, as she reported daily binges of large amounts of food that were distressing and unrelated to hunger. The CGM report showed severe hyperglycemia that worsened during overnight binges. She scored 39/46 on the Binge Eating Scale (BES) and reported significant weight-related distress contributing to high depression scores on the 21-item Depression Anxiety and Stress Scale (DASS-21). She started once-weekly subcutaneous semaglutide for the treatment of obesity, at a starting weekly dose of 0.25 mg, which was then increased to 0.5 mg after 4 weeks. After one month of semaglutide therapy (at a weekly dose of 0.25 mg), the patient’s body weight had already decreased from 82 kg to 77 kg (−5 kg) and TIR 3.9–10.0 mmol/L (70–180 mg/dL) had increased from 9% to 44%, despite similar adherence to insulin therapy (although the daily insulin doses had decreased). There was also a reduction in the BES score and in the DASS-21 score. After 2 months, the patient had to interrupt the use of semaglutide due to drug shortage. One month later, she experienced recurrent symptoms of binge eating disorder, weight regain (body weight: 84 kg; BMI: 30.8 kg/m^2^) and deterioration of glucose control (as evidenced by a GMI value of 10.2%). During the subsequent 8 months, the patient obtained a sporadic supply of semaglutide and used the medication at an average weekly dose of 0.5 mg. Despite the intermittent use of semaglutide, she experienced resolution of severe binge eating disorder symptoms and improvement of depression, stress and anxiety symptoms [123].

## 4. Clinical Studies on the Use of Semaglutide in LADA

Our literature search regarding the use of semaglutide in LADA identified only a case report published by Da Porto et al. [124]. The authors reported good long-term glycemic control and beta-cell preservation in a patient with LADA who received early treatment with semaglutide and metformin. The patient was a 36-year-old woman with a past medical history of autoimmune thyroiditis and gestational diabetes mellitus that required only dietary management. The patient presented with polyuria and recurrent genital infections. She also reported recent unintentional weight loss. Her BMI was 20 kg/m^2^ and her waist circumference was 76 cm. She had a familiar history of T2D and was physically active. Laboratory tests showed a random blood glucose value of 315 mg/dL, normal anion gap and bicarbonate values, an HbA1c value of 12.3% (111 mmol/mol), absence of blood and urinary ketones, positivity of GAD antibodies and antibodies against intracellular epitopes of the tyrosine-phosphatase 2, as well as negativity of zinc transporter 8 antibodies. Genetic testing for MODY (maturity-onset diabetes of the young) was negative. Such clinical and laboratory findings led to a diagnosis of LADA, which was associated with preserved fasting C-peptide values (0.65 ng/mL). The patient was initially treated with metformin (at a dose of 1000 mg twice a day) and basal insulin (insulin glargine U300, at a dose of 10 units/day). Blood glucose levels gradually normalized during the first 5 weeks of treatment, although the patient experienced episodes of fasting hypoglycemia. Hence, basal insulin was interrupted and replaced with once-weekly subcutaneous semaglutide, which was started at an initial weekly dose of 0.25 mg for the first 4 weeks and subsequently titrated to a weekly dose of 0.5 mg. During a 5-year follow-up period, the patient underwent periodic 180-min mixed meal tolerance tests (MMTTs) to monitor the residual beta-cell function over time. During the 5-year follow-up period, the patient maintained good glycemic control until 36 months, when there was a mild increase in the Hb1Ac value (7.1%) and the semaglutide dose was therefore titrated to 1 mg/week. The patient exhibited a preserved beta-cell function up to 60 months after the initial diagnosis of LADA [124]. Supplementary Table S1 lists the clinical studies on the use of semaglutide in patients with autoimmune diabetes.

## 5. Clinical Studies on the Use of Tirzepatide in T1D

### 5.1. Retrospective Cohort Studies

A single-center retrospective observational study conducted by Akturk et al. [125] evaluated the safety and efficacy of tirzepatide in 26 adult patients with T1D over an observation period of 8 months. The mean age of the study participants was 42 years (age range: 28–56 years), with a mean BMI of 36.7 kg/m^2^. The starting weekly dose of tirzepatide for all patients was 2.5 mg, even though dose adjustments varied among patients and the tirzepatide doses were up-titrated based on the individual glycemic and/or weight loss goals. Changes from baseline in HbA1c, body weight, TDD of insulin and CGM metrics were expressed as least squares means. The authors observed a significant reduction in the HbA1c value by 0.45% at 3 months, which was sustained over the 8-month period. Moreover, there was a significant body weight loss over the 8-month period. In particular, the percent change in body weight from baseline was −3.4% at 3 months and −10.5% at 6 months, although no further body weight loss was observed after 6 months. The total daily insulin dose decreased by 24.3 IU/day at 8 months. With regard to CGM metrics, there was a significant increase in TIR 70–180 mg/dL by 12.6% at 3 months, which was sustained over the 8-month period and accompanied by a significant increase in time in tight range (TITR) 70–140 mg/dL by 10.7% at 3 months and by 11.5% at 6 months. Moreover, there was a significant reduction in TAR > 180 mg/dL by 12.6% at 3 months. There was no change in TBR (reported as the percentage of time spent with interstitial glucose values below 70 mg/dL), whereas a severe hypoglycemic episode (*n* = 1) and severe constipation (*n* = 1) led to tirzepatide discontinuation in two patients. Furthermore, one patient experienced peroneal nerve palsy (foot drop) as a possible consequence of rapid weight loss. No DKA event was reported during the 8-month period [125].

Garg et al. [126] conducted a retrospective single-center real-world study in 62 adult patients with long-standing T1D and overweight/obesity who were prescribed tirzepatide (treated group) and followed for one year. Inclusion criteria for this analysis were the following: patients age between 18 and 80 years; use of tirzepatide for at least 3 months; BMI ≥ 27 kg/m^2^; intensive insulin therapy using either multiple daily injections or an insulin pump or a hybrid closed-loop (HCL) system; and use of a CGM sensor. The authors used for the data analysis a control group including 37 patients with overweight or obesity who were not using any other weight loss medication during the same period and were computer-frequency matched by age, duration of diabetes, gender, BMI, and glucose control. At baseline, gender distribution, mean age, ethnicity, duration of diabetes and HbA1c were similar in the two groups, although the mean body weight, BMI and TDD of insulin were higher in the tirzepatide group than in the control group. The starting weekly tirzepatide dose was 2.5 mg, with the mean weekly tirzepatide dose being 5.6 mg and 9.7 mg at 3 and 12 months, respectively. Body weight and BMI decreased significantly at each time point (3, 6, 9, and 12 months) in the tirzepatide group compared to the control group. In particular, the mean body weight decreased significantly by 21.4 lbs. (9.6%) at 3 months and by 46.5 lbs. (18.5%) at 12 months in the tirzepatide group. Moreover, the mean BMI decreased significantly by 3.4 kg/m^2^ at 3 months and by 6.5 kg/m^2^ at 12 months in the tirzepatide group. The mean HbA1c decreased significantly at all time points in the tirzepatide group compared to the control group by 0.50% at 3 months and by 0.67% at 12 months. Moreover, the mean TDD of insulin decreased significantly in the tirzepatide group compared to the control group at all time points, specifically by 26.1 U/day at 3 months and by 22.8 U/day at 12 months. With regard to CGM metrics, there were significant decreases in mean CGM glucose in the tirzepatide group compared to the control group at 3 months (−16.8 mg/dL), 6 months (−19.1 mg/dL) and 12 months (−23.5 mg/dL). There were significantly greater increases in mean TIR 70–180 mg/dL in the tirzepatide group compared to the control group at 3 months (+10.5%) and at 6 months (+10.8%). There were significantly greater decreases in mean TAR > 180 mg/dL in the tirzepatide group compared to the control group at 3 months (−11.0%), at 6 months (−11.8%) and at 9 months (−9.3%). There were also significantly greater decreases in mean SD of glucose in the tirzepatide group compared to the control group at 6 months (−9.5 mg/dL), at 9 months (−9.9 mg/dL) and at 12 months (−11.0 mg/dL), even though no significant difference in CV of glucose was observed between the tirzepatide group and the control group at any time point. Importantly, TBR < 70 mg/dL did not change significantly in either group at any time point, and there was no significant difference in the change in TBR < 70 mg/dL between the two groups. There were no reported hospitalizations due to severe hypoglycemia or DKA in both groups [126].

Karakus et al. [127] conducted a pilot, single-center, retrospective observational study to explore the role of tirzepatide as a non-insulin adjunct therapy in adults with T1D who were using an AID system. The authors analyzed changes in body weight, insulin requirements and markers of glucose control over an 8-month period after the initiation of tirzepatide therapy, with data collected at different time intervals (0–2 months, 2–3 months, 3–6 months, and 6–8 months) and compared to baseline. Eleven adult patients with long-standing T1D, who were using the Tandem t:slim X2 insulin pump with Control-IQ technology (Tandem Diabetes Care, Inc., San Diego, CA, USA) and received tirzepatide as a non-insulin adjunct therapy, were included in the analysis. The median age of the study participants was 37 years, whereas the median BMI was 39.6 kg/m^2^ and the median diabetes duration was 24 years. The starting weekly dose of tirzepatide for all patients was 2.5 mg, and the doses were up-titrated and adjusted by physicians based on the individual patients’ weight loss and/or glycemic goals and adverse drug events. There was a significant reduction in TDD of insulin from a median of 73.9 units/day at baseline to 51.7 units/day at 0–2 months interval (corresponding to a 30% reduction in TDD of insulin within 2 months after the initiation of tirzepatide therapy), with less pronounced subsequent reductions: median of 46.2 units/day at 2–3 months interval; median of 45.3 units/day at 3–6 months interval; and median of 41.8 units/day at 6–8 months interval. Notably, there was a significant reduction in median values of both basal insulin and bolus insulin doses within the first 2 months from baseline (31% reduction in basal insulin dose; 43% reduction in bolus insulin dose), and this reduction remained significantly sustained over 8 months: median basal insulin, from 47 units/day at baseline to 25.6 units/day at 6–8 months interval; median bolus insulin, from 31.4 units/day at baseline to 13.0 units/day at 6–8 months interval. Of note, the largest percent reduction in median bolus insulin dose was seen within the first 2 months after the initiation of tirzepatide therapy (43%). TDD of insulin expressed as units/kg/day (*n* = 8) also decreased significantly from a median of 0.53 units/kg/day at baseline to a median of 0.40 units/kg/day at 6 months from baseline (corresponding, approximately, to a 25% reduction compared to baseline). With regard to bolus insulin, there was a significant reduction in the doses of both user-initiated insulin boluses and automated correction insulin boluses during the first 2 months, and this reduction remained significantly sustained over 8 months. Similarly, there was a significant reduction in the number of automated correction insulin bolus counts during the 8-month period. Carbohydrate entry into the insulin pump decreased during the 8-month period after the initiation of tirzepatide therapy, with mean daily carbohydrate consumption declining from 120 g to 70 g at 0–2 months, 81 g at 2–3 months, 66 g at 3–6 months, and 45 g at 6–8 months.

Body weight and BMI (*n* = 9) decreased significantly from a median of 114.3 kg and 39.6 kg/m^2^ at baseline to a median of 105.7 kg and 36.3 kg/m^2^ at 6 months, respectively. Moreover, median HA1c values decreased from 7.0% to 6.3% after tirzepatide therapy, although this change was not statistically significant. With regard to CGM metrics, there was a significant increase in TIR 70–180 mg/dL within the first 2 months after the initiation of tirzepatide therapy, and this increase remained significantly sustained throughout the subsequent time intervals: median TIR 70–180 mg/dL, from 69.8% at baseline to 75.6% at 6–8 months. The ambulatory glucose profile (AGP) showed an improvement in interstitial glucose values throughout the day after tirzepatide therapy, which was more pronounced during daytime and was also accompanied by lower postprandial glucose values. Importantly, there was no significant change in TBR < 70 mg/dL between baseline and all the subsequent analyzed time intervals (median TBR values at baseline, 0–2 months, 2–3 months, 3–6 months, and 6–8 months: 0.8%, 0.6%, 0.6%, 0.6%, and 0.7%, respectively) [127].

Rivera Gutierrez et al. [128] conducted a retrospective review of electronic medical records of adults with T1D who were prescribed tirzepatide for the treatment of overweight and obesity at Mayo Clinic (between 1 June 2022 and 31 October 2023). The authors categorized weekly subcutaneous tirzepatide dosages as “low dose” (2.5–5.0 mg), “medium dose” (7.5-10.0 mg), and “high dose” (12.5–15.0 mg). In total, 51 patients were included in the study. All the study participants had used tirzepatide for at least 3 months. The primary endpoint was total body weight loss percentage (TBWL%) at the last follow-up (last time point at which data were documented or tirzepatide therapy was discontinued). At baseline, most subjects were female (*n* = 30; 58.8%) and had class 3 obesity (*n* = 21; 41.2%). The most prevalent adiposity-related condition was hyperlipidemia (*n* = 41; 80.4%), and most patients had long-standing T1D. Most patients were on insulin pump therapy (*n* = 40; 78.4%) and used a CGM device (*n* = 46; 90.2%). The median time of follow-up was 8.0 months. With regard to tirzepatide dose, most patients (41.2%) achieved a medium dose, and about one-third of patients (35.3%) achieved a high dose.

At the last follow-up, there was a significant reduction in median TBWL%, which amounted to −8.5%. By the last follow-up, 76.5% of patients (*n* = 39) achieved at least 5% of TBWL, while all patients with available data at 12 months of follow-up achieved at least 5% of TBWL. The reduction in TBWL% was significant at all time points (3 months, −6%; 6 months, −9%; 9 months, −10%; 12 months, −12.2%).

At the last follow-up, there was a significant reduction in the median HbA1c by 0.9%. This improvement in HbA1c values was mirrored by significant changes in CGM metrics among CGM users, namely: a significant increase in median TIR 70–180 mg/dL (from 51.0% at baseline to 69.0% at the last follow-up) and a significant decrease in median TAR > 180 mg/dL (from 48.0% at baseline to 29.0% at the last follow-up). There was no significant change in median TBR < 70 mg/dL among CGM users. At the last follow-up, there was a significant reduction in TDD of insulin (−25 units, corresponding to a 31.6% reduction). In particular, the authors observed a precipitous (and statistically significant) decrease in TDD of insulin (to almost half of the daily insulin requirements) during the first 6 months of follow-up. Similar trends of reduction were also observed (particularly during the first 6 months of follow-up) for total daily basal insulin and total daily bolus insulin. Tirzepatide therapy was also associated with a significant decrease in total cholesterol, LDL cholesterol, triglycerides, alanine aminotransferase, and diastolic blood pressure.

Almost one-third of patients (*n* = 15; 29.4%) reported at least one side effect of tirzepatide, with the most common being nausea (*n* = 7; 13.7%). Only 5.9% of patients (*n* = 3) reported moderate to severe gastrointestinal side effects that caused medication discontinuation, whereas 7.8% of patients (*n* = 4) reported moderate gastrointestinal symptoms requiring tirzepatide dose de-escalation. Moreover, 7.8% of patients (*n* = 4) reported hypoglycemia, although none required assistance, hospitalization or medication discontinuation. There were no reported cases of DKA.

Over a median follow-up period of 8 months, the use of tirzepatide in adults with T1D was therefore associated with significant weight loss, along with significant reduction in daily insulin requirements and improvement of glucose control and cardiometabolic parameters, without reported serious adverse events (including severe hypoglycemia and DKA) [128].

### 5.2. Case Series and Case Reports

Sharma et al. [129] first described the case of a 36-year-old woman with T1D on a closed-loop insulin infusion system (Tandem t:slim X2 insulin pump with Control-IQ technology; Tandem Diabetes Care, Inc., San Diego, CA, USA) and tirzepatide therapy who experienced DKA after closed-loop insulin infusion set failure in the setting of volume depletion. The patient, who was initially treated only with the closed-loop insulin infusion system (with a predictive algorithm protecting against hyperglycemia and hypoglycemia), was prescribed tirzepatide due to the concomitant presence of class 1 obesity (BMI: 32.2 kg/m^2^) associated with poorly controlled diabetes (HbA1c: 9.4%). The exact tirzepatide dose and titration scheme were not specified in the manuscript. At 3 months from tirzepatide initiation, the patient had lost 50 lbs. (BMI: 25.3 kg/m^2^), but reported severe nausea and heartburn related to tirzepatide therapy. Given the ongoing weight loss, the patient decided to continue using tirzepatide despite the medical advice to stop or decrease the drug dose. The patient presented to the emergency room 4 days after the last tirzepatide injection, reporting acute worsening of heartburn, nausea, vomiting, and minimal oral intake for 3 days. Home urine ketone testing was positive. The patient was alert and afebrile. She had tachycardia, tachypnea, Kussmaul breathing and dry oral mucosa, suggesting volume depletion. At that time, the patient’s TDD of insulin was 30 units, while the patient’s TDD of insulin was closer to 70 units before tirzepatide initiation. The closed-loop insulin infusion system showed intermittent insulin delivery suspensions due to intermittent hypoglycemic episodes reported by the CGM sensor (Dexcom G6). However, a point-of-care blood glucose testing showed a glucose level of 289 mg/dL, which was comparable to the serum glucose level detected with laboratory tests (322 mg/dL), whereas the concurrent CGM reading showed an interstitial glucose level of 40 mg/dL. Further laboratory tests confirmed the presence of high-anion-gap metabolic acidosis. The patient was therefore diagnosed with DKA based on elevated serum glucose values associated with high-anion-gap metabolic acidosis and a history of positive urine ketones on home urine testing. Physical examination and laboratory tests did not show findings suggestive of infection, while volume depletion was attributed to the gastrointestinal side effects of tirzepatide. The insulin pump and CGM sensor were removed, and the patient was treated with intravenous insulin and fluid therapy. Serum glucose values improved (143 mg/dL) and the anion gap normalized within 8 h, while normalization of serum bicarbonate values occurred within 72 h. Afterwards, the patient was first transitioned to MDI insulin therapy with insulin pens and then to the closed-loop insulin infusion system (Tandem t:slim X2 insulin pump with Control-IQ technology plus Dexcom G6 CGM sensor) at pre-admission settings. Glucose values remained within the optimal range for the subsequent 24 h and until the outpatient follow-up visit 10 days later, while tirzepatide was definitively discontinued. The substantial decrease in TDD of insulin observed after the initiation of tirzepatide therapy was attributed to the weight loss and decreased insulin resistance induced by tirzepatide [129].

It is important to highlight that the aforementioned episode of DKA was a direct consequence of poor CGM accuracy and subsequent insulin pump infusion set failure, as the algorithm of the closed-loop insulin infusion system depends on the accuracy of the CGM sensor. In this regard, the authors hypothesized that side effects of tirzepatide therapy (particularly vomiting) precipitated volume depletion, which, in turn, caused intermittent and inaccurate CGM sensor readings and subsequent intermittent suspensions of insulin delivery by the insulin pump due to false CGM hypoglycemia readings [129]. Indeed, conditions characterized by volume depletion induced by fluid loss can compromise the accuracy of CGM sensors for the measurement of interstitial fluid (ISF) glucose concentrations, as the decrease in intravascular water content leads to a compensatory mobilization of fluid and electrolytes from the interstitial space to the intravascular compartment [130], which results in ISF glucose concentration changes.

Mendoza and Parsiani [131] reported the case of a 23-year-old woman with T1D since the age of 10 years who was referred to the outpatient clinic due to insulin resistance and increasing insulin requirements. The patient was using CGM and insulin pump therapy (Omnipod 5) with hybrid closed-loop technology in automated mode (100% of the time), which remained consistent during the subsequent weeks. The patient’s most recent HbA1c value was 7.4%, and her BMI was indicative of class 2 obesity (38 kg/m^2^). The patient’s body weight had increased by about 40 pounds in the last year. Exercise was limited due to a busy schedule (less than 10 thousand steps per day). The patient was using an average daily dose of basal and prandial insulin of 55.4 units and 26.5 units, respectively (TDD of insulin: 81.9 units). The month earlier, she had started extended-release metformin (titrated to 1000 mg/day) to reduce insulin resistance and insulin requirements and improve glucose control. With regard to CGM metrics, GMI was 8.7%, TIR 70–180 mg/dL was only 31%, whereas TAR 181–250 mg/dL was 32%, TAR > 250 mg/dL was 37%, and TBR 54–69 mg/dL and TBR < 54 mg/dL were 0%. Thus, the patient was prescribed once-weekly subcutaneous tirzepatide at an initial weekly dose of 2.5 mg, which was titrated to 5 mg after 4 weeks and to 7.5 mg after the subsequent 4 weeks. Within the first month of tirzepatide therapy, the patient experienced a weight loss of 5 pounds. By week 9, the patient’s weight decreased further (−7 lbs.; BMI: 36 kg/m^2^), and her HbA1c decreased to 6.9%. By week 12, the patient reported a 24% decrease in daily carbohydrate consumption (from an average of 119.5 g to 90.9 g), although weight data were not available at this time. Moreover, at 12 weeks, the TDD of insulin decreased to 57.6 units, TIR 70–180 mg/dL increased to 61%, and TAR > 250 mg/dL decreased to 7% (vs. 81.9 units, 31%, and 37%, respectively, at baseline). During the course of the 12-week tirzepatide therapy, the patient continued to use metformin at a daily dose that varied over time (ranging from 1000 mg to 2000 mg). The main side effects of tirzepatide therapy were mild transient nausea (experienced with the 2.5 mg and 7.5 mg weekly doses), transient vomiting (experienced with the 7.5 mg weekly dose for about 2 days after drug injection), and transient hypoglycemic episodes (around the third week on the 2.5 mg weekly dose). However, hypoglycemic episodes were not long enough to affect the patient’s TBR and disappeared after the adjustment of the insulin-to-carbohydrate ratios [131].

Ahmed et al. [132] reported the case of a 38-year-old man with a 16-year history of T1D presenting with poor glucose control while he was on MDI insulin therapy (with prandial insulin lispro-aabc plus basal insulin degludec) and wearing a CGM sensor. The patient’s glucose levels ranged between 75 mg/dL and 200 mg/dL, with intermittent hyperglycemic episodes (>200 mg/dL). The patient also suffered from hypertension, steatotic liver disease, combined hyperlipidemia, stage 2 chronic kidney disease, renal hyperparathyroidism, diabetic nephropathy and macroalbuminuria. The patient’s body weight was 126.5 kg, while his systolic blood pressure and diastolic blood pressure values were reported to be around 165 mmHg and 100 mmHg, respectively. Moreover, the most recent urine albumin–creatinine ratio (uACR) value at the time of presentation was 700 mg/g. Therefore, the patient was prescribed tirzepatide at a starting weekly dose of 2.5 mg. During the subsequent 30 days, the patient’s daily glucose levels remained consistently within the range 100–150 mg/dL, with only a few hypoglycemic episodes. Thus, the weekly tirzepatide dose was increased to 5 mg at 30 days from tirzepatide therapy initiation. Afterwards, the daily glucose levels remained consistently within the range 80–140 mg/dL, without significant hyperglycemic episodes and with rare hypoglycemic episodes. The weekly tirzepatide dose was then increased to 7.5 mg after about 4 weeks from the initiation of 5-mg weekly tirzepatide therapy. During this period, the patient also experienced a reduction in TDD of insulin (from about 45 units/day pre-tirzepatide therapy to about 46 units/day). After about 2 months from the initiation of 7.5-mg weekly tirzepatide therapy, the patient missed the tirzepatide injections for about 30 days due to a family emergency, thus experiencing a weight gain of 1.5 kg. Subsequently, the patient resumed tirzepatide therapy at a low maintenance weekly dose of 5 mg. At the last follow-up visit, which was performed at 6 months from the initiation of tirzepatide therapy, the patient showed a body weight of 104.6 kg (−21.9 kg vs. pre-tirzepatide therapy), along with decreased values of HbA1c (5.8%), uACR (117 mg/g), and systolic and diastolic blood pressure (about 130/80 mmHg vs. 165/100 mmHg pre-tirzepatide therapy), thus suggesting the potential efficacy of tirzepatide in improving glucose control, treating hypertension and counteracting the progression of diabetic kidney disease [132].

## 6. Clinical Studies on the Use of Tirzepatide in LADA

Our literature search regarding the use of tirzepatide in LADA identified only a post-hoc analysis of SURPASS clinical trials (SURPASS-2, SURPASS-3, SURPASS-4, SURPASS-5) conducted by Peters et al. [133]. The post-hoc analysis aimed to assess changes in HbA1c and body weight associated with tirzepatide therapy in GADA-positive vs. GADA-negative participants with a clinical diagnosis of T2D. Patients with clinically diagnosed T2D but exhibiting GADA positivity were considered to be likely affected by LADA. The post-hoc analysis based on pooled data from the aforementioned clinical trials was performed using mixed-model repeated measures from the efficacy analysis set, adjusting for study and baseline covariates, including sex, age, BMI, HbA1c and GADA status. Study participants were randomly assigned to receive once-weekly tirzepatide (5 mg, 10 mg or 15 mg), once-weekly semaglutide (1 mg) (SURPASS-2), insulin degludec (SURPASS-3), insulin glargine (SURPASS-4), or a volume-matched placebo in a single-dose pen (SURPASS-5). Overall, 3791 participants with confirmed GADA status were included in this analysis: 3671 (96.8%) were GADA-negative, while 120 (3.2%) tested positive for GADA. Of the 120 participants who were GADA-positive, 76 (63.3%) had low GADA levels (defined by GADA concentration ≤ 200 IU/mL and ≥5 IU/mL) and 44 (36.7%) had high GADA levels (defined by GADA concentration > 200 IU/mL). Baseline characteristics were similar between the two groups, except for BMI and serum triglyceride concentration, which were slightly lower in GADA-positive patients compared to GADA-negative patients: mean BMI, 32.2 kg/m^2^ vs. 33.6 kg/m^2^; mean triglycerides, 169.2 mg/dL vs. 188.1 mg/dL.

The change from baseline in HbA1c and body weight over time was expressed as least squares mean (LSM) ± standard error (SE). Both GADA-positive and GADA-negative patients achieved significant reductions from baseline in HbA1c values over time. At week 40/42, both GADA-positive and GADA-negative patients achieved significant HbA1c reductions, with slightly greater reductions in HbA1c observed in GADA-negative participants than in GADA-positive participants (−2.32% vs. −2.11%; estimated between GADA subgroup differences: 95% confidence interval [95% CI], 0.21% [0.03–0.39%]; *p* = 0.024). Moreover, significant reductions from baseline in HbA1c were observed in tirzepatide-treated GADA-positive patients, regardless of having low or high GADA levels over time; at week 40/42, HbA1c reductions were −2.17% in GADA-positive participants with low GADA levels and −2.01% in GADA-positive participants with high GADA levels. In addition, tirzepatide-treated GADA-positive patients from SURPASS-2 and SURPASS-4 achieved improvements in glucose control irrespective of baseline fasting C-peptide levels.

Both GADA-positive and GADA-negative patients showed significant reductions from baseline in body weight over time. Yet, at week 40/42, there was no significant difference in body weight reductions between GADA-negative patients and GADA-positive patients (−9.6 kg [−10.2%] vs. −9.2 kg [−10.4%]; estimated treatment differences: 95% CI, 0.38 kg [−0.99 to 1.75 kg]; *p* = 0.588). The significant reductions from baseline in body weight were observed in tirzepatide-treated GADA-positive patients regardless of having low or high GADA levels over time; at week 40/42, body weight reductions with tirzepatide were −9.1 kg (−9.8%) in GADA-positive patients with low GADA levels and −9.5 kg (−11.0%) in GADA-positive patients with high GADA levels.

A pooled analysis of SURPASS-2 and SURPASS-4 clinical trials showed that baseline HOMA2-B (homeostasis model assessment of beta-cell function; calculated with fasting C-peptide) increased significantly from baseline over time in both GADA-positive and GADA-negative patients during tirzepatide therapy (by 62.4% and 97.0%, respectively), with improvement in HOMA2-B being significantly greater among GADA-negative patients than in GADA-positive patients at week 40/52 (estimated difference vs. GADA-positive patients: 95% CI, 21.3% [7.6–36.8%]; *p* = 0.002). Moreover, HOMA2-IR (homeostasis model assessment of insulin resistance; calculated with fasting C-peptide) decreased significantly from baseline in both GADA-positive and GADA-negative patients during tirzepatide therapy by 27.2% and 16.9%, respectively, with the improvement in HOMA2-IR being significantly greater among GADA-positive patients than in GADA-negative patients at week 40/52 (estimated difference vs. GADA-negative patients: 95% CI, 14.3% [1.9–28.2%]; *p* = 0.023).

Overall, hypoglycemia was reported in 151 out of 3678 GADA-negative patients compared to 12 out of 120 GADA-positive patients (6 patients with high GADA levels and 6 patients with low GADA levels). However, seven of these 12 GADA-positive patients were on insulin therapy, while none of these patients were concomitantly using sulfonylureas. Furthermore, there was no reported case of severe hypoglycemia in GADA-positive patients [133]. Thus, this post-hoc analysis represents the first demonstration of substantial reductions in HbA1c and body weight achieved with the novel once-weekly dual GIP/GLP-1 RA tirzepatide in adult GADA-positive patients who were previously diagnosed with T2D and who are likely affected by LADA. Interestingly, the study also suggests that the improvement in glucose control observed after tirzepatide therapy in this population may be related to the tirzepatide-mediated increase in beta-cell function and reduction of insulin resistance (as indicated by the increased values of the marker of beta-cell function HOMA2-B and by the decreased values of the marker of insulin resistance HOMA2-IR) [133].

### Use of Tirzepatide in a Patient with Mitochondrial Diabetes Mellitus (MDM) and GADA Positivity

By inserting the keywords “type 1 diabetes” and “tirzepatide” in the MEDLINE/PubMed database, we identified a case report describing the use of tirzepatide in a patient with mitochondrial diabetes mellitus (MDM) associated with GADA positivity. This case was published by Yoshihiko Suzuki, who described the successful use of tirzepatide in a 67-year-old man affected by MDM [134], a rare form of diabetes mellitus caused by mutations in mitochondrial DNA and often associated with other comorbidities, such as GADA positivity [135]. The patient in question was affected by MDM with a mutation at position 3271. He also had a past history of transient GADA positivity and autoimmune pancreatitis [134]. Even though autoimmune pancreatitis went into spontaneous remission, the patient experienced poor post-pancreatitis glycemic control. Thus, the patient, who had a history of treatment with oral glucose-lowering medications for several years, was prescribed tirzepatide. After 5 months of tirzepatide therapy, his HbA1c decreased from 7.0% to 6.4% and his serum C-peptide decreased from 1.04 ng/mL to 0.89 ng/mL. The decrease in serum C-peptide values was interpreted as a likely consequence of the reduction in insulin resistance. Other notable findings observed after tirzepatide therapy were a body weight loss of 3.3 kg and discontinuation of glimepiride. Moreover, GADA remained negative, and autoimmune pancreatitis did not relapse over the course of tirzepatide therapy [134]. Supplementary Table S2 lists the clinical studies on the use of tirzepatide in patients with autoimmune diabetes.

## 7. Clinical Studies Evaluating and Comparing the Use of Semaglutide and Tirzepatide in Patients with T1D and Overweight/Obesity (Including Genetic Forms of Obesity)

Snell-Bergeon et al. [136] performed a retrospective analysis to assess the safety and efficacy of off-label semaglutide and tirzepatide use in adult patients with T1D over a period of 1 year. This retrospective chart review included 100 patients who were prescribed semaglutide (*n* = 50) or tirzepatide (*n* = 50) and 50 controls frequency matched for age, sex, BMI, HbA1c and diabetes duration, and who did not receive any weight loss drugs during the study period. Data were collected before the initiation of weight loss drugs (baseline) and for up to 1 year for each patient. Almost all patients who were prescribed semaglutide (*n* = 36; 72%) and tirzepatide (*n* = 43; 86%) were affected by obesity, while 11 (22%) semaglutide-treated patients and 7 (14%) tirzepatide-treated patients were affected by overweight. Baseline matching characteristics (age, sex, BMI, HbA1c and diabetes duration) were similar between the cases and the controls, with no differences observed in matching variables between the cases and controls. All insulin pump users were using AID systems. Patients started on an initial weekly dose of 0.25 mg for semaglutide and of 2.5 mg for tirzepatide, as per clinical guidelines. The median weekly dose of semaglutide prescribed was 0.5 mg, while the median weekly dose of tirzepatide prescribed was 7.5 mg weekly.

At 12 months, BMI decreased by a least square mean of 3.0 kg/m^2^ and 7.5 kg/m^2^ in the semaglutide and tirzepatide groups, respectively. There was no significant change in BMI in the control group at any time point. At 12 months, body weight decreased by a least square mean of 19.2 lbs. (9.1%) and 49.4 lbs. (21.4%) in the semaglutide and tirzepatide groups, respectively. BMI, weight in pounds and weight percentage decreased significantly more in the tirzepatide group than in the control group at all time points from 3 to 12 months, and significantly more in the semaglutide group than in the control group at 6, 9, and 12 months. Importantly, the reduction in BMI, weight in pounds and weight percentage was significantly greater in the tirzepatide group than in the semaglutide group at all time points. At 12 months, 93% of tirzepatide users, 77% of semaglutide users and 14% of controls had lost 5% of their baseline body weight, while 87% of tirzepatide users, 47% of semaglutide users and none of the controls had lost 10% or more of their baseline body weight.

At 12 months, there was a significant reduction in the least square mean HbA1c of 0.54% in the semaglutide group and a reduction in the least square mean HbA1c of 0.68% in the tirzepatide group, with no change observed in HbA1c among controls (−0.04%). The reduction in HbA1c was significantly greater in the semaglutide group than in the control group and in the tirzepatide group than in the control group overall. Although there was no difference in the amount of reduction in HbA1c between the semaglutide and tirzepatide groups overall, there was a greater reduction in the tirzepatide group than in the semaglutide group at 6 months.

In the tirzepatide group, TDD of insulin as well as basal and bolus insulin doses (expressed either in U/day or U/kg/day) decreased significantly from baseline and to a greater extent than in the semaglutide and control groups at all time points (least square mean TDD of insulin at 12 months: tirzepatide group, −26.4 U/day [−0.13 U/kg/day]; semaglutide group, −4.1 U/day [0.02 U/kg/day]; control group, 3.9 U/day [0.02 U/kg/day]). With regard to the comparison between semaglutide and control groups in terms of daily insulin doses, only the bolus insulin dose at 6 months significantly differed between semaglutide users and controls (least square mean bolus insulin dose at 6 months: semaglutide group, −4.2 U/day; control group, 3.6 U/day). Changes in BMI, body weight and HbA1c did not differ significantly between groups based on the insulin delivery method (MDI insulin therapy, insulin pump/AID). Weight loss remained significantly greater in tirzepatide-treated patients than in semaglutide-treated patients, regardless of the insulin delivery method [136].

In summary, this was the first study comparing the efficacy of semaglutide and tirzepatide in improving glucose control and promoting weight loss in adults with T1D and comorbid overweight/obesity over 1 year. Notably, the study documented, for the first time, that tirzepatide promoted more than two times weight loss as compared to semaglutide, similarly to what has been reported in patients with overweight/obesity (with or without T2D) [137]; patients on semaglutide lost 19.2 lbs. (corresponding to 9.1% of body weight) and had a decrease in BMI of 3.0 kg/m^2^ at 1 year, while patients on tirzepatide lost 49.4 lbs. (corresponding to 21.4% of body weight) and had a decrease in BMI of 7.5 kg/m^2^ at 1 year [136]. Conversely, semaglutide and tirzepatide determined similar (clinically meaningful) improvements in glucose control (reduction in HbA1c of 0.54% and 0.68% after 12 months of treatment with semaglutide and tirzepatide, respectively). Nevertheless, it is important to note that the aforementioned study had various limitations besides its retrospective nature, such as the lack of information regarding the semaglutide and tirzepatide titration schedule used and the side effects experienced by patients [136]. Therefore, randomized controlled trials are needed to confirm these preliminary findings.

### 7.1. Use of Semaglutide and Tirzepatide in Adolescents and Young Adults (AYA) with T1D

In December 2022, the FDA approved the use of semaglutide for the treatment of obesity in adolescents aged 12 years and older [138].

Seetharaman and Cengiz [139] from the University of California, San Francisco (San Francisco, CA, USA) published a case series including adolescents and young adults (AYA) with T1D who received GLP-1 RA therapy (as an adjunct therapy to insulin). Patients were selected based on clinical needs, particularly concurrent obesity or suboptimal glucose control. The GLP-1 RAs used in this case series included semaglutide and tirzepatide. The case series (including eight patients) provided valuable real-world experience with semaglutide and tirzepatide and highlighted practical issues associated with the use of these medications in AYA with T1D. Interestingly, seven out of the eight patients had obesity. The remaining patient was an 18-year-old female with T1D for 5 years (managed with Omnipod insulin pump and Dexcom CGM sensor) who had a BMI of 22.9 kg/m^2^ (body weight: 60.9 kg; 134 lbs.) and was prescribed once-weekly subcutaneous semaglutide (at a starting dose of 0.25 mg/week) as an adjunct therapy for postprandial hyperglycemia and appetite regulation. She was previously using inhaled insulin (Afrezza^®^) at least once daily for managing postprandial hyperglycemia, but the medication was later denied by the patient’s insurance. The patient’s body weight was 63.2 kg (137 lbs.) at the time of semaglutide prescription and 57.4 kg (128 lbs.) after 4 months of semaglutide therapy. However, after about 4 months of semaglutide therapy, the patient was referred to a gastroenterology clinic for persistent diarrhea and weight loss and was subsequently diagnosed with isomaltase deficiency. Thus, the patient started digestive enzyme supplementation that improved her diarrhea. The weekly semaglutide dose was increased up to 2 mg/week. Afterwards, semaglutide was well-tolerated, the episodes of severe hypoglycemia decreased, glycemic variability decreased, and the patient continued to maintain good glucose control (as indicated by a GMI of 6.1%). This case highlights the importance of careful monitoring of GLP-1 RA side effects and differential diagnosis of underlying conditions that may mimic or deteriorate side effects of these medications.

Another interesting case described by the authors was that of a 14-year-old male with T1D for 6 years, who was managed with a t:slim insulin pump and Dexcom CGM sensor. The patient also suffered from obesity (BMI: 30 kg/m^2^; body weight: 84.4 kg) and insulin resistance. Therefore, he was prescribed once-weekly subcutaneous semaglutide (at a starting dose of 0.25 mg/week). After 2 months, he had lost 10 lbs., while he was also adopting lifestyle changes. He denied any side effects with semaglutide therapy. Semaglutide therapy also led to improved glucose control and reduced daily insulin requirements. Afterwards, semaglutide was interrupted due to rapid weight loss (44 lbs.) that occurred in 3.5 months. The patient developed restrictive eating behaviors, stating that he had a goal of losing 50 lbs. He was restricting his oral intake to 800 calories per day and running for about 30–60 min per day. Therefore, he was referred to the adolescent eating disorder clinic for a comprehensive assessment. This specific clinical case underscores the need for close monitoring of disordered eating behaviors in patients on GLP-1 RA therapy, especially in those who are already at risk for these behaviors. Hence, therapeutic benefits of GLP-1 RA should always be weighed against the potential psychological impact of these medications, particularly when they are prescribed as off-label drugs in AYA with T1D.

Most patients in this case series showed notable improvements in HbA1c (up to −2.2%), TIR (up to +27%) and average glucose levels (up to −50 mg/dL), as well as reductions in TDD of insulin (up to −0.67 U/kg/day) and mild to moderate weight loss (up to 20.5 kg, except for one patient) after treatment with semaglutide or tirzepatide (used for up to 16 months). With regard to side effects, a significant proportion of patients experienced common gastrointestinal side effects of GLP-1 RAs, such as nausea, vomiting and diarrhea, even when these medications were used at low doses. However, such symptoms generally improved after a few weeks, with some patients managing them with the use of histamine H2-receptor antagonists and/or antiemetic drugs. Gradual GLP-1 RA dose titration helped minimize these common gastrointestinal side effects. One patient (a 14-year-old female with a 3-year history of T1D, managed with an Omnipod insulin pump and a Dexcom CGM sensor) experienced an increase in hypoglycemic episodes, thus requiring a decrease in her daily insulin dose. Importantly, there were no reported episodes of DKA. None of the patients in the case series exhibited depression, suicidal thoughts, or mood issues. These findings suggest that the novel incretin analogs semaglutide and tirzepatide (administered as add-on treatment to insulin) have the potential to improve glucose control, reduce insulin requirements and promote weight management in AYA with T1D, without increasing the risk of serious adverse events [139].

### 7.2. Use of Semaglutide and Tirzepatide in Patients with T1D and Genetic Forms of Obesity

It has been documented that between 9% and 27% of patients using tirzepatide or semaglutide have little or no response to these weight loss medications (patients considered “non-responders to incretin therapies”), with lack of response being defined as less than 5% weight loss observed over an average treatment period of 72 weeks [140]. Absent or poor response to incretin therapies may be attributable to different factors, such as genetic factors (e.g., genetic variation at the incretin receptors associated with expression of non-functional incretin receptors) and gut microbiome alterations, among others [140].

In this regard, an exploratory, single-center, retrospective study conducted by Klein et al. [141] sought to assess the response to incretin therapies in patients with long-standing T1D and obesity likely related to genetic mutations. Adults with a BMI ≥ 40 kg/m^2^ and a history of early-onset severe obesity were eligible for genetic testing (performed through saliva or blood sample collection) aimed at screening 79 genes mainly associated with the leptin–melanocortin pathway, Bardet–Biedl syndrome (and related ciliopathies) and genes affecting energy balance. The genetic mutations screened were mainly associated with the functionality of hypothalamic regulatory centers, which represent sites of endogenous GLP-1 action and play a critical role in the regulation of energy intake and expenditure. The majority of patients in the “mutation cohort” were heterozygous for the mutations screened. Adults with T1D who had participated in the “Uncovering Rare Obesity” program (through which eligible subjects were tested for genetic forms of obesity) and used incretin analogs (including GLP-1 RAs and the dual GIP/GLP-1 RA tirzepatide) for the management of obesity were enrolled in this study. The control group included 15 individuals with obesity unlikely related to genetic mutations, namely: 3 patients who tested negative for a genetic mutation associated with obesity, and 12 adults with T1D who were not eligible for genetic testing but were followed for up to 8 months to assess the safety and efficacy of tirzepatide therapy for the management of hyperglycemia or obesity. Among 126 individuals with a BMI ≥ 40 kg/m^2^ who were seen for clinical care, only 23 (18.3%) were eligible for and consented to genetic testing. In turn, 18 (78.3%) out of these 23 patients tested positive for an obesity-related mutation(s), while the remaining 5 (21.7%) patients tested negative. Incretin therapies were used by 14 of the 18 patients who tested positive for an obesity-related mutation(s) and by 4 of the 5 patients who tested negative for an obesity-related mutation(s). However, in this latter group of patients using incretin therapies, only 11 out of the 14 mutation-carrying patients were analyzed as the “mutation cohort”, and only 3 out of the 4 mutation-negative patients were included in the control group, since 3 patients who were positive for a mutation(s) and one patient who was negative for a mutation(s) did not have analyzable data at 6 months from baseline. Among the 11 patients in the “mutation cohort”, 3 patients (27.3%) used semaglutide and 4 patients (36.3%) used tirzepatide, while the remaining 4 patients used liraglutide (*n* = 1) and dulaglutide (*n* = 3). Among the 15 patients in the control group, 13 patients (86.6%) used tirzepatide, while the remaining 2 patients used liraglutide (*n* = 1) and exenatide (*n* = 1). Data on dosages of GLP-1 RAs and tirzepatide over time in the two groups were not available. At baseline (defined as the date of initiation of incretin therapies), patients with obesity likely related to genetic mutations, as compared to controls, were younger (median age: 39.5 vs. 45.8 years), with a shorter duration of diabetes (median duration of diabetes: 12.8 vs. 24.0 years) and a higher BMI (median BMI: 43.0 vs. 38.7 kg/m^2^). Baseline use of technologies for type T1D management (such as CGM sensors and insulin pumps) and HbA1c values were similar between the two groups. As expected, baseline TDD of insulin was higher among adults with obesity likely related to genetic mutations than among controls (median TDD of insulin: 0.68 vs. 0.46 units/kg/day, respectively). The primary outcome of this study was percentage change in body weight and absolute change in HbA1c at 6 months from baseline across the two groups. Response to incretin therapy was defined based on the achievement of a body weight reduction equal to or greater than 5% at 6 months from baseline and/or on the achievement of an HbA1c reduction equal to or greater than 0.4% at 6 months from baseline. Of note, there was a non-significant lower absolute and relative change in body weight (mean: −5.75 kg vs. −8.65 kg; −4.78% vs. −8.57%) as well as a non-significant lower absolute change in HbA1c (mean: −0.28% vs. −0.43%) at 6 months among patients with obesity likely related to genetic mutations, as compared to the control group. Yet, there were significantly less subjects with obesity likely related to genetic mutations compared to subjects with obesity unlikely related to genetic mutations who met either HbA1c reduction of ≥0.4% or weight loss of ≥5% at 6 months from baseline (36.36% vs. 80.0%) [141]. Thus, this study documented a reduced efficacy of GLP-1 RAs and tirzepatide in terms of glucose-lowering and weight loss effects among patients with T1D and obesity likely related to genetic causes.

Supplementary Tables S1 and S2 include detailed information on the clinical studies evaluating and comparing the use of semaglutide and tirzepatide in patients with T1D and obesity (including genetic forms of obesity).

## 8. Discussion

### 8.1. Summary of the Current Evidence

The present narrative review comprehensively examined the existing studies investigating the use of the novel incretin analogs semaglutide and tirzepatide in patients with T1D (as non-insulin adjunct therapies at different stages of the disease) and in patients with LADA.

**Figure 1:**
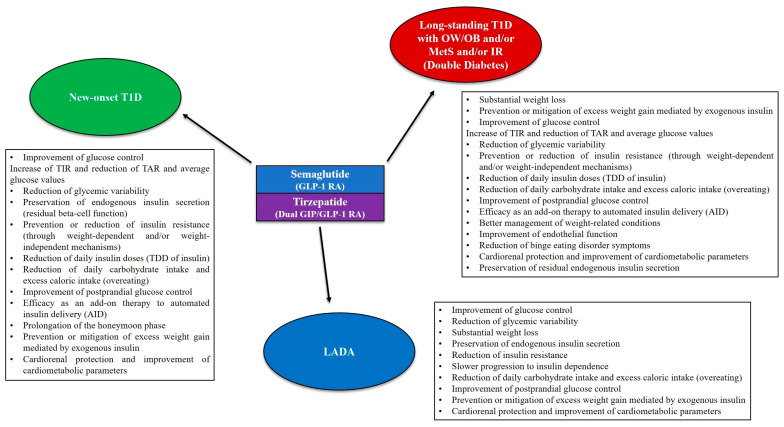
**Potential and established benefits of the novel incretin analogs semaglutide and tirzepatide in patients with T1D (at different stages of the disease), LADA and double diabetes associated with overweight/obesity.** Abbreviations: AID, automated insulin delivery; GIP, glucose-dependent insulinotropic polypeptide; GLP-1, glucagon-like peptide-1; IR, insulin resistance; LADA, latent autoimmune diabetes in adults; MetS, metabolic syndrome; OB, obesity; OW, overweight; RA, receptor agonist; T1D, type 1 diabetes; TAR, time above range; TDD, total daily dose; TIR, time in range.

Our review suggests that both semaglutide and tirzepatide may exert a series of beneficial effects in patients with T1D (at different stages of the disease) and LADA (Figure 1), as follows:

- Improvement of glucose control, as evidenced by reductions in HbA1c [in patients with T1D and LADA], fasting plasma glucose, average CGM glucose and TAR values, accompanied by the increase in TIR values (without significant changes in time spent in hypoglycemia [TBR]) [in patients with T1D]. In the studies examined in this review (excluding case series and case reports), the use of semaglutide and tirzepatide [administered at different doses and for varying durations (from 12 weeks to 50 months)] in T1D patients was associated with average/median reductions in HbA1c values of about 0.20–0.60% and 0.45–0.90%, respectively; on the other hand, the use of tirzepatide in patients with LADA (for 40/42 weeks) was associated with an average reduction in HbA1c values of 2.11%.
- Reduction of glycemic variability in patients with T1D (as evidenced by reductions in CV and SD of interstitial glucose values).
- Reduction of daily insulin doses (regarding particularly prandial insulin in some studies) in patients with new-onset and long-standing T1D.
- Reduction of daily carbohydrate intake in patients with long-standing T1D. Reduction of daily carbohydrate intake may be accompanied by reduction of excess caloric intake (overeating) and improvement of postprandial glucose control (decreased postprandial hyperglycemic episodes due to reduced prandial load with low dietary carbohydrate intake).
- Improvement of glucose control (without significant changes in TBR), reduction of daily insulin requirements, reduction of carbohydrate intake, substantial weight loss, and reduction of waist and hip circumferences in adult patients with established T1D using AID systems.
- Reduction of insulin resistance in patients with T1D and LADA.
- Preservation of endogenous insulin secretion and prolongation of the honeymoon phase in patients with new-onset T1D.
- Preservation and improvement of endogenous insulin secretion in patients with LADA.
- Substantial weight loss in patients with T1D/LADA and concomitant overweight/obesity. In the studies conducted in this population and examined in this review (excluding case series and case reports), the use of semaglutide and tirzepatide [administered at different doses and for varying durations (from 12 weeks to 50 months)] in T1D patients was associated with median/average reductions in body weight of about 5.0–10.6% and 8.5–21.4%, respectively; on the other hand, the use of tirzepatide in patients with LADA (for 40/42 weeks) was associated with an average reduction in body weight of 10.4%. These reductions in body weight were clinically meaningful, since the degree of weight loss achieved with semaglutide and tirzepatide in patients with autoimmune diabetes was substantially higher than the minimum weight loss required for metabolic health improvement [142].
- Potential improvement of symptoms of severe binge eating disorder, depression, stress and anxiety in patients with T1D and obesity: such potential benefits have been postulated based on a case report published by Gong and Wentworth [123]. This case report suggests semaglutide as a potential add-on treatment to insulin able to concomitantly improve glucose control, excess body weight and binge eating disorder symptoms. Although these findings need to be confirmed in clinical trials, they are particularly interesting since binge eating disorder and disordered eating behaviors can contribute to inadequate glucose control and weight management in T1D patients [57]. Indeed, subjects with T1D (particularly adolescent females) are at greater risk for the development of disordered eating behaviors and eating disorders compared to their peers without diabetes [57].

The most common side effects of semaglutide and tirzepatide observed across the studies examined in this review were the well-established gastrointestinal side effects of these drugs (nausea, vomiting, diarrhea, constipation), which were mostly transient and mild-to-moderate in severity. One severe hypoglycemic episode and severe constipation led to the interruption of tirzepatide in two patients in the study published by Akturk et al. [125]. Furthermore, in the same study, one patient experienced peroneal nerve palsy (foot drop) as a possible consequence of rapid weight loss [125]. In the prospective cohort study published by Navodnik et al. [110], there was only one case of semaglutide withdrawal due to persistent vomiting, while there were no reported cases of severe hypoglycemia or DKA. Moreover, two participants in the randomized crossover trial conducted by Pasqua et al. [105] experienced overt episodes of euglycemic ketosis during semaglutide use, although these episodes did not lead to acidosis and were resolved with adequate changes in carbohydrate intake and insulin therapy. Moreover, the same randomized crossover trial did not report severe hypoglycemic events or DKA during semaglutide use [105]. The remaining studies (retrospective cohort studies) did not observe an increased risk of severe hypoglycemic episodes and DKA deriving from the use of semaglutide and tirzepatide in patients with T1D and LADA.

With regard to other established and putative adverse events associated with the use of incretin analogs [3,143,144,145], the studies examined in this review did not report cases of acute kidney injury, retained gastric contents, aspiration pneumonia, cholecystitis, cholelithiasis, biliary obstruction, ileus, neuropsychiatric events, sarcopenia, leukocytoclastic vasculitis, dermal hypersensitivity reactions, systemic allergic reactions, pancreatitis, or development/worsening of retinopathy.

Although a prospective cohort study [110] and a recently published randomized controlled trial [105] provided robust evidence of clinical benefits deriving from the use of semaglutide in adult T1D patients, it is worth specifying that the other studies examined in the present review are retrospective observational studies, post-hoc analyses, case series and case reports, which evaluated the impact of the off-label use of semaglutide and tirzepatide in patients with T1D and LADA within a real-world context. Moreover, some of these studies were uncontrolled and characterized by a small sample size, short duration of the observation period, possible selection bias of participants, lack of structured protocols for initiating semaglutide or tirzepatide, and lack of a structured drug dose escalation scheme. Lack of data from different ethnic groups (and consequent limited generalizability of the study results to diverse populations), lack of socioeconomic status representation, lack of assessment of circulating C-peptide levels, and use of CGM data obtained from different CGM devices represent additional limitations of many studies reviewed in the present manuscript. Therefore, future large, randomized, placebo-controlled trials on the use of semaglutide and tirzepatide are warranted to definitively clarify the long-term safety and efficacy profile of these medications in patients with T1D (at different stages of the disease and treated with or without AID systems) and in patients with LADA.

### 8.2. Future Research Directions

Given the expected increase in the prevalence of double diabetes associated with overweight/obesity [61], the use of the highly effective weight loss medications semaglutide and tirzepatide (second-generation incretin analogs) in patients with T1D and LADA may keep on rising in the near future. In this regard, a recent pooled cross-sectional analysis using electronic health records from an integrated database including about 257 million US residents across 50 states explored prescribing trends for T1D patients who were prescribed GLP-1 RAs, the dual GIP/GLP-1 RA tirzepatide or SGLT2 inhibitors [146]. This analysis identified 943.456 subjects with T1D from 2010 to 2023, showing that the percentage of T1D patients who were prescribed GLP-1 RAs (dulaglutide, albiglutide, lixisenatide, exenatide, liraglutide, and semaglutide) and the dual GIP/GLP-1 RA tirzepatide increased significantly from 0.3% in 2010 to 6.6% by 2023. Patients who were newly prescribed GLP-1 RAs or tirzepatide had a high prevalence of obesity (69.4%). Within the GLP-1 RA and dual GIP/GLP-1 RA (tirzepatide) subclass, the most pronounced growth regarded semaglutide, whose prescription increased from 0.2% in 2018 to 4.4% in 2023. Tirzepatide, which has been approved for T2D in 2022, reached 1.3% within one year [146].

Therefore, the aforementioned preliminary results highlight the strong need for planning further large, prospective, randomized controlled trials aimed to assess the long-term safety and efficacy of semaglutide and tirzepatide as non-insulin adjunct therapies in both pediatric and adult patients with T1D (at different stages of the disease) and double diabetes, and as anti-hyperglycemic agents in patients with LADA. An ongoing randomized, open-label, phase 3 trial sponsored by the “Centre Hospitalier Universitaire Dijon” (Dijon, France) is already investigating the efficacy of semaglutide (administered as add-on to insulin treatment for 26 weeks) in improving glucose control among adult patients with double diabetes [ClinicalTrials.gov ID: NCT05305794] [147]. Similarly, other randomized clinical trials are currently ongoing to investigate the efficacy of tirzepatide in improving glucose control and reducing body weight in adults with T1D and overweight/obesity (ClinicalTrials.gov ID: NCT06180616) [148], as well as the role of tirzepatide as an adjunct to AID in adults with T1D (ClinicalTrials.gov ID: NCT06630585) [149].

It is therefore clear that many questions regarding the use of semaglutide and tirzepatide in patients with T1D and LADA still remain unanswered. In this regard, future research areas concerning the use of semaglutide and tirzepatide in patients with autoimmune diabetes (including pediatric patients) are the following:

- Investigation of the safety and efficacy of semaglutide and tirzepatide used as non-insulin adjunct therapies in patients with new-onset and long-standing T1D (stages 3 and 4 T1D) for preserving residual beta-cell function, improving glucose control, reducing daily insulin requirements (in terms of both basal and bolus insulin doses), prolonging the honeymoon phase, and preventing or treating excess body weight.
- Investigation of the efficacy of semaglutide and tirzepatide in preventing or halting the progression of chronic (microvascular and macrovascular) complications of diabetes and improving the quality of life in patients with T1D, double diabetes and LADA.
- Investigation of the efficacy of semaglutide and tirzepatide in improving cardiometabolic parameters, treating weight-related conditions (e.g., dyslipidemia, hypertension, metabolic dysfunction-associated steatotic liver disease, metabolic dysfunction-associated steatohepatitis and OSA, among others) and preventing or mitigating exogenous insulin-mediated weight gain among patients with T1D, double diabetes and LADA.
- Investigation of the efficacy of semaglutide and tirzepatide in reducing cardiovascular mortality and all-cause mortality in patients with T1D, double diabetes and LADA.
- Investigation of the safety and efficacy profile of semaglutide and tirzepatide used in pre-symptomatic T1D (stages 1 and 2 T1D) for halting disease progression and preventing or delaying the clinical onset of T1D (stage 3 T1D) through anti-hyperglycemic actions and potential anti-inflammatory, antioxidant, immunomodulatory and beta-cell protective properties (stimulation of beta-cell proliferation and survival).
- Investigation (based on randomized controlled trials) of the use of semaglutide and tirzepatide in patients with T1D and genetic forms of obesity for improvement of glucose control and management of excess body weight.
- Investigation of the use of semaglutide and tirzepatide in patients with T1D, overweight/obesity and PCOS for treating anovulatory menstrual cycles/ irregular menstrual cycles, infertility and/or insulin resistance and/or excess body weight and/or hyperandrogenism and/or weight-related comorbidities in this population [150,151].
- Investigation of the safety and efficacy profile of semaglutide and tirzepatide used in patients with T1D who have previously undergone allogeneic islet transplantation/pancreas transplantation and/or kidney transplantation for improving glucose control, reducing beta-cell glucotoxicity, mitigating the immunosuppressant-induced beta-cell dysfunction [152], preserving endogenous insulin secretion, preventing graft dysfunction and graft failure, promoting graft function and survival, and possibly reducing the required doses of immunosuppressants through anti-hyperglycemic actions and potential anti-inflammatory, antioxidant, immunomodulatory and beta-cell protective properties (stimulation of beta-cell proliferation and survival). According to these hypotheses, experimental evidence from in vitro models of autoimmune diabetes suggested that liraglutide can preserve insulin secretion by exerting anti-inflammatory actions that protect beta cells from the autoimmune attack [153]. Interestingly, liraglutide has also been shown to enhance the efficacy of mesenchymal stem cells in preserving islet beta-cell function in non-obese diabetic (NOD) mice [154], which represent a well-established animal model of human T1D [155]. More recently, a study conducted in mice receiving islet or cardiac allotransplantation documented that GLP-1 receptor acts as a T cell-negative co-stimulatory molecule, and GLP-1 receptor signaling mitigates alloimmune response, reduces T lymphocyte graft infiltration, and prolongs allograft survival [156].
- Investigation of the safety and efficacy of semaglutide and tirzepatide as adjuvant therapeutic agents able to reduce glucotoxicity and promote successful clinical outcomes of investigational beta-cell replacement strategies based on the use of encapsulated stem cell-derived insulin-producing beta cells in patients with T1D.
- Investigation of the safety and efficacy profile of semaglutide and tirzepatide used in patients with LADA for preserving beta-cell function, reducing insulin resistance, improving glucose control and delaying or preventing the need for insulin therapy.
- Investigation of the safety and efficacy profile of semaglutide and tirzepatide used in patients with other forms of autoimmune diabetes (e.g., checkpoint inhibitor-associated autoimmune diabetes mellitus) [157] for preserving beta-cell function and improving glucose control.
- Investigation of the impact of semaglutide and tirzepatide on lean body mass (through diagnostic tools such as the dual-energy X-ray absorptiometry and/or the bioelectrical impedance analysis) in patients with T1D, double diabetes and LADA. Indeed, incretin analog therapy has been shown to cause a variable lean mass loss across studies (besides a substantial fat mass reduction) [158,159]. Since sarcopenia is strongly associated with morbidity and mortality [160], there is a great need for investigation of nutrition and exercise strategies as well as novel pharmacotherapies aimed to preserve muscle mass and function during incretin analog therapy [158,161].
- Investigation of the safety and efficacy profile of combination therapies based on the co-administration of semaglutide (or tirzepatide) and amylin analogs in patients with T1D, double diabetes and LADA.

With regard to the latter point, combinations therapies based on the co-administration of semaglutide (or tirzepatide) and amylin analogs may offer, as compared to GLP-1 RA or dual GIP/GLP-1 RA monotherapies, greater glycemic improvements and weight loss reductions in patients with autoimmune diabetes and comorbid overweight/obesity. Amylin (a.k.a. islet amyloid polypeptide or IAPP) is a peptide hormone which is co-secreted with insulin by pancreatic beta cells in response to food intake, at a level of approximately 1% that of insulin [162]. Amylin plays a relevant role in glucose homeostasis by slowing gastric emptying, promoting satiety (through action on selected areas of the central nervous system) and suppressing postprandial glucagon secretion, thus preventing excessive caloric intake and reducing postprandial glucose excursions [163,164,165,166,167].

T1D is also regarded as a two-hormone deficiency disorder [166], given that T1D-related autoimmune destruction of beta cells leads to amylin deficiency (in addition to insulin deficiency) [163,164,168]. Coupled with insulin deficiency and defective suppression of alpha-cell glucagon secretion in the postprandial period (due to the loss of paracrine inhibition by insulin secreted from the neighboring beta cells) [169,170], amylin deficiency represents an additional cause of poor postprandial glucose control, postprandial hyperglucagonemia and increased glycemic variability in patients with T1D [168].

To date, pramlintide is the only commercially available amylin analog (administered subcutaneously at mealtime) approved by the FDA as an adjunctive treatment to insulin therapy in patients with T1D and T2D [171,172,173]. As we previously mentioned, Wong et al. [121] suggested the possible existence of synergistic weight-loss and glucose-lowering effects of semaglutide and pramlintide in two adults with T1D and comorbid obesity (without reporting significant side effects during this combination therapy). Accordingly, a study conducted in rats demonstrated that the administration of an amylin analog together with the GLP-1 RA liraglutide determined synergistic anorectic and weight-loss effects [174]. Thus, it would be interesting to assess whether novel once-weekly subcutaneous long-acting amylin analogs (like cagrilintide) or novel co-formulations containing long-acting amylin analogs and long-acting GLP-1 RA (like CagriSema, which contains cagrilintide and semaglutide) [175] can exert a therapeutic role in patients with T1D, double diabetes and LADA. Significant therapeutic benefits of such combination therapies may partly depend on greater suppression of postprandial glucagon secretion as compared to the single administration of GLP-1 RAs, tirzepatide (dual GIP/GLP-1 RA) or amylin analogs. However, it will also be critical to ascertain whether co-administration of amylin analogs and GLP-1 RAs is associated with higher rates of gastrointestinal side effects or other adverse effects like hypoglycemic episodes. Indeed, it is worth noting that T1D is also characterized by an impaired glucagon secretion in responses to hypoglycemia [176], apart from exaggerated postprandial glucagon secretion [170,177].

As we previously mentioned, over the last years there has been a progressive increase in the proportion of T1D patients affected by overweight/obesity and/or insulin resistance [45,61]. Mechanisms accounting for development of overweight/obesity in T1D patients include: iatrogenic peripheral hyperinsulinemia related to intensive insulin therapy (due to peripheral insulin administration, such as subcutaneous insulin administration) [178], physical inactivity and increased caloric intake [45] (for example, due to the fear of hypoglycemia or due to the treatment of frequent episodes of hypoglycemia) [44,179], unhealthy food consumption, T1D-specific biopsychosocial burden, exposure to an obesogenic environment, socioeconomic and cultural factors [45], binge eating disorder and disordered eating behaviors [180], genetic susceptibility, epigenetics, hormonal alterations other than insulin deficiency (e.g., alterations involving the secretion of amylin, glucagon, leptin, ghrelin and other gastrointestinal hormones), dysregulation of biologic mechanisms regulating appetite and satiety, sociodemographic disparities, gut microbiota dysbiosis, adipose tissue dysfunction, lipotoxicity, and glucotoxicity [45,181].

**Figure 2:**
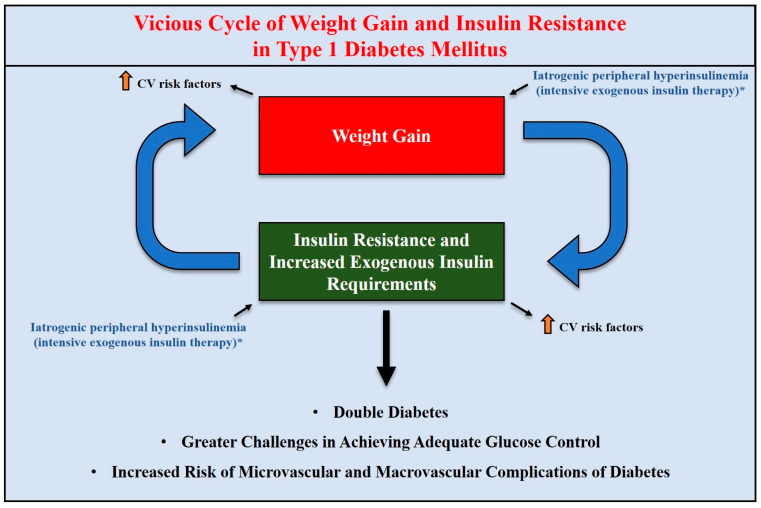
**Vicious cycle of weight gain, insulin resistance and increased exogenous insulin requirements in patients with T1D**. In patients with T1D, this perpetuating cycle leads to double diabetes, which is characterized by the coexistence of clinical features of both T1D and T2D in the same subject. Iatrogenic peripheral hyperinsulinemia during intensive exogenous insulin therapy promotes insulin resistance and weight gain. In turn, excess weight gain promotes insulin resistance, which leads to higher exogenous insulin requirements. Moreover, insulin resistance and excess weight gain due to intensive exogenous insulin therapy can increase cardiovascular risk factors (e.g., hypertension, dyslipidemia). *Iatrogenic peripheral hyperinsulinemia during intensive exogenous insulin therapy is related to subcutaneously administered exogenous insulin, which bypasses first-pass hepatic insulin extraction. Abbreviations: CV, cardiovascular; T1D, type 1 diabetes; T2D, type 2 diabetes.

Moreover, T1D patients can develop insulin resistance due to several mechanisms, including iatrogenic peripheral hyperinsulinemia (related to intensive insulin therapy based on subcutaneously administered exogenous insulin, which bypasses first-pass hepatic insulin extraction) [182], genetic factors, glucotoxicity, lipotoxicity, excessive accumulation of adipose tissue (particularly visceral adipose tissue), adipose tissue dysfunction (overweight/obesity), chronic systemic low-grade inflammation, oxidative stress, insulin-like growth factor-1 (IGF-1) deficiency and growth hormone (GH) hypersecretion, among others [50,183,184]. This creates a vicious cycle where iatrogenic hyperinsulinemia (related to intensive insulin therapy based on subcutaneously administered exogenous insulin) promotes insulin resistance and weight gain [182,185,186,187]. In turn, excess weight gain promotes insulin resistance [187,188,189,190,191], which leads to higher exogenous insulin requirements. Moreover, insulin resistance and excess weight gain due to intensive insulin therapy can increase cardiovascular risk factors (e.g., hypertension, dyslipidemia) [66,67,187,191,192]. Thus, strategies aimed to break the perpetuating cycle of weight gain, insulin resistance and increased exogenous insulin requirements (Figure 2) are highly desirable in patients with double diabetes, where clinical features of both T1D and T2D coexist in the same subject [61].

Semaglutide and tirzepatide can effectively break this perpetuating dangerous cycle. Indeed, the use of semaglutide and tirzepatide in T1D and LADA across most studies reviewed in this manuscript has been associated with a substantial reduction of daily TDD of insulin (or even with the interruption of insulin therapy), along with remarkable weight loss effects. The weight loss and glucose-lowering effects of semaglutide and tirzepatide in patients with autoimmune diabetes appear to be partly mediated by the insulin-sensitizing actions of these drugs. In fact, it has been shown that semaglutide and tirzepatide reduce insulin resistance in patients with T2D, an effect that appears to be primarily mediated by the substantial weight loss effects of these drugs [81,109,193,194,195]. However, a preclinical study conducted in high-fat-diet-fed obese IR mice documented that tirzepatide enhances insulin action to a greater extent than semaglutide, since it exerts both weight-dependent and weight-independent (through GIP receptor agonism) insulin-sensitizing actions [196]. Mechanistically, tirzepatide has been shown to improve insulin sensitivity by enhancing insulin-stimulated glucose disposal in skeletal muscle and white adipose tissue, and by inducing the expression of genes linked to the oxidation of glucose, lipids and branched-chain amino acids in brown adipose tissue, thus reducing excess nutrient delivery to metabolically relevant organs and consequently enhancing systemic insulin sensitivity [196].

Another interesting research area is the investigation of potential cardiorenal benefits of semaglutide and tirzepatide in patients with T1D, double diabetes and LADA.

In particular, increased risk of cardiovascular disease and chronic kidney disease has been reported in patients with T1D [197,198]. Therefore, there is an unmet need for cardioprotective and nephroprotective adjuncts able to increase life expectancy and improve quality of life of T1D patients [199]. This aspect is highly relevant, as cardiovascular disease and diabetic kidney disease are leading causes of death in T1D patients [197,200,201]. Even though such conditions may be attributable to traditional cardiorenal risk factors, non-traditional risk factors (such as insulin resistance) may also play a role in their pathophysiology. In fact, insulin resistance in T1D has been linked to coronary atherosclerosis [67], cardiovascular disease, diabetic nephropathy and all-cause mortality [66,202], and it is partly driven by subcutaneously administered exogenous insulin itself [182]. Semaglutide and tirzepatide are undoubtedly promising drug candidates to reduce the cardiorenal risk and the development of chronic microvascular and macrovascular complications of diabetes in patients with T1D. Preliminary clinical evidence in this direction has already been provided by the prospective study conducted by Navodnik et al. [110], who showed that semaglutide (as add-on treatment to insulin) positively impacted endothelial function in patients with long-standing T1D. Additionally, a preclinical study conducted by Yan et al. [203] on C57BL/6J mice with streptozotocin-induced diabetes mellitus found that 8-week administration of semaglutide (injected subcutaneously at a weekly dose of 0.15 mg/kg) decreased myocardial oxidative stress by activating the Sirt1/AMPK (AMP-activated protein kinase) pathway, thus reducing cardiomyocyte apoptosis, restoring the expression of connexin 43 (a.k.a. Cx43, the predominant protein forming gap junctions and non-junctional hemichannels in ventricular myocardium) [204], and attenuating pathological electrophysiological remodeling and cardiac dysfunction in the context of diabetic cardiomyopathy [203]. More importantly, Frampton et al. [205] recently published the study protocol of a phase 2, double-blind, randomized, placebo-controlled trial termed RESET1 (“REducing cardiometabolic risk with SEmaglutide in Type 1 diabetes”). This trial will recruit 60 eligible adults with T1D at St Vincent’s Hospital (a teaching hospital in Sydney, Australia) to assess the impact of 26-week administration of once-weekly subcutaneous semaglutide (at a weekly dose of up to 1.0 mg) on carotid-femoral pulse wave velocity (cfPWV) [205], which serves as a measure of arterial stiffness and as a surrogate marker of cardiorenal risk in T1D [206]. Inclusion criteria will be the following: age ranging from 25 to 70 years; diabetes duration equal to or greater than 2 years; BMI ≥ 25 kg/m^2^; HbA1c ≥ 7%; and presence of at least one cardiovascular risk factor (microalbuminuria, and/or hypertension, and/or use of anti-hypertensive drugs, and/or hyperlipidemia, and/or use of lipid-lowering drugs, and/or current smoking) [205].

Another key point to be addressed in future clinical trials is the establishment of the most proper doses of novel incretin analogs required to achieve the desired clinical outcomes in different subgroups of patients with autoimmune diabetes (e.g., patients with new-onset T1D, patients with long-standing T1D, patients with double diabetes and overweight/obesity, patients with LADA). Indeed, the proper doses of incretin analogs may vary depending on the specific therapeutic goals. For example, low semaglutide or tirzepatide doses may be sufficient for safe and effective preservation of residual beta-cell function in patients with new-onset T1D (who usually exhibit normal or low BMI values), as it has been demonstrated by Dandona et al. (who used a maximum weekly semaglutide dose of 0.5 mg) [112], whereas higher semaglutide or tirzepatide doses are likely necessary to achieve substantial weight loss and adequate glucose control in patients with double diabetes. These considerations also apply to patients with distinct T1D endotypes and to patients with heterogeneous clinical manifestations of LADA. The investigation of cost-effectiveness of different doses of novel incretin analogs based on the desired clinical outcomes has relevant implications, particularly in light of the recent widespread shortage of these drugs (due to the transient fragility of global pharmaceutical supply chain) and frequent issues regarding the high costs and insurance coverage of these medications [207,208,209].

### 8.3. Practical Considerations Regarding the Use of Semaglutide and Tirzepatide in Patients with T1D, Double Diabetes and LADA

Even though semaglutide and tirzepatide are currently not approved for the treatment of patients with autoimmune diabetes, practical considerations regarding the use of these medications should be taken into account in view of their increasing off-label use in this population within real-world contexts [146]. Indeed, healthcare providers tend to prescribe these medications off-label, particularly for patients with T1D facing overweight/obesity and/or insulin resistance.

One of the main aspects to consider when starting semaglutide or tirzepatide therapy in patients with autoimmune diabetes is the concomitant need for insulin dose adjustments, particularly because there is still no evidence-based insulin titration guidance for semaglutide and tirzepatide use in T1D patients. First, patients should be properly informed about the potential adverse effects of these medications. In particular, patients with autoimmune diabetes should be instructed on how to recognize and manage the most dreaded side effects of semaglutide and tirzepatide in this population, such as severe hypoglycemia, DKA and euglycemic diabetic ketoacidosis (euDKA). Second, patients should be properly informed about the expected reductions in insulin requirements after initiation and dose escalation of semaglutide or tirzepatide. Proper insulin dose adjustments during semaglutide or tirzepatide therapy are indeed essential to minimize the potential risks of semaglutide- or tirzepatide-related adverse events. While significant insulin dose reductions after the initiation of semaglutide or tirzepatide therapy are generally required to avoid hypoglycemia, excessive insulin dose reductions can lead to DKA or euDKA. Therefore, insulin dose adjustments should always be made on the basis of clinical judgment and clinical decision making by expert diabetologists. Third, patients should receive proper education by healthcare providers on how to recognize possible signs and symptoms of hypoglycemia and DKA/euDKA [210,211,212], and on when they should measure blood ketone values through the use of ketone meters.

Useful suggestions regarding insulin titration during semaglutide or tirzepatide therapy come from some of the studies discussed in this review. Remarkably, the use of second-generation insulin analogs (semaglutide and tirzepatide) may serve as a valid adjunct to closed-loop insulin therapy by preventing or treating excess body weight and by enhancing the glycemic and metabolic benefits of AID systems in patients with T1D and comorbid overweight/obesity. In this regard, the retrospective study by Karakus et al. [127] demonstrated that tirzepatide, in addition to AID, led to significant improvement of glucose control, reduction of TDD of insulin, and weight loss among adult patients with T1D and obesity. Based on their findings, Karakus et al. [127] suggest reducing TDD of insulin by 25% during tirzepatide initiation in adults with T1D who use AID systems and have a baseline HbA1c lower than 7.5%. Specifically, the authors suggest reducing basal and bolus insulin doses by 20–30% in line with the weight loss observed within the first month of tirzepatide initiation (administered at a starting weekly dose of 2.5 mg). During the following months, an additional 5–10% reduction in TDD of insulin (as compared to baseline) may be required. The authors underline that such reductions can be achieved through distinct approaches among patients using AID, CSII or MDI insulin therapy. With regard to AID systems, they suggest adjusting the modifiable settings (e.g., insulin to carbohydrate ratio, correction factor, active insulin time, target glucose) based on the functionality of the AID systems with intention to reduce insulin delivery by 20–30% during tirzepatide therapy initiation. On the other hand, patients on MDI insulin therapy may be more susceptible to hypoglycemia than AID users due to the lack of basal automation and may therefore require more frequent evaluations and adjustments of their insulin doses. In the latter category of patients, 15% basal insulin reduction and 20–25% bolus insulin reduction during the first months can be implemented with monitoring nighttime and postprandial glucose values for fine-tuning the basal and bolus insulin doses, respectively. The authors suggest that patients using non-AID insulin pumps may reduce basal insulin rates for all hours, while taking into account diurnal rhythm and changing basal insulin requirements throughout the day. In addition, the authors suggest that insulin dose reductions may depend on baseline HbA1c values. For example, T1D patients with higher HbA1c values (>8%) may not require a 20–30% reduction in TDD of insulin, whereas T1D patients with HbA1c values close to 7% (indicating more optimal insulin use) may require close to 30% reduction in TDD of insulin. Given the baseline HbA1c value of 7.0% observed in their study cohort, the authors assumed that up to 15% reduction in TDD of insulin may be a reasonable starting strategy for patients with baseline HbA1c values >8%, while patients with baseline HbA1c values >9% probably may not require any insulin dose reduction (particularly during the first month of adjunctive GLP-1 RA therapy) [127].

An international group of experts in diabetes treatment recently published a consensus report on the use of GLP-1 RAs or novel dual GIP/GLP-1 RA tirzepatide as an adjunctive therapy to AID systems in adults with T1D [213]. According to the consensus opinions of the participants, the use of incretin analogs would be particularly useful in adults with T1D using AID systems, as these medications have the potential to improve glycemic and metabolic outcomes in this population. Moreover, the consensus report highlights that the beneficial effects of these drugs are likely not outweighed by the risk of severe hypoglycemia or DKA, given the current availability of advanced AID systems able to effectively predict and prevent such adverse events through algorithm-based changes in insulin delivery [213]. Moreover, the consensus group felt that if GLP-1 RAs and the novel dual GIP/GLP-1 RA tirzepatide show efficacy in the aforementioned subset of T1D patients, then it will be more likely that these drugs can eventually be widely approved for all T1D patients without posing a significant risk of hypoglycemia or DKA, especially in light of the current widespread use of CGM systems in this population and in view of the possible availability of continuous ketone monitoring (CKM) systems in the near future [213,214].

**Figure 3:**
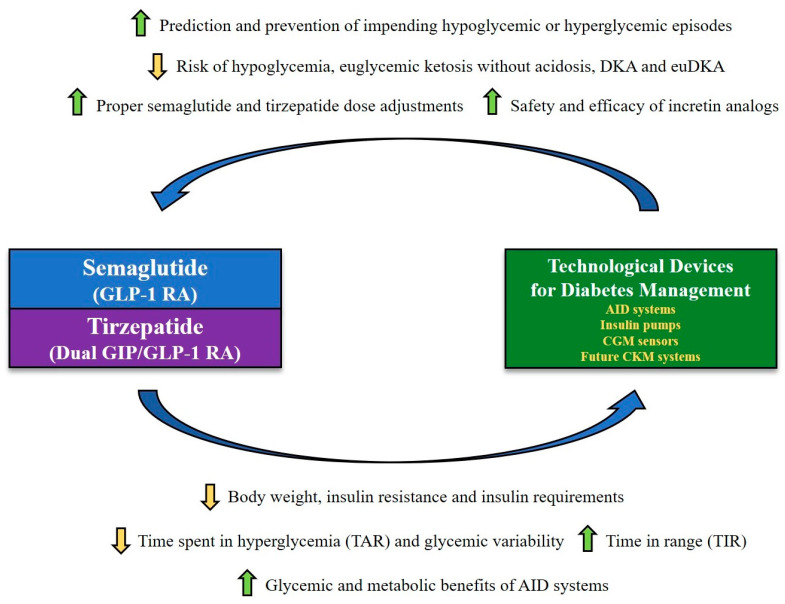
**Potential synergistic benefits of second-generation incretin analogs (semaglutide and tirzepatide) and advanced technological devices used for diabetes management in patients with T1D, double diabetes and LADA.** Second-generation incretin analogs semaglutide and tirzepatide may be valid allies of the current advanced technological devices employed for the management of autoimmune diabetes (particularly AID systems) by preventing or reducing excess body weight, increasing the time spent in recommended target blood glucose range, reducing time spent in hyperglycemia, and decreasing glycemic variability, insulin resistance and insulin requirements, thus enhancing the glycemic and metabolic benefits of diabetes technological devices/AID systems. On the other hand, the currently available advanced technological devices used for the management of autoimmune diabetes (particularly AID systems), which can predict impending episodes of hypoglycemia or hyperglycemia and promptly and dynamically adjust insulin delivery, may help prevent the most dreaded adverse effects of incretin analogs in patients with autoimmune diabetes (especially hypoglycemia, DKA and euDKA). Abbreviations: AID, automated insulin delivery; CGM, continuous glucose monitoring; CKM, continuous ketone monitoring; DKA, diabetic ketoacidosis; euDKA, euglycemic diabetic ketoacidosis; GIP, glucose-dependent insulinotropic polypeptide; GLP-1, glucagon-like peptide-1; RA, receptor agonist; TAR, time above range; TIR, time in range; T1D, type 1 diabetes.

In light of the current literature, it can be postulated that the second-generation incretin analogs semaglutide and tirzepatide may be valid allies of the advanced technologies employed for the management of T1D, particularly by preventing or treating excess body weight, reducing insulin resistance and insulin requirements, and enhancing the glycemic and metabolic benefits of AID systems. On the other hand, the currently available advanced AID systems (able to predict impending episodes of hypoglycemia or hyperglycemia and to promptly and dynamically adjust insulin delivery), together with future CKM systems, may help effectively prevent the most dreaded adverse effects of incretin analogs in T1D patients, namely hypoglycemia, DKA and euDKA. Figure 3 illustrates the potential synergistic benefits of second-generation incretin analogs (semaglutide and tirzepatide) and advanced technological devices used for diabetes management in patients with T1D, double diabetes and LADA.

Large prospective studies are certainly needed to establish structured and evidence-based insulin titration protocols (regarding both basal and bolus insulin doses) to avoid hypoglycemia, ketosis and DKA/euDKA in T1D patients using semaglutide and tirzepatide in addition to insulin therapy (administered either via insulin pens or via insulin pumps and AID systems) and exhibiting different degrees of glucose control, excess body weight and/or insulin resistance.

At the same time, clinicians should always verify the proper functioning of CGM sensors and insulin pumps, given that CGM sensor and/or insulin pump malfunctions can precipitate the occurrence of adverse events during semaglutide or tirzepatide therapy, as in the case described by Sharma et al. [129]. Moreover, clinicians should be aware of the existence of specific conditions that can precipitate or exacerbate the common gastrointestinal side effects of incretin analogs, such as the isomaltase deficiency identified in the case described by Seetharaman and Cengiz [139].

Gradual semaglutide and tirzepatide dose titration (as per clinical guidelines) helps prevent or minimize the severity of the most common side effects of GLP1- RA (nausea, vomiting, diarrhea, constipation). Additionally, adoption of specific behavioral and nutritional strategies is also crucial to minimize the occurrence and/or the severity of the most common gastrointestinal side effects of GLP-1 RA, as it has recently been published by an expert panel including endocrinologists, nephrologists, primary care physicians, internists, cardiologists and diabetes nurse educators [215].

With regard to the strategies useful for prevention of possible muscle loss related to the use of second-generation incretin analogs (semaglutide and tirzepatide), preliminary evidence suggests that individuals taking these medications should aim for a daily protein intake ≥1.2 g/kg actual body weight and for physical activity programs including resistance training exercises (one to three sets of 8–12 repetitions for each major muscle group) at least twice a week [216]. However, it is important to highlight that ideal daily protein intake is highly variable across individuals and depends on several factors, such as age, gender, caloric intake, body weight, fitness level, physical activity (type, intensity and frequency of physical activity), presence of chronic diseases and overall health. For example, daily protein requirements may be higher for subjects who engage in regular physical activity, whereas daily protein intake should be restricted in patients with chronic kidney disease. Moreover, nutrient timing strategies that maximize daily protein intake (e.g., frequent consumption of small portions of high-protein foods evenly distributed throughout the day) and consumption of high-quality protein sources rich in essential amino acids (such as animal and dairy-based proteins or high-quality plant-based proteins like soy) may also provide significant benefits in terms of preservation of skeletal muscle health [216].

Therefore, future large, randomized, placebo-controlled trials on the use of semaglutide and tirzepatide are warranted to definitively clarify the long-term safety and efficacy profile of these medications in patients with T1D (at different stages of the disease and treated with or without AID systems), double diabetes and LADA. Future studies will also be required to ascertain whether pre-existing diabetic retinopathy (at different stages) represents a contraindication to the use of semaglutide or tirzepatide in patients with autoimmune diabetes.

Meanwhile, the off-label use of semaglutide and tirzepatide should be avoided in T1D patients at increased risk for gastroparesis (such as those with diabetic gastrointestinal autonomic neuropathy) [217], in patients with (or at risk for) restrictive eating behaviors (as it has been suggested by the case published by Seetharaman and Cengiz) [139], and in patients with “diabulimia”, a term indicating an eating disorder experienced by T1D patients and characterized by the deliberate administration of insufficient insulin to maintain glucose control for the purpose of weight loss [218,219].

## 9. Conclusions

In patients with long-standing T1D and double diabetes, the use of novel incretin analogs (semaglutide and tirzepatide) has been associated with remarkable clinical benefits, which mostly consist of clinically meaningful improvement of glucose control and substantial weight loss. Semaglutide therapy has also been associated with improved glucose control, reduced daily insulin requirements, prolonged honeymoon phase and preserved residual beta-cell function in patients with new-onset T1D. Moreover, both semaglutide and tirzepatide may potentially improve glucose control, preserve beta-cell function and reduce insulin resistance in patients with LADA, even though there is a paucity of studies conducted in this population.

Although a prospective cohort study and a recently published randomized controlled trial have demonstrated that semaglutide leads to significant improvements in glucose control and substantial weight loss in adults with T1D, the remaining evidence for the efficacy of semaglutide and tirzepatide in patients with autoimmune diabetes mostly comes from retrospective studies, case series and case reports. In addition, future prospective studies will need to establish whether semaglutide and tirzepatide provide significant cardiorenal benefits without increasing the risk of severe hypoglycemia, DKA and euDKA in patients with autoimmune diabetes who use different methods of insulin delivery (MDI insulin therapy, insulin pumps, AID systems). These studies will also help clarify the cost-effectiveness of semaglutide and tirzepatide for the treatment of autoimmune diabetes. The latter aspect is highly relevant, given the high costs of these medications. In conclusion, future large, randomized, placebo-controlled trials are needed to confirm the long-term safety and efficacy profile of semaglutide and tirzepatide used for different purposes in patients with T1D (at different stages of the disease), double diabetes and LADA.

## Data Availability

Not applicable.

## Supplementary Materials

The following supporting information can be downloaded at https://www.mdpi.com/article/10.3390/jcm14041303/s1. Table S1. Clinical studies investigating the use of semaglutide in patients with autoimmune diabetes; Table S2. Clinical studies investigating the use of tirzepatide in patients with autoimmune diabetes.

## References

[1] Drucker D.J., Holst J.J. (2023). The expanding incretin universe: From basic biology to clinical translation. Diabetologia.

[2] Kim W., Egan J.M. (2008). The role of incretins in glucose homeostasis and diabetes treatment. Pharmacol. Rev..

[3] Drucker D.J. (2024). Efficacy and Safety of GLP-1 Medicines for Type 2 Diabetes and Obesity. Diabetes Care.

[4] Le R., Nguyen M.T., Allahwala M.A., Psaltis J.P., Marathe C.S., Marathe J.A., Psaltis P.J. (2024). Cardiovascular Protective Properties of GLP-1 Receptor Agonists: More than Just Diabetic and Weight Loss Drugs. J. Clin. Med..

[5] Perkovic V., Tuttle K.R., Rossing P., Mahaffey K.W., Mann J.F.E., Bakris G., Baeres F.M.M., Idorn T., Bosch-Traberg H., Lausvig N.L. (2024). Effects of Semaglutide on Chronic Kidney Disease in Patients with Type 2 Diabetes. N. Engl. J. Med..

[6] Apperloo E.M., Gorriz J.L., Soler M.J., Cigarrán Guldris S., Cruzado J.M., Puchades M.J., López-Martínez M., Waanders F., Laverman G.D., van der Aart-van der Beek A. (2024). Semaglutide in patients with overweight or obesity and chronic kidney disease without diabetes: A randomized double-blind placebo-controlled clinical trial. Nat. Med..

[7] Lincoff A.M., Brown-Frandsen K., Colhoun H.M., Deanfield J., Emerson S.S., Esbjerg S., Hardt-Lindberg S., Hovingh G.K., Kahn S.E., Kushner R.F. (2023). Semaglutide and Cardiovascular Outcomes in Obesity without Diabetes. N. Engl. J. Med..

[8] Marso S.P., Bain S.C., Consoli A., Eliaschewitz F.G., Jódar E., Leiter L.A., Lingvay I., Rosenstock J., Seufert J., Warren M.L. (2016). Semaglutide and Cardiovascular Outcomes in Patients with Type 2 Diabetes. N. Engl. J. Med..

[9] Bray J.J.H., Foster-Davies H., Salem A., Hoole A.L., Obaid D.R., Halcox J.P.J., Stephens J.W. (2021). Glucagon-like peptide-1 receptor agonists improve biomarkers of inflammation and oxidative stress: A systematic review and meta-analysis of randomised controlled trials. Diabetes Obes. Metab..

[10] Wong C.K., McLean B.A., Baggio L.L., Koehler J.A., Hammoud R., Rittig N., Yabut J.M., Seeley R.J., Brown T.J., Drucker D.J. (2024). Central glucagon-like peptide 1 receptor activation inhibits Toll-like receptor agonist-induced inflammation. Cell Metab..

[11] Hammoud R., Kaur K.D., Koehler J.A., Baggio L.L., Wong C.K., Advani K.E., Yusta B., Efimova I., Gribble F.M., Reimann F. (2024). Glucose-dependent insulinotropic polypeptide receptor signaling alleviates gut inflammation in mice. JCI Insight.

[12] Xia Y., Jin J., Sun Y., Kong X., Shen Z., Yan R., Huang R., Liu X., Xia W., Ma J. (2024). Tirzepatide’s role in targeting adipose tissue macrophages to reduce obesity-related inflammation and improve insulin resistance. Int. Immunopharmacol..

[13] Toki S., Abney M., Zhang J., Rusznak M., Warren C.M., Newcomb D.C., Cahill K.N., Drucker D.J., Niswender K.D., Peebles R.S. (2024). Endogenous Glucagon-Like Peptide-1 Receptor and Glucose-Dependent Insulinotropic Polypeptide Receptor Signaling Inhibits Aeroallergen-Induced Innate Airway Inflammation. Allergy.

[14] Atkinson M.A., Eisenbarth G.S., Michels A.W. (2014). Type 1 diabetes. Lancet.

[15] Holt R.I.G., DeVries J.H., Hess-Fischl A., Hirsch I.B., Kirkman M.S., Klupa T., Ludwig B., Nørgaard K., Pettus J., Renard E. (2021). The management of type 1 diabetes in adults. A consensus report by the American Diabetes Association (ADA) and the European Association for the Study of Diabetes (EASD). Diabetologia.

[16] Syed F.Z. (2022). Type 1 Diabetes Mellitus. Ann. Intern. Med..

[17] Vehik K., Dabelea D. (2011). The changing epidemiology of type 1 diabetes: Why is it going through the roof?. Diabetes Metab. Res. Rev..

[18] Patterson C.C., Harjutsalo V., Rosenbauer J., Neu A., Cinek O., Skrivarhaug T., Rami-Merhar B., Soltesz G., Svensson J., Parslow R.C. (2019). Trends and cyclical variation in the incidence of childhood type 1 diabetes in 26 European centres in the 25 year period 1989–2013: A multicentre prospective registration study. Diabetologia.

[19] Rewers M., Ludvigsson J. (2016). Environmental risk factors for type 1 diabetes. Lancet.

[20] Paschou S.A., Papadopoulou-Marketou N., Chrousos G.P., Kanaka-Gantenbein C. (2018). On type 1 diabetes mellitus pathogenesis. Endocr. Connect..

[21] Greenbaum C.J., Speake C., Krischer J., Buckner J., Gottlieb P.A., Schatz D.A., Herold K.C., Atkinson M.A. (2018). Strength in Numbers: Opportunities for Enhancing the Development of Effective Treatments for Type 1 Diabetes-The TrialNet Experience. Diabetes.

[22] Abdul-Rasoul M., Habib H., Al-Khouly M. (2006). ‘The honeymoon phase’ in children with type 1 diabetes mellitus: Frequency, duration, and influential factors. Pediatr. Diabetes.

[23] Fonolleda M., Murillo M., Vázquez F., Bel J., Vives-Pi M. (2017). Remission Phase in Paediatric Type 1 Diabetes: New Understanding and Emerging Biomarkers. Horm. Res. Paediatr..

[24] Aly H., Gottlieb P. (2009). The honeymoon phase: Intersection of metabolism and immunology. Curr. Opin. Endocrinol. Diabetes Obes..

[25] Oram R.A., Jones A.G., Besser R.E., Knight B.A., Shields B.M., Brown R.J., Hattersley A.T., McDonald T.J. (2014). The majority of patients with long-duration type 1 diabetes are insulin microsecretors and have functioning beta cells. Diabetologia.

[26] Wang L., Lovejoy N.F., Faustman D.L. (2012). Persistence of prolonged C-peptide production in type 1 diabetes as measured with an ultrasensitive C-peptide assay. Diabetes Care.

[27] Keenan H.A., Sun J.K., Levine J., Doria A., Aiello L.P., Eisenbarth G., Bonner-Weir S., King G.L. (2010). Residual insulin production and pancreatic ß-cell turnover after 50 years of diabetes: Joslin Medalist Study. Diabetes.

[28] Alan M.S., Tayebi A., Afshar E.J., Alan S.S., Alan M.S., Fazeli R., Sohbatzade T., Samimisedeh P., Rastad H. (2024). Association of detectable C-peptide levels with glycemic control and chronic complications in individuals with type 1 diabetes mellitus: A systematic review and meta-analysis. J. Diabetes Its Complicat..

[29] Steffes M.W., Sibley S., Jackson M., Thomas W. (2003). Beta-cell function and the development of diabetes-related complications in the diabetes control and complications trial. Diabetes Care.

[30] Gibb F.W., McKnight J.A., Clarke C., Strachan M.W.J. (2020). Preserved C-peptide secretion is associated with fewer low-glucose events and lower glucose variability on flash glucose monitoring in adults with type 1 diabetes. Diabetologia.

[31] Abuabat M.I., Budhram D.R., Bapat P., Bakhsh A.M., Verhoeff N., Mumford D., Orszag A., Fralick M., Weisman A., Lovblom L.E. (2024). 1440-P: C-Peptide Preservation and Diabetic Ketoacidosis (DKA) Risk in Type 1 Diabetes. Diabetes.

[32] Pinheiro M.M., Pinheiro F.M.M., Garo M.L., Pastore D., Pacifici F., Ricordi C., Della-Morte D., Infante M. (2024). Prevention and treatment of type 1 diabetes: In search of the ideal combination therapy targeting multiple immunometabolic pathways. Metab. Target Organ Damage.

[33] Infante M., Ricordi C. (2019). Editorial—Moving forward on the pathway of targeted immunotherapies for type 1 diabetes: The importance of disease heterogeneity. Eur. Rev. Med. Pharmacol. Sci..

[34] Infante M., Alejandro R., Fabbri A., Ricordi C. (2022). The heterogeneity of type 1 diabetes: From immunopathology to immune intervention. Translational Autoimmunity.

[35] Battaglia M., Ahmed S., Anderson M.S., Atkinson M.A., Becker D., Bingley P.J., Bosi E., Brusko T.M., DiMeglio L.A., Evans-Molina C. (2020). Introducing the Endotype Concept to Address the Challenge of Disease Heterogeneity in Type 1 Diabetes. Diabetes Care.

[36] Gregory G.A., Robinson T.I.G., Linklater S.E., Wang F., Colagiuri S., de Beaufort C., Donaghue K.C., Magliano D.J., Maniam J., Orchard T.J. (2022). Global incidence, prevalence, and mortality of type 1 diabetes in 2021 with projection to 2040: A modelling study. Lancet Diabetes Endocrinol..

[37] Cudini A., Fierabracci A. (2023). Advances in Immunotherapeutic Approaches to Type 1 Diabetes. Int. J. Mol. Sci..

[38] Keam S.J. (2023). Teplizumab: First Approval. Drugs.

[39] Pinheiro M.M., Pinheiro F.M.M., Diniz S.N., Fabbri A., Infante M. (2021). Combination of vitamin D and dipeptidyl peptidase-4 inhibitors (VIDPP-4i) as an immunomodulation therapy for autoimmune diabetes. Int. Immunopharmacol..

[40] Keymeulen B., De Groot K., Jacobs-Tulleneers-Thevissen D., Thompson D.M., Bellin M.D., Kroon E.J., Daniels M., Wang R., Jaiman M., Kieffer T.J. (2024). Encapsulated stem cell-derived β cells exert glucose control in patients with type 1 diabetes. Nat. Biotechnol..

[41] Neumann M., Arnould T., Su B.L. (2023). Encapsulation of stem-cell derived β-cells: A promising approach for the treatment for type 1 diabetes mellitus. J. Colloid Interface Sci..

[42] Subramanian S., Baidal D., Feingold K.R., Anawalt B., Blackman M.R. (2000). The Management of Type 1 Diabetes. Endotext [Internet].

[43] McCall A.L. (2012). Insulin therapy and hypoglycemia. Endocrinol. Metab. Clin. N. Am..

[44] Russell-Jones D., Khan R. (2007). Insulin-associated weight gain in diabetes--causes, effects and coping strategies. Diabetes Obes. Metab..

[45] Kueh M.T.W., Chew N.W.S., Al-Ozairi E., le Roux C.W. (2024). The emergence of obesity in type 1 diabetes. Int. J. Obes..

[46] Wallace A.S., Chang A.R., Shin J.I., Reider J., Echouffo-Tcheugui J.B., Grams M.E., Selvin E. (2022). Obesity and Chronic Kidney Disease in US Adults With Type 1 and Type 2 Diabetes Mellitus. J. Clin. Endocrinol. Metab..

[47] Fellinger P., Fuchs D., Wolf P., Heinze G., Luger A., Krebs M., Winhofer Y. (2019). Overweight and obesity in type 1 diabetes equal those of the general population. Wien. Klin. Wochenschr..

[48] Popovic D.S., Papanas N. (2022). Double Diabetes: A Growing Problem Requiring Solutions. Exp. Clin. Endocrinol. Diabetes.

[49] Kietsiriroje N., Pearson S., Campbell M., Ariëns R.A.S., Ajjan R.A. (2019). Double diabetes: A distinct high-risk group?. Diabetes Obes. Metab..

[50] Karatas S., Beysel S. (2021). Visceral Adiposity Index, Triglyceride/High-Density Lipoprotein Ratio, and Lipid Accumulation Product Index to Discriminate Metabolic Syndrome Among Adult Type 1 Diabetes Patients. Metab. Syndr. Relat. Disord..

[51] Foster N.C., Beck R.W., Miller K.M., Clements M.A., Rickels M.R., DiMeglio L.A., Maahs D.M., Tamborlane W.V., Bergenstal R., Smith E. (2019). State of Type 1 Diabetes Management and Outcomes from the T1D Exchange in 2016–2018. Diabetes Technol. Ther..

[52] Akturk H.K., Rewers A., Joseph H., Schneider N., Garg S.K. (2018). Possible Ways to Improve Postprandial Glucose Control in Type 1 Diabetes. Diabetes Technol. Ther..

[53] Yardley J.E., Sigal R.J. (2015). Exercise strategies for hypoglycemia prevention in individuals with type 1 diabetes. Diabetes Spectr..

[54] Infante M., Baidal D.A., Rickels M.R., Fabbri A., Skyler J.S., Alejandro R., Ricordi C. (2021). Dual-hormone artificial pancreas for management of type 1 diabetes: Recent progress and future directions. Artif. Organs.

[55] Talbo M.K., Katz A., Hill L., Peters T.M., Yale J.F., Brazeau A.S. (2023). Effect of diabetes technologies on the fear of hypoglycaemia among people living with type 1 diabetes: A systematic review and meta-analysis. eClinicalMedicine.

[56] Cho M.K., Kim M.Y. (2021). What Affects Quality of Life for People with Type 1 Diabetes?: A Cross-Sectional Observational Study. Int. J. Environ. Res. Public Health.

[57] Hanlan M.E., Griffith J., Patel N., Jaser S.S. (2013). Eating Disorders and Disordered Eating in Type 1 Diabetes: Prevalence, Screening, and Treatment Options. Curr. Diabetes Rep..

[58] NCD Risk Factor Collaboration (NCD-RisC) (2024). Worldwide trends in underweight and obesity from 1990 to 2022: A pooled analysis of 3663 population-representative studies with 222 million children, adolescents, and adults. Lancet.

[59] Novelli G., Cassadonte C., Sbraccia P., Biancolella M. (2023). Genetics: A Starting Point for the Prevention and the Treatment of Obesity. Nutrients.

[60] The Lancet Diabetes Endocrinology (2022). Childhood obesity: A growing pandemic. Lancet Diabetes Endocrinol..

[61] Bielka W., Przezak A., Molęda P., Pius-Sadowska E., Machaliński B. (2024). Double diabetes-when type 1 diabetes meets type 2 diabetes: Definition, pathogenesis and recognition. Cardiovasc. Diabetol..

[62] Freeby M., Lane K. (2024). Treating obesity in type 1 diabetes mellitus—Review of efficacy and safety. Curr. Opin. Endocrinol. Diabetes Obes..

[63] Bailey R., Calhoun P., Garg S.K. (2024). Weight Gain and Glycemic Control in Adults with Type 1 Diabetes in the T1D Exchange Registry. Diabetes Technol. Ther..

[64] Edqvist J., Rawshani A., Adiels M., Björck L., Lind M., Svensson A.M., Gudbjörnsdottir S., Sattar N., Rosengren A. (2019). BMI, Mortality, and Cardiovascular Outcomes in Type 1 Diabetes: Findings Against an Obesity Paradox. Diabetes Care.

[65] Melin E.O., Thulesius H.O., Hillman M., Landin-Olsson M., Thunander M. (2018). Abdominal obesity in type 1 diabetes associated with gender, cardiovascular risk factors and complications, and difficulties achieving treatment targets: A cross sectional study at a secondary care diabetes clinic. BMC Obes..

[66] Sun R., Wang J., Li M., Li J., Pan Y., Liu B., Lip G.Y.H., Zhang L. (2024). Association of Insulin Resistance With Cardiovascular Disease and All-Cause Mortality in Type 1 Diabetes: Systematic Review and Meta-analysis. Diabetes Care.

[67] Schauer I.E., Snell-Bergeon J.K., Bergman B.C., Maahs D.M., Kretowski A., Eckel R.H., Rewers M. (2011). Insulin resistance, defective insulin-mediated fatty acid suppression, and coronary artery calcification in subjects with and without type 1 diabetes: The CACTI study. Diabetes.

[68] Cantley N.W., Lonnen K., Kyrou I., Tahrani A.A., Kahal H. (2021). The association between overweight/obesity and double diabetes in adults with type 1 diabetes; a cross-sectional study. BMC Endocr. Disord..

[69] Price S.A., Gorelik A., Fourlanos S., Colman P.G., Wentworth J.M. (2014). Obesity is associated with retinopathy and macrovascular disease in type 1 diabetes. Obes. Res. Clin. Pract..

[70] Pozzilli P., Pieralice S. (2018). Latent Autoimmune Diabetes in Adults: Current Status and New Horizons. Endocrinol. Metab..

[71] Buzzetti R., Tuomi T., Mauricio D., Pietropaolo M., Zhou Z., Pozzilli P., Leslie R.D. (2020). Management of Latent Autoimmune Diabetes in Adults: A Consensus Statement From an International Expert Panel. Diabetes.

[72] Lohmann T., Nietzschmann U., Kiess W. (2000). “Lady-like”: Is there a latent autoimmune diabetes in the young?. Diabetes Care.

[73] O’Neal K.S., Johnson J.L., Panak R.L. (2016). Recognizing and Appropriately Treating Latent Autoimmune Diabetes in Adults. Diabetes Spectr..

[74] Klemann C., Wagner L., Stephan M., von Hörsten S. (2016). Cut to the chase: A review of CD26/dipeptidyl peptidase-4’s (DPP4) entanglement in the immune system. Clin. Exp. Immunol..

[75] Liu Q.K. (2024). Mechanisms of action and therapeutic applications of GLP-1 and dual GIP/GLP-1 receptor agonists. Front. Endocrinol..

[76] Hammoud R., Drucker D.J. (2023). Beyond the pancreas: Contrasting cardiometabolic actions of GIP and GLP1. Nat. Rev. Endocrinol..

[77] Seino Y., Fukushima M., Yabe D. (2010). GIP and GLP-1, the two incretin hormones: Similarities and differences. J. Diabetes Investig..

[78] Drucker D.J. (2018). Mechanisms of Action and Therapeutic Application of Glucagon-like Peptide-1. Cell Metab..

[79] El K., Campbell J.E. (2020). The role of GIP in α-cells and glucagon secretion. Peptides.

[80] Müller T.D., Finan B., Bloom S.R., D’Alessio D., Drucker D.J., Flatt P.R., Fritsche A., Gribble F., Grill H.J., Habener J.F. (2019). Glucagon-like peptide 1 (GLP-1). Mol. Metab..

[81] Corrao S., Pollicino C., Maggio D., Torres A., Argano C. (2024). Tirzepatide against obesity and insulin-resistance: Pathophysiological aspects and clinical evidence. Front. Endocrinol..

[82] Zheng Z., Zong Y., Ma Y., Tian Y., Pang Y., Zhang C., Gao J. (2024). Glucagon-like peptide-1 receptor: Mechanisms and advances in therapy. Signal Transduct. Target. Ther..

[83] Wang X., Yang X., Qi X., Fan G., Zhou L., Peng Z., Yang J. (2024). Anti-atherosclerotic effect of incretin receptor agonists. Front. Endocrinol..

[84] Sattar N., Lee M.M.Y., Kristensen S.L., Branch K.R.H., Del Prato S., Khurmi N.S., Lam C.S.P., Lopes R.D., McMurray J.J.V., Pratley R.E. (2021). Cardiovascular, mortality, and kidney outcomes with GLP-1 receptor agonists in patients with type 2 diabetes: A systematic review and meta-analysis of randomised trials. Lancet Diabetes Endocrinol..

[85] Farilla L., Hui H., Bertolotto C., Kang E., Bulotta A., Di Mario U., Perfetti R. (2002). Glucagon-like peptide-1 promotes islet cell growth and inhibits apoptosis in Zucker diabetic rats. Endocrinology.

[86] Stoffers D.A., Kieffer T.J., Hussain M.A., Drucker D.J., Bonner-Weir S., Habener J.F., Egan J.M. (2000). Insulinotropic glucagon-like peptide 1 agonists stimulate expression of homeodomain protein IDX-1 and increase islet size in mouse pancreas. Diabetes.

[87] Xu G., Stoffers D.A., Habener J.F., Bonner-Weir S. (1999). Exendin-4 stimulates both beta-cell replication and neogenesis, resulting in increased beta-cell mass and improved glucose tolerance in diabetic rats. Diabetes.

[88] Juang J.H., Kuo C.H., Wu C.H., Juang C. (2008). Exendin-4 treatment expands graft beta-cell mass in diabetic mice transplanted with a marginal number of fresh islets. Cell Transplant..

[89] Trümper A., Trümper K., Trusheim H., Arnold R., Göke B., Hörsch D. (2001). Glucose-dependent insulinotropic polypeptide is a growth factor for beta (INS-1) cells by pleiotropic signaling. Mol. Endocrinol..

[90] Trümper A., Trümper K., Hörsch D. (2002). Mechanisms of mitogenic and anti-apoptotic signaling by glucose-dependent insulinotropic polypeptide in beta(INS-1)-cells. J. Endocrinol..

[91] Schmitz S.H., Aronne L.J. (2024). Using second-generation anti-obesity medications. Diabetes Spectrum..

[92] Chavda V.P., Ajabiya J., Teli D., Bojarska J., Apostolopoulos V. (2022). Tirzepatide, a New Era of Dual-Targeted Treatment for Diabetes and Obesity: A Mini-Review. Molecules.

[93] FDA Approves First Treatment to Reduce Risk of Serious Heart Problems Specifically in Adults with Obesity or Overweight. https://www.fda.gov/news-events/press-announcements/fda-approves-first-treatment-reduce-risk-serious-heart-problems-specifically-adults-obesity-or.

[94] FDA Approves First Medication for Obstructive Sleep Apnea. https://www.fda.gov/news-events/press-announcements/fda-approves-first-medication-obstructive-sleep-apnea.

[95] Malhotra A., Grunstein R.R., Fietze I., Weaver T.E., Redline S., Azarbarzin A., Sands S.A., Schwab R.J., Dunn J.P., Chakladar S. (2024). Tirzepatide for the Treatment of Obstructive Sleep Apnea and Obesity. N. Engl. J. Med..

[96] Kuhadiya N.D., Malik R., Bellini N.J., Patterson J.L., Traina A., Makdissi A., Dandona P. (2013). Liraglutide as additional treatment to insulin in obese patients with type 1 diabetes mellitus. Endocr. Pract..

[97] Anson M., Zhao S.S., Austin P., Ibarburu G.H., Malik R.A., Alam U. (2023). SGLT2i and GLP-1 RA therapy in type 1 diabetes and reno-vascular outcomes: A real-world study. Diabetologia.

[98] Mathieu C., Zinman B., Hemmingsson J.U., Woo V., Colman P., Christiansen E., Linder M., Bode B., ADJUNCT ONE Investigators (2016). Efficacy and Safety of Liraglutide Added to Insulin Treatment in Type 1 Diabetes: The ADJUNCT ONE Treat-To-Target Randomized Trial. Diabetes Care.

[99] Ahrén B., Hirsch I.B., Pieber T.R., Mathieu C., Gómez-Peralta F., Hansen T.K., Philotheou A., Birch S., Christiansen E., Jensen T.J. (2016). Efficacy and Safety of Liraglutide Added to Capped Insulin Treatment in Subjects With Type 1 Diabetes: The ADJUNCT TWO Randomized Trial. Diabetes Care.

[100] Dejgaard T.F., Frandsen C.S., Kielgast U., Størling J., Overgaard A.J., Svane M.S., Olsen M.H., Thorsteinsson B., Andersen H.U., Krarup T. (2024). Liraglutide enhances insulin secretion and prolongs the remission period in adults with newly diagnosed type 1 diabetes (the NewLira study): A randomized, double-blind, placebo-controlled trial. Diabetes Obes. Metab..

[101] Pozzilli P., Leslie R.D., Peters A.L., Buzzetti R., Shankar S.S., Milicevic Z., Pavo I., Lebrec J., Martin S., Schloot N.C. (2018). Dulaglutide treatment results in effective glycaemic control in latent autoimmune diabetes in adults (LADA): A post-hoc analysis of the AWARD-2, -4 and -5 Trials. Diabetes Obes. Metab..

[102] Sofrà D., Beer S. (2019). Impact of adding semaglutide in a person with type 1 diabetes: A case report. Diabetes Updates.

[103] Raven L.M., Greenfield J.R., Muir C.A. (2023). Glucagon-like Peptide-1 Receptor Agonist Treatment with Semaglutide in Type 1 Diabetes. JCEM Case Rep..

[104] Dhillon S. (2018). Semaglutide: First Global Approval. Drugs.

[105] Pasqua M.R., Tsoukas M.A., Kobayati A., Aboznadah W., Jafar A., Haidar A. (2025). Subcutaneous weekly semaglutide with automated insulin delivery in type 1 diabetes: A double-blind, randomized, crossover trial. Nat. Med..

[106] Lakshman R., Boughton C., Hovorka R. (2023). The changing landscape of automated insulin delivery in the management of type 1 diabetes. Endocr. Connect..

[107] Pasqua M.R., Jafar A., Kobayati A., Tsoukas M.A., Haidar A. (2023). Low-Dose Empagliflozin as Adjunct to Hybrid Closed-Loop Insulin Therapy in Adults With Suboptimally Controlled Type 1 Diabetes: A Randomized Crossover Controlled Trial. Diabetes Care.

[108] Battelino T., Danne T., Bergenstal R.M., Amiel S.A., Beck R., Biester T., Bosi E., Buckingham B.A., Cefalu W.T., Close K.L. (2019). Clinical Targets for Continuous Glucose Monitoring Data Interpretation: Recommendations From the International Consensus on Time in Range. Diabetes Care.

[109] Fonseca V.A., Capehorn M.S., Garg S.K., Jódar Gimeno E., Hansen O.H., Holst A.G., Nayak G., Seufert J. (2019). Reductions in Insulin Resistance are Mediated Primarily via Weight Loss in Subjects With Type 2 Diabetes on Semaglutide. J. Clin. Endocrinol. Metab..

[110] Navodnik M.P., Janež A., Žuran I. (2023). The Effect of Additional Treatment with Empagliflozin or Semaglutide on Endothelial Function and Arterial Stiffness in Subjects with Type 1 Diabetes Mellitus-ENDIS Study. Pharmaceutics.

[111] Mohandas D., Calma J., Gao C., Basina M. (2023). Evaluating the Efficacy and Safety of Long-Acting GLP-1 Receptor Agonists in T1DM Patients. Endocrines.

[112] Dandona P., Chaudhuri A., Ghanim H. (2023). Semaglutide in Early Type 1 Diabetes. N. Engl. J. Med..

[113] Nassar M., Chaudhuri A., Ghanim H., Dandona P. (2024). Glucagon-like peptide-1 receptor agonists as a possible intervention to delay the onset of type 1 diabetes: A new horizon. World J. Diabetes.

[114] Gaglia J.L., Laffel L.M., Patti M.E. (2024). More on Semaglutide in Early Type 1 Diabetes. N. Engl. J. Med..

[115] Grassi B.A., Teresa Onetto M., Sánchez C., Tapia N., Mena F. (2024). Effect of low dose Semaglutide in people with Type 1 Diabetes and excess weight. Diabetes Res. Clin. Pract..

[116] Garg S.K., Kaur G., Haider Z., Rodriquez E., Beatson C., Snell-Bergeon J. (2024). Efficacy of Semaglutide in Overweight and Obese Patients with Type 1 Diabetes. Diabetes Technol. Ther..

[117] Almohareb S.N., Alfayez O.M., Aljuaid S.S., Alshahrani W.A., Bakhsh G., Alshammari M.K., Al Yami M.S., Alshaya O.A., Alomran A.S., Korayem G.B. (2024). Effectiveness and Safety of GLP-1 Receptor Agonists in Patients with Type 1 Diabetes. J. Clin. Med..

[118] Orrange S., Humphrey T., Fayne E., Peters A. (2024). Semaglutide for Weight Reduction in Type 1 Diabetes: Promising Results with Uncertain Glycemic Impact. J. Diabetes Sci. Technol..

[119] Cohen N., Yeung A., Jenkins A. (2025). Real-world experience of adjunct weekly semaglutide in Type 1 diabetes. Is it worth it?. Diabetes Res. Clin. Pract..

[120] Zheng X., Huang B., Luo S., Yang D., Bao W., Li J., Yao B., Weng J., Yan J. (2017). A new model to estimate insulin resistance via clinical parameters in adults with type 1 diabetes. Diabetes Metab. Res. Rev..

[121] Wong G., Garner E.M., Srivastava G. (2023). Combined GLP-1 Receptor Agonist and Amylin Analogue Pharmacotherapy to Treat Obesity Comorbid With Type 1 Diabetes. JCEM Case Rep..

[122] Gad H., Malik R.A. (2024). Rapid Improvement in Weight, Body Composition, and Glucose Variability With Semaglutide in Type 1 Diabetes. Cureus.

[123] Gong J.Y., Wentworth J.M. (2024). Resolution of symptoms of binge eating disorder associated with semaglutide treatment for obesity and type 1 diabetes. Intern. Med. J..

[124] Da Porto A., Varisco E., Antonello M., Casarsa V., Sechi L.A. (2024). Semaglutide Treatment in Adult-Onset Autoimmune Diabetes: A Case Study With Long-Term Follow-Up and Periodic Evaluation of Beta-Cell Function. Cureus.

[125] Akturk H.K., Dong F., Snell-Bergeon J.K., Karakus K.E., Shah V.N. (2024). Efficacy and Safety of Tirzepatide in Adults with Type 1 Diabetes: A Proof of Concept Observational Study. J. Diabetes Sci. Technol..

[126] Garg S.K., Akturk H.K., Kaur G., Beatson C., Snell-Bergeon J. (2024). Efficacy and Safety of Tirzepatide in Overweight and Obese Adult Patients with Type 1 Diabetes. Diabetes Technol. Ther..

[127] Karakus K.E., Klein M.P., Akturk H.K., Shah V.N. (2024). Changes in Basal and Bolus Insulin Requirements with Tirzepatide as an Adjunctive Therapy in Adults with Type 1 Diabetes Using Tandem Control-IQ. Diabetes Ther..

[128] Rivera Gutierrez R., Tama E., Bechenati D., Castañeda Hernandez R., Bennett P.K., McNally A.W., Fansa S., Anazco D., Acosta A., Hurtado Andrade M.D. (2024). Effect of Tirzepatide on Body Weight and Diabetes Control in Adults With Type 1 Diabetes and Overweight or Obesity. Mayo Clin. Proc..

[129] Sharma P.P., Ramirez-Berlioz A.M., Weisz A.D. (2023). Diabetic Ketoacidosis in a Patient With Type I Diabetes Treated With a Closed-Loop Sensor-Augmented Insulin Infusion System. AACE Clin. Case Rep..

[130] Nose H., Mack G.W., Shi X., Nadel E.R., Marriott B.M. (1994). Shift in Body Fluid Compartments After Dehydration in Humans. Institute of Medicine (US) Committee on Military Nutrition Research. Fluid Replacement and Heat Stress.

[131] Mendoza F., Parsiani R. (2023). Impact of tirzepatide in a patient with type 1 diabetes and obesity: A case report. J. Am. Pharm. Assoc. (2003).

[132] Ahmed K., Koubeh S., Ahmed A., Hussain S. (2024). Cardiorenal benefit of tirzepatide in type 1 diabetes: A case report. J Diabetol.

[133] Peters A.L., Buzzetti R., Lee C.J., Pavo I., Liu M., Karanikas C.A., Paik J.S. (2024). Improved HbA1c and body weight in GADA-positive individuals treated with tirzepatide: A post hoc analysis of SURPASS. J. Clin. Endocrinol. Metab..

[134] Suzuki Y. (2024). Case report: Tirzepatide for mitochondrial diabetes mellitus with a mutation at position 3271 and transient GAD antibody positivity. Diabetes Res. Clin. Pract..

[135] Suzuki Y., Taniyama M., Shimada A., Atumi Y., Matsuoka K., Oka Y. (2002). GAD antibody in mitochondrial diabetes associated with tRNA(UUR) mutation at position 3271. Diabetes Care.

[136] Snell-Bergeon J.K., Kaur G., Renner D., Akturk H.K., Beatson C., Garg S.K. (2025). Effectiveness of Semaglutide and Tirzepatide in Overweight and Obese Adults with Type 1 Diabetes. Diabetes Technol. Ther..

[137] Rodriguez P.J., Goodwin Cartwright B.M., Gratzl S., Brar R., Baker C., Gluckman T.J., Stucky N.L. (2024). Semaglutide vs. Tirzepatide for Weight Loss in Adults With Overweight or Obesity. JAMA Intern. Med..

[138] FDA Approves Once-Weekly Wegovy^®^ Injection for the Treatment of Obesity in Teens Aged 12 Years and Older. https://www.novonordisk-us.com/media/news-archive/news-details.html?id=151389.

[139] Seetharaman S., Cengiz E. (2024). Expectations and Outcomes from Glucagon-Like Peptide-1 Receptor Agonists as Adjunct Treatment for Type 1 Diabetes—Case Presentations. J. Diabetes Sci. Technol..

[140] Austin G.O., Tomas A. (2023). Variation in responses to incretin therapy: Modifiable and non-modifiable factors. Front. Mol. Biosci..

[141] Klein M.P., Akturk H.K., Snell-Bergeon J.K., Shah V.N. (2024). Reduced Efficacy of Glucagon-Like Peptide-1 Receptor Agonists Therapy in People With Type 1 Diabetes and Genetic Forms of Obesity. J. Diabetes Sci. Technol..

[142] Blackburn G. (1995). Effect of degree of weight loss on health benefits. Obes. Res..

[143] Pinheiro M.M., de Souza L.G., Nunes G.P., Martin I.F., de Oliveira Y.U., Pinheiro F.M.M., Costa L.N., Caprio M., Della-Morte D., Infante M. (2024). The first report of leukocytoclastic vasculitis induced by once-weekly subcutaneous semaglutide. Curr. Med. Res. Opin..

[144] Ouellette S., Frias G., Shah R., Alamgir M., Wassef C. (2023). Dermal Hypersensitivity Reaction to Semaglutide: Two Case Reports. J. Drugs Dermatol..

[145] Le T.T.B., Minh L.H.N., Devi P., Islam N., Sachmechi I. (2024). A Case Report of Systemic Allergic Reaction to the Dual Glucose-Dependent Insulinotropic Polypeptide/Glucagon-Like Peptide-1 Receptor Agonist Tirzepatide. Cureus.

[146] Li P., Li Z., Staton E., Umpierrez G.E., Davis G., Shao H., Pasquel F.J. (2024). GLP-1 Receptor Agonist and SGLT2 Inhibitor Prescribing in People With Type 1 Diabetes. JAMA.

[147] Effect of Weekly GLP1 Agonist Treatment in “double Diabetes” (TOLEDDO). ClinicalTrials.gov ID: NCT05305794. NCT05305794.

[148] Tirzepatide for the Treatment of Concurrent Type 1 Diabetes and Overweight or Obesity (TZP-T1D). ClinicalTrials.gov ID: NCT06180616. NCT06180616.

[149] GIP/GLP-1RA as Adjunctive to Automated Insulin Delivery in Adults With Type 1 Diabetes (AID-JUNCT). ClinicalTrials.gov ID: NCT06630585. NCT06630585.

[150] Cichocka E., Maj-Podsiadło A., Gumprecht J. (2024). Polycystic ovary syndrome and type 1 diabetes—The current state of knowledge. Endokrynol. Pol..

[151] Anala A.D., Saifudeen I.S.H., Ibrahim M., Nanda M., Naaz N., Atkin S.L. (2023). The Potential Utility of Tirzepatide for the Management of Polycystic Ovary Syndrome. J. Clin. Med..

[152] Dai C., Walker J.T., Shostak A., Padgett A., Spears E., Wisniewski S., Poffenberger G., Aramandla R., Dean E.D., Prasad N. (2020). Tacrolimus- and sirolimus-induced human β cell dysfunction is reversible and preventable. JCI Insight.

[153] Yesildag B., Mir-Coll J., Neelakandhan A., Gibson C.B., Perdue N.R., Rufer C., Karsai M., Biernath A., Forschler F., Jin P.W. (2022). Liraglutide protects β-cells in novel human islet spheroid models of type 1 diabetes. Clin. Immunol..

[154] Li L.R., Jia X.L., Hui H., Zhang J., Liu Y., Cui W.J., Xu Q.Y., Zhu D.L. (2016). Liraglutide Enhances the Efficacy of Human Mesenchymal Stem Cells in Preserving Islet β-cell Function in Severe Non-obese Diabetic Mice. Mol. Med..

[155] Kachapati K., Adams D., Bednar K., Ridgway W.M. (2012). The non-obese diabetic (NOD) mouse as a model of human type 1 diabetes. Methods Mol. Biol..

[156] Ben Nasr M., Usuelli V., Dellepiane S., Seelam A.J., Fiorentino T.V., D’Addio F., Fiorina E., Xu C., Xie Y., Balasubramanian H.B. (2024). Glucagon-like peptide 1 receptor is a T cell-negative costimulatory molecule. Cell Metab..

[157] Wu L., Carlino M.S., Brown D.A., Long G.V., Clifton-Bligh R., Mellor R., Moore K., Sasson S.C., Menzies A.M., Tsang V. (2024). Checkpoint Inhibitor-Associated Autoimmune Diabetes Mellitus Is Characterized by C-peptide Loss and Pancreatic Atrophy. J. Clin. Endocrinol. Metab..

[158] Locatelli J.C., Costa J.G., Haynes A., Naylor L.H., Fegan P.G., Yeap B.B., Green D.J. (2024). Incretin-Based Weight Loss Pharmacotherapy: Can Resistance Exercise Optimize Changes in Body Composition?. Diabetes Care.

[159] Prado C.M., Phillips S.M., Gonzalez M.C., Heymsfield S.B. (2024). Muscle matters: The effects of medically induced weight loss on skeletal muscle. Lancet Diabetes Endocrinol..

[160] Koon-Yee Lee G., Chun-Ming Au P., Hoi-Yee Li G., Chan M., Li H.L., Man-Yung Cheung B., Chi-Kei Wong I., Ho-Fun Lee V., Mok J., Hon-Kei Yip B. (2021). Sarcopenia and mortality in different clinical conditions: A meta-analysis. Osteoporos Sarcopenia.

[161] Nunn E., Jaiswal N., Gavin M., Uehara K., Stefkovich M., Drareni K., Calhoun R., Lee M., Holman C.D., Baur J.A. (2024). Antibody blockade of activin type II receptors preserves skeletal muscle mass and enhances fat loss during GLP-1 receptor agonism. Mol. Metab..

[162] Denroche H.C., Verchere C.B. (2018). IAPP and type 1 diabetes: Implications for immunity, metabolism and islet transplants. J. Mol. Endocrinol..

[163] Scherbaum W.A. (1998). The role of amylin in the physiology of glycemic control. Exp. Clin. Endocrinol. Diabetes.

[164] Young A.A. (1997). Amylin’s physiology and its role in diabetes. Curr. Opin. Endocrinol. Diabetes.

[165] Bronský J., Chada M., Kotaska K., Průsa R. (2002). Amylin—Its physiological role in humans. Cesk Fysiol..

[166] Riddle M.C. (2020). Rediscovery of the Second β-Cell Hormone: Co-replacement With Pramlintide and Insulin in Type 1 Diabetes. Diabetes Care.

[167] Lutz T.A. (2009). Control of food intake and energy expenditure by amylin-therapeutic implications. Int. J. Obes..

[168] Kruger D.F., Gatcomb P.M., Owen S.K. (1999). Clinical implications of amylin and amylin deficiency. Diabetes Educ..

[169] Unger R.H., Cherrington A.D. (2012). Glucagonocentric restructuring of diabetes: A pathophysiologic and therapeutic makeover. J. Clin. Investig..

[170] Fredheim S., Andersen M.L., Pörksen S., Nielsen L.B., Pipper C., Hansen L., Holst J.J., Thomsen J., Johannesen J., Mortensen H.B. (2015). The influence of glucagon on postprandial hyperglycaemia in children 5 years after onset of type 1 diabetes. Diabetologia.

[171] Weyer C., Maggs D.G., Young A.A., Kolterman O.G. (2001). Amylin replacement with pramlintide as an adjunct to insulin therapy in type 1 and type 2 diabetes mellitus: A physiological approach toward improved metabolic control. Curr. Pharm. Des..

[172] Edelman S.V., Caballero L. (2006). Amylin replacement therapy in patients with type 1 diabetes. Diabetes Educ..

[173] Ryan G.J., Jobe L.J., Martin R. (2005). Pramlintide in the treatment of type 1 and type 2 diabetes mellitus. Clin. Ther..

[174] Liberini C.G., Koch-Laskowski K., Shaulson E., McGrath L.E., Lipsky R.K., Lhamo R., Ghidewon M., Ling T., Stein L.M., Hayes M.R. (2019). Combined Amylin/GLP-1 pharmacotherapy to promote and sustain long-lasting weight loss. Sci. Rep..

[175] Sidrak W.R., Kalra S., Kalhan A. (2024). Approved and Emerging Hormone-Based Anti-Obesity Medications: A Review Article. Indian J. Endocrinol. Metab..

[176] Bisgaard Bengtsen M., Møller N. (2021). Mini-review: Glucagon responses in type 1 diabetes—A matter of complexity. Physiol. Rep..

[177] Zhang L., Qin Y., Huang Y., Hu Q., Wu Q., Wang X., Zhang M. (2024). Abnormal late postprandial glucagon response in type 1 diabetes is a function of differences in stimulated C-peptide concentrations. Front. Endocrinol..

[178] Oboza P., Ogarek N., Olszanecka-Glinianowicz M., Kocelak P. (2023). Can type 1 diabetes be an unexpected complication of obesity?. Front. Endocrinol..

[179] Martyn-Nemeth P., Quinn L., Penckofer S., Park C., Hofer V., Burke L. (2017). Fear of hypoglycemia: Influence on glycemic variability and self-management behavior in young adults with type 1 diabetes. J. Diabetes Complicat..

[180] Dziewa M., Bańka B., Herbet M., Piątkowska-Chmiel I. (2023). Eating Disorders and Diabetes: Facing the Dual Challenge. Nutrients.

[181] Corbin K.D., Driscoll K.A., Pratley R.E., Smith S.R., Maahs D.M., Mayer-Davis E.J. (2018). Obesity in Type 1 Diabetes: Pathophysiology, Clinical Impact, and Mechanisms. Endocr. Rev..

[182] Gregory J.M., Cherrington A.D., Moore D.J. (2020). The Peripheral Peril: Injected Insulin Induces Insulin Insensitivity in Type 1 Diabetes. Diabetes.

[183] Wolosowicz M., Lukaszuk B., Chabowski A. (2020). The Causes of Insulin Resistance in Type 1 Diabetes Mellitus: Is There a Place for Quaternary Prevention?. Int. J. Environ. Res. Public Health.

[184] Marques C.L., Beretta M.V., Prates R.E., de Almeida J.C., da Costa Rodrigues T. (2023). Body adiposity markers and insulin resistance in patients with type 1 diabetes. Arch. Endocrinol. Metab..

[185] Kolb H., Stumvoll M., Kramer W., Kempf K., Martin S. (2018). Insulin translates unfavourable lifestyle into obesity. BMC Med..

[186] Szukiewicz D. (2023). Molecular Mechanisms for the Vicious Cycle between Insulin Resistance and the Inflammatory Response in Obesity. Int. J. Mol. Sci..

[187] Purnell J.Q., Hokanson J.E., Marcovina S.M., Steffes M.W., Cleary P.A., Brunzell J.D. (1998). Effect of excessive weight gain with intensive therapy of type 1 diabetes on lipid levels and blood pressure: Results from the DCCT. Diabetes Control and Complications Trial. JAMA.

[188] Everson S.A., Goldberg D.E., Helmrich S.P., Lakka T.A., Lynch J.W., Kaplan G.A., Salonen J.T. (1998). Weight gain and the risk of developing insulin resistance syndrome. Diabetes Care.

[189] Wondmkun Y.T. (2020). Obesity, Insulin Resistance, and Type 2 Diabetes: Associations and Therapeutic Implications. Diabetes Metab. Syndr. Obes..

[190] Wilkin T.J. (2001). The accelerator hypothesis: Weight gain as the missing link between Type I and Type II diabetes. Diabetologia.

[191] Purnell J.Q., Braffett B.H., Zinman B., Gubitosi-Klug R.A., Sivitz W., Bantle J.P., Ziegler G., Cleary P.A., Brunzell J.D. (2017). Impact of Excessive Weight Gain on Cardiovascular Outcomes in Type 1 Diabetes: Results from the Diabetes Control and Complications Trial/Epidemiology of Diabetes Interventions and Complications (DCCT/EDIC) Study. Diabetes Care.

[192] Cleland S.J., Fisher B.M., Colhoun H.M., Sattar N., Petrie J.R. (2013). Insulin resistance in type 1 diabetes: What is ‘double diabetes’ and what are the risks?. Diabetologia.

[193] Heise T., Mari A., DeVries J.H., Urva S., Li J., Pratt E.J., Coskun T., Thomas M.K., Mather K.J., Haupt A. (2022). Effects of subcutaneous tirzepatide versus placebo or semaglutide on pancreatic islet function and insulin sensitivity in adults with type 2 diabetes: A multicentre, randomised, double-blind, parallel-arm, phase 1 clinical trial. Lancet Diabetes Endocrinol..

[194] Lee C.J., Mao H., Thieu V.T., Landó L.F., Thomas M.K. (2023). Tirzepatide as Monotherapy Improved Markers of Beta-cell Function and Insulin Sensitivity in Type 2 Diabetes (SURPASS-1). J. Endocr. Soc..

[195] Thomas M.K., Nikooienejad A., Bray R., Cui X., Wilson J., Duffin K., Milicevic Z., Haupt A., Robins D.A. (2021). Dual GIP and GLP-1 Receptor Agonist Tirzepatide Improves Beta-cell Function and Insulin Sensitivity in Type 2 Diabetes. J. Clin. Endocrinol. Metab..

[196] Samms R.J., Christe M.E., Collins K.A., Pirro V., Droz B.A., Holland A.K., Friedrich J.L., Wojnicki S., Konkol D.L., Cosgrove R. (2021). GIPR agonism mediates weight-independent insulin sensitization by tirzepatide in obese mice. J. Clin. Investig..

[197] Rawshani A., Sattar N., Franzén S., Hattersley A.T., Svensson A.M., Eliasson B., Gudbjörnsdottir S. (2018). Excess mortality and cardiovascular disease in young adults with type 1 diabetes in relation to age at onset: A nationwide, register-based cohort study. Lancet.

[198] Rossing P., Groop P.H., Singh R., Lawatscheck R., Tuttle K.R. (2024). Prevalence of Chronic Kidney Disease in Type 1 Diabetes Among Adults in the U.S.. Diabetes Care.

[199] Greenfield J.R., Frampton R., Millard K., Snaith J.R. (2025). Use of Cardioprotective Adjuncts in Type 1 Diabetes. Diabetes Ther..

[200] Vergès B. (2024). Cardiovascular disease in type 1 diabetes, an underestimated danger: Epidemiological and pathophysiological data. Atherosclerosis.

[201] Bjornstad P., Cherney D., Maahs D.M. (2014). Early diabetic nephropathy in type 1 diabetes: New insights. Curr. Opin. Endocrinol. Diabetes Obes..

[202] Orchard T.J., Chang Y.F., Ferrell R.E., Petro N., Ellis D.E. (2002). Nephropathy in type 1 diabetes: A manifestation of insulin resistance and multiple genetic susceptibilities? Further evidence from the Pittsburgh Epidemiology of Diabetes Complication Study. Kidney Int..

[203] Yan M., Lin K., Huang D., Li J., Qu X., Chen K. (2024). Semaglutide attenuates pathological electrophysiological remodeling in diabetic cardiomyopathy via restoring Cx43 expression. Endocrine.

[204] Boengler K., Schulz R., Heusch G. (2006). Connexin 43 signalling and cardioprotection. Heart.

[205] Frampton R., Snaith J.R., Hocking S., Holmes-Walker J., Olsen N., Greenfield J.R. (2024). Reducing cardiometabolic risk with semaglutide in type 1 diabetes (RESET1): Study protocol of a phase 2 double-blinded randomised placebo-controlled trial. Diabet Med..

[206] Tougaard N.H., Theilade S., Winther S.A., Tofte N., Ahluwalia T.S., Hansen T.W., Rossing P., Frimodt-Møller M. (2020). Carotid-Femoral Pulse Wave Velocity as a Risk Marker for Development of Complications in Type 1 Diabetes Mellitus. J. Am. Heart Assoc..

[207] Mahase E. (2024). GLP-1 agonist shortage will last until end of 2024, government warns. BMJ.

[208] Sallam M., Snygg J., Allam D., Kassem R. (2024). Shifting the Scales: Tirzepatide’s Breakthrough in Obesity Management. Cureus.

[209] Green L., Taddei-Allen P. (2023). Shifting paradigms: Reframing coverage of antiobesity medications for plan sponsors. J. Manag. Care Spec. Pharm..

[210] Evans Kreider K., Pereira K., Padilla B.I. (2017). Practical Approaches to Diagnosing, Treating and Preventing Hypoglycemia in Diabetes. Diabetes Ther..

[211] Shahid W., Khan F., Makda A., Kumar V., Memon S., Rizwan A. (2020). Diabetic Ketoacidosis: Clinical Characteristics and Precipitating Factors. Cureus.

[212] Jarvis P.R.E. (2023). Euglycemic diabetic ketoacidosis: A potential pitfall for the emergency physician. Clin. Exp. Emerg. Med..

[213] Shah V.N., Peters A.L., Umpierrez G.E., Sherr J.L., Akturk H.K., Aleppo G., Bally L., Cengiz E., Cinar A., Dungan K. (2024). Consensus Report on Glucagon-Like Peptide-1 Receptor Agonists as Adjunctive Treatment for Individuals With Type 1 Diabetes Using an Automated Insulin Delivery System. J. Diabetes Sci. Technol..

[214] Nguyen K.T., Xu N.Y., Zhang J.Y., Shang T., Basu A., Bergenstal R.M., Castorino K., Chen K.Y., Kerr D., Koliwad S.K. (2022). Continuous Ketone Monitoring Consensus Report 2021. J. Diabetes Sci. Technol..

[215] Gorgojo-Martínez J.J., Mezquita-Raya P., Carretero-Gómez J., Castro A., Cebrián-Cuenca A., de Torres-Sánchez A., García-de-Lucas M.D., Núñez J., Obaya J.C., Soler M.J. (2022). Clinical Recommendations to Manage Gastrointestinal Adverse Events in Patients Treated with Glp-1 Receptor Agonists: A Multidisciplinary Expert Consensus. J. Clin. Med..

[216] Grosicki G.J., Dhurandhar N.V., Unick J.L., Arent S.M., Thomas J.G., Lofton H., Shepherd M.C., Kiel J., Coleman C., Jonnalagadda S.S. (2024). Sculpting Success: The Importance of Diet and Physical Activity to Support Skeletal Muscle Health during Weight Loss with New Generation Anti-Obesity Medications. Curr. Dev. Nutr..

[217] Kuźnik E., Dudkowiak R., Adamiec R., Poniewierka E. (2020). Diabetic autonomic neuropathy of the gastrointestinal tract. Prz Gastroenterol..

[218] Coleman S.E., Caswell N. (2020). Diabetes and eating disorders: An exploration of ‘Diabulimia’. BMC Psychol..

[219] Davidson J. (2014). Diabulimia: How eating disorders can affect adolescents with diabetes. Nurs. Stand..

