# Supplementary material for: Unveiling the Therapeutic Potential of the Second-Generation Incretin Analogs Semaglutide and Tirzepatide in Type 1 Diabetes and Latent Autoimmune Diabetes in Adults
Source: J Clin Med. 2025 Feb 15;14(4):1303. doi: 10.3390/jcm14041303 (PMC11856673; doi:10.3390/jcm14041303)
Supplement: Supplementary file 1 [file jcm-14-01303-s001.zip › jcm-3374466 - Supplementary Table S1.pdf]

**Table S1.** Clinical studies investigating the use of semaglutide in patients with autoimmune diabetes.

| Study Design | Study Population | Study Treatment and Duration | Main Findings |
|---|---|---|---|
| Case report (Sofrà and Beer; 2019 - Ref. 102) | • 32-year-old woman with overweight (BMI: 26.8 kg/m$^2$) and a 20-year history of T1D, who was treated with a patch insulin pump.<br>• Glucose control and comorbidities: HbA1c, 8.7%; non-proliferative diabetic retinopathy and maculopathy. | • Prescription: semaglutide prescribed as non-insulin adjunct therapy (6-month semaglutide therapy).<br>• Once-weekly subcutaneous semaglutide (starting dose: 0.25 mg/week for the first 4 weeks; afterwards, the weekly semaglutide dose was increased to 0.5 mg). | Flash glucose monitoring (FGM) metrics at 3 months from semaglutide initiation (vs. baseline):<br>• Average glucose level: 8.3 mmol/L (149 mg/dL) vs. 10.8 mmol/L (194 mg/dL) [-2.5 mmol/L (-45 mg/dL)].<br>• TIR 3.9-8.9 mmol/L (TIR 70-160 mg/dL): 63% vs. 38%.<br>• Percentage of time spent in hypoglycemia: 6% vs. 2%.<br>Glucose control, TDD of insulin, body weight and BMI at 6 months from the initiation of semaglutide therapy (vs. baseline):<br>• HbA1c: 6.8% vs. 8.7% (1.9% reduction).<br>• Fasting plasma glucose: 7.2 mmol/L (130 mg/dL) vs. 13.0 mmol/L (234 mg/dL) [-5.8 mmol/L (-104 mg/dL)].<br>• TDD of insulin: 20.8 U/day vs. 26 |

| | | | |
|---|---|---|---|
| | | | U/day (-5.2 U/day).<br>• Body weight loss of 5 kg (corresponding to an 8% body weight reduction), accompanied by a normalization of the BMI (24.8 kg/m$^2$ vs. 26.8 kg/m$^2$; -2.0 kg/m$^2$).<br><br><u>Side effects/adverse reactions</u>:<br><br>• Moderate gastrointestinal side effects (mostly nausea) were reported by the patient, particularly during the semaglutide titration phase, although such side effects did not require any treatment. |
| Prospective cohort study (Navodnik et al. 2023 - Ref. 110) | • ENDIS study (Endothelium dysfunction assessment study)<br>• 12-week intervention, prospective, randomized, single-center, controlled clinical study aimed to assess and compare the metabolic and endothelial function-related effects of | <u>Three study groups</u>:<br><br>• Group E (n = 30) received empagliflozin 10 mg/day.<br><br>• Group S (n = 30) received once-weekly subcutaneous semaglutide [semaglutide dose titration from 0.25 to 1 mg according to the recommendations | <u>Anthropometric parameters, laboratory parameters, FMD and PR at 12 weeks from treatment initiation</u>:<br><br>• Mean (±SD) body weight: E group, -2.49±2.69 kg; S group, -4.3±2.98 kg; vs. C group, -0.10±2.16 kg.<br>• Mean (±SD) waist circumference: E group: -4.0±5.5 cm; S group: -4.4±5.2 cm; vs. C group: -0.97±5.4 cm.<br>• Mean body weight and waist circumference decreased from baseline in |

| | | | |
|---|---|---|---|
| | empagliflozin and subcutaneous semaglutide as add-on treatments to insulin.<br><br>• Participants enrolled in the study: 91 adults with long-standing T1D on insulin therapy [either with multiple daily injection (MDI) insulin therapy or continuous subcutaneous insulin infusion (CSII)], all using continuous glucose monitoring (CGM) systems.<br><br>• 89 participants completed the study.<br><br>• 2 subjects withdrew from the study: one due to the side effects related to semaglutide (nausea and vomiting), and one in the control group due | from the SmPC or to the maximum tolerated dose]<br><br>• Group C (control group; n=29) received insulin therapy alone. | all groups: this reduction was statistically significant in both treatment groups as compared to baseline values and as compared to 12-week values in the control group ($p<0.05$).<br>• The reduction in mean (±SD) body weight values was significantly greater in the S group as compared to the E group ($p=0.020$).<br>• The reduction in mean (±SD) HbA1c values from baseline to week 12 was significant in the S group (-0.29±0.61%; $p=0.016$), but non-significant in the E group (-0.24±0.59%; $p=0.052$).<br>• Pairwise comparisons revealed a significant reduction in HbA1c in both treatment groups (E-C $p=0.015$, S-C $p=0.003$) as compared to the control group (C: 0.12±0.44%; $p=0.091$).<br>• There was no significant difference regarding changes in TIR 3.9-10.0 mmol/L (70-180 mg/dL) between E group and S group. |

| | | | |
|---|---|---|---|
| | to personal reasons.<br><br>• Demographic and baseline characteristics (age, sex, diabetes duration, smoking status, BMI, body weight, waist circumference, and estimated glomerular filtration rate based on cystatin C) were similar between groups.<br><br>• Only mean HbA1c values at baseline were significantly (but slightly) higher in the two treatment groups (p=0.002): empagliflozin group, 7.88%; semaglutide group, 7.42%; control group, 7.04%.<br><br>• Baseline characteristics of the study participants: mean | | • Participants in both treatment groups experienced a significant reduction in mean (±SD) TDD of insulin as compared to controls (-1.1±7.5 IU/day): S group, -8.5±9.3 IU/day (p=0.000); E group, -5.1±10.0 IU/day (p=0.022).<br>• There was no significant difference in mean TDD of insulin between the two treatment groups (p=0.114).<br>• There was a statistically significant reduction in mean (±SD) LDL cholesterol values in the S group from baseline (-0.30±0.68 mmol/L; p=0.017), and compared to controls (-0.15±0.51 mmol/L; S–C p=0.044) and to the E group (0.03±0.75 mmol/L; E-S p=0.001).<br>• There was a significant improvement in FMD (brachial artery flow-mediated dilation) in both intervention groups after 12 weeks, as compared to the baseline, with no changes observed between those two groups (p=0.745).<br>• In the E group, mean (±SD) FMD increased from 5.39±3.01% to 10.6±5.7% (2.0-fold; p=0.000) |

| | | |
|---|---|---|
| | (±SD) age [empagliflozin group, 48.2±10.7 years; semaglutide group, 48.5±9.6 years; control group, 47.0±12 years]; mean (±SD) diabetes duration [empagliflozin group, 21.3±10.2 years; semaglutide group, 21.9±11.2 years; control group, 18.8±11.9 years]; number and percentage of males [empagliflozin group, 18 (60%); semaglutide group, 19 (63.3%); control group, 17 (58.6%)]; mean (±SD) body weight [empagliflozin group, 81.9±14.2 kg; semaglutide group, 84.6±16.4 kg; control group, 82.6±11.7 kg]; mean (±SD) waist | • In the S group, mean (±SD) FMD increased from 5.81±3.14% to 11.1±4.9% (1.9-fold; p=0.000).<br>• Mean (±SD) peripheral resistance (PR) decreased in both intervention groups, although this reduction was statistically significant only in the S group (-0.07±0.23 mmHg/L/min; p=0.046).<br><br><u>Side effects/adverse reactions</u>:<br><br>• Only mild side effects were reported.<br>• There was only one case of semaglutide withdrawal due to persistent vomiting.<br>• Some patients reported nausea, but continued semaglutide treatment at a lower dose.<br>• No cases of severe hypoglycemia or diabetic ketoacidosis (DKA) were reported. |

| | | |
|---|---|---|
| | circumference [empagliflozin group, 99.9±11.7 cm; semaglutide group, 99.5±14.3 cm; control group, 98.5±9.9 cm]; mean (±SD) BMI [empagliflozin group, 27.7±4.3 kg/m$^2$; semaglutide group, 28.2±4.8 kg/m$^2$; control group, 26.9±3.0 kg/m$^2$]; mean (±SD) HbA1c [empagliflozin group, 7.88±0.72%; semaglutide group, 7.42±0.80%; control group, 7.04±0.90%]; mean (±SD) TIR 3.9-10.0 mmol/L (70-180 mg/dL) [empagliflozin group, 61.7±17.1%; semaglutide group, 70.6±14.7%; control group, 69.7±17.4%]; mean (±SD) CV of glucose | |

| |
|---|
| [empagliflozin group, 33.8±5.7%; semaglutide group, 35.3±6.1%; control group, 33.9±5.5%]; mean (±SD) LDL cholesterol [empagliflozin group, 2.61±0.86 mmol/L; semaglutide group, 2.66±0.71 mmol/L; control group, 2.62±0.78 mmol/L]; mean (±SD) HDL cholesterol [empagliflozin group, 1.56±0.36 mmol/L; semaglutide group, 1.53±0.38 mmol/L; control group, 1.51±0.39 mmol/L]; mean (±SD) triglycerides [empagliflozin group, 1.07±0.51 mmol/L; semaglutide group, 1.23±0.75 mmol/L; control group, |

| | | | |
|---|---|---|---|
| | 1.11±0.56 mmol/L]; number and percentage of smokers [empagliflozin group, 4 (13.3%); semaglutide group, 5 (16.7%); control group, 5 (17.2%)]; estimated glomerular filtration rate (eGFR) based on cystatin C (eGFRcys) [empagliflozin group, 86.1±9.2 mL/min/1.73 m$^2$; semaglutide group, 85.8±8.3 mL/min/1.73 m$^2$; control group, 86.5±7.8 mL/min/1.73 m$^2$]. | | |
| Retrospective chart review study (Mohandas et al. 2023 - Ref. 111) | • 54 adults with long-standing T1D who had been on long-acting GLP-1 RAs (including semaglutide, exenatide extended-release, dulaglutide, albiglutide) | • Baseline and post-GLP-1 RA initiation values were calculated for each parameter by averaging values over a 2-year period before starting the GLP-1 RAs and values collected | • Mean (±SD) HbA1c values decreased significantly from a baseline of 7.76±1.40% to 7.05±1.00% (mean difference = -0.71% percentage-points [%-points]; p=0.002; n=43).<br>• Mean (±SD) body weight decreased significantly from a baseline of 86.66±19.24 kg to 83.50±20.83 kg |

| | | | |
|---|---|---|---|
| | for at least 6 months.<br>• The study included data from 2 years before GLP-1 RA therapy initiation, and data from 6 or more months after GLP-1 RA therapy initiation.<br>• Inclusion criteria: patient age ≥18 years; diagnosis of T1D; use of a long-acting GLP-1 RA for ≥6 months.<br>• Exclusion criteria: age less than 18 years; pregnancy; history of concomitant steroid use.<br>• Baseline characteristics of the study participants: mean (±SD) age, 41.54±13.89 years; mean (±SD) diabetes duration, 16.37±12.92 years; 35 patients (64.8%) were fe- | starting from 6 months after the initiation of GLP-1 RA therapy (and ending at the time of chart review).<br>• Mean (±SD) duration of GLP-1 RA therapy: 23.85±15.46 months.<br>• The majority of patients (n=34; 63.0%) were using once-weekly subcutaneous semaglutide as GLP-1 RA.<br>• The remaining patients were using dulaglutide (n=19; 35.2%), exenatide extended-release (n=2; 3.7%) and albiglutide (n=2; 3.7%).<br>• Specific data regarding the medication dose and the changes in body weight and markers of glucose homeostasis | (mean difference = -3.16 kg; p=0.007; n=36).<br>• Mean (±SD) TIR increased significantly from a baseline of 54.59±23.12% to 66.74±17.82% (mean difference = +12.15% points; p=0.0009; n=23).<br>• Mean (±SD) TAR decreased significantly from a baseline of 42.20±21.47% to 30.23±15.66% (mean difference = -11.97% points; p=0.006; n=20).<br>• Mean (+SD) SD of glucose decreased significantly from a baseline of 56.09±18.26 mg/dL to 47.64±12.62 mg/dL (mean difference = -8.45% points; p=0.006665; n=17).<br>• Mean (±SD) 14-day CGM glucose values decreased significantly from a baseline of 182±32.00 mg/dL to 163±24.9 mg/dL (mean difference = -19 mg/dL; p=0.015; n=27).<br>• Mean (±SD) TBR decreased, although not significantly, from a baseline of 2.32±2.63% to 1.78±2.15% (mean difference = -0.54% points; p=0.0900; n=28).<br>• There was a non-significant |

| | | | | |
|---|---|---|---|---|
| | | male; 31 patients (57.4%) were White and 11 patients (20.3%) self-identified as Black or Hispanic/Latino.<br>• At the time of GLP-1 RA therapy initiation, 16 patients (29.6%) were using MDI insulin therapy, 36 (66.7%) were on insulin pump therapy, and 42 (77.8%) were using a CGM sensor.<br>• The majority of patients (n=28; 51.6%) were using the CGM sensor and the insulin pump as a closed-loop system; fewer patients (n=18; 33.3%) were using a CGM sensor plus MDI insulin therapy. | in semaglutide-treated group were not available. | decrease in mean (±SD) daily insulin requirements, from 0.553±0.22 units/kg/day to 0.547±0.19 units/kg/day (mean difference = -0.0061; p=0.83; n=18).<br>• There was no statistically significant difference between baseline and post-GLP-1 RA initiation values of serum creatinine, total cholesterol, LDL cholesterol, systolic blood pressure and diastolic blood pressure.<br><br>Side effects/adverse reactions:<br><br>• 15 patients (27.8%) discontinued GLP-1 RA therapy over a 2-year period.<br>• The most common reasons for GLP-1 RA discontinuation were gastrointestinal side effects such as nausea and vomiting (40.0%), minimal or negative impact of GLP-1 RAs on glucose control (20.0%), and lack of insurance coverage (6.7%).<br>• The remaining 33.3% of patients discontinued GLP-1 RAs for unknown reasons.<br>• There was no difference in the occurrence of |

| | | | |
|---|---|---|---|
| | | | hypoglycemia or DKA after the initiation of GLP-1 RA therapy, even considering that many participants (n=23 out of 36 for which C-peptide values were available; 63.9%) had C-peptide values lower than 0.2 nmol/L. |
| Retrospective study (Dandona et al. 2023 - Ref. 112) | • 10 patients (5 females and 5 males) with new-onset T1D.<br>• Baseline characteristics of the study participants: days since diagnosis of T1D [mean±SD], 33±19 days; age range, 21-39 years; mean (±SD) age, 27.0±6.3 years; mean (±SD) body weight, 78.4±8.3 kg; mean (±SD) BMI, 25.1±1.6 kg/m$^2$; mean (±SD) HbA1c, 11.7±2.1%; mean (±SD) fasting C-peptide, 0.65±0.33 ng/mL; mean (±SD) TDD of insulin, 0.35±0.11 | • Subcutaneous semaglutide (in addition to dietary carbohydrate restriction and standard basal and prandial insulin) was started at a weekly dose of 0.125 mg to avoid hypoglycemia and monitor other side effects.<br>• The weekly semaglutide dose was progressively titrated up to a maximum of 0.5 mg, while the dose of prandial insulin was gradually adjusted down.<br>• The basal insulin dose was reduced | • Semaglutide treatment led to the withdrawal of prandial insulin in all patients (within 3 months) and to the withdrawal of basal insulin in the majority of patients (in 7 out of 10 patients; within 6 months).<br>• Mean (±SD) HbA1c fell to 5.9±0.3% and 5.7±0.4% at 6 months and at 12 months, respectively.<br>• At 12 months, mean (±SD) fasting C-peptide level increased to 1.05±0.40 ng/mL, while the mean (±SD) TIR extrapolated from CGM was 89±3%.<br><u>Side effects/adverse reactions</u>:<br>• Mild hypoglycemia was reported only during the period of |

| | | | |
|---|---|---|---|
| | U/kg; mean (±SD) daily basal insulin dose, 0.21±0.12 U/kg; mean (±SD) daily prandial insulin dose, 0.15±0.09 U/kg.<br>• At the time of T1D diagnosis, 4 out of 10 patients presented with DKA, while the remaining patients presented with weight loss, polyuria and polydipsia.<br>• Nine patients had antibodies against glutamic acid decarboxylase, while one patient had autoantibodies against islet antigen 2. | according to CGM-derived data.<br>• Duration of the observation period: 12 months. | semaglutide dose up-titration.<br>• After semaglutide dose stabilization, there were no hypoglycemic episodes, DKA or other serious side effects. |
| Case report (Raven et al. 2023 - Ref. 103) | • 36-year-old woman with a 27-year history of T1D, undetectable fasting circulating C-peptide levels, and overweight (BMI: 29.3 kg/m$^2$). | • 6-month therapy with once-weekly subcutaneous (sc) semaglutide.<br>• Semaglutide was initiated at a starting weekly dose | • At 6 months from the initiation of semaglutide therapy: body weight change, -16.2 kg; BMI change, -6.0 kg/m$^2$; systolic blood pressure change, -18 mmHg; diastolic blood pressure change, -4 mmHg; HbA1c change, - |

| | | | |
|---|---|---|---|
| | • Treatment regimen at baseline: MDI insulin therapy [insulin glargine 300 units/mL 21 U daily plus insulin lispro 16 U daily] plus metformin (1000 mg twice daily) and oral contraceptive pill for polycystic ovary syndrome (PCOS). | of 0.25 mg, which was then up-titrated to 0.5 mg/week. | 0.9%; total daily insulin dose change, -16 units (insulin lispro: -10 units; insulin glargine: -6 units).<br><br><u>Side effects/adverse reactions</u>:<br>• Semaglutide (in addition to insulin and metformin) was well-tolerated by the patient.<br>• Semaglutide therapy was associated with reduced appetite, without significant nausea or gastrointestinal side effects, and without increase in hypoglycemia. |
| Case reports (Wong et al. 2023 - Ref. 121) | • Case 1: 32-year-old man with T1D (treated with an insulin pump), microalbuminuria, obstructive sleep apnea (OSA), restless legs syndrome, and class 3 obesity (body weight, 129.3 kg; BMI, 47.43 kg/m$^2$; body fat percentage, 63.6%); HbA1c value at presentation: 7.8%. | • Subcutaneous semaglutide and pramlintide combination therapy for 6 to 10 months. | <u>Case 1</u>:<br>• The patient was prescribed once-weekly subcutaneous semaglutide (dose: 0.25 mg/week, increased to 1 mg/week over a 2-month period) and pramlintide (15 mcg pre-meal sc injections, 3 times a day).<br>• At 10 months from the initial visit, the patient experienced a body weight loss of 20.9 kg (body weight: 108.4 kg; -16.1% of total body weight as compared to baseline) and a 15.2% reduction in body fat percentage. |

| | | |
|---|---|---|
| | • Case 2: this case was not considered, since the GLP-1 RA used was dulaglutide (out of the scope of this review).<br><br>• Case 3: a 49-year-old woman with T1D (treated with an insulin pump), hypertension, hypothyroidism, depression, and class 1 obesity (body weight, 81.7 kg; BMI, 30.9 kg/m$^2$; body fat percentage, 42.3%); HbA1c value at presentation: 7.9%; average TDD of insulin at presentation: 43 units/day. | • There was a reduction in insulin requirements, with no increase observed in the incidence of hypoglycemic events.<br>• The most recent HbA1c value was 7.6% (-0.2% as compared to baseline).<br>• LDL cholesterol values remained unchanged over the 10-month period.<br>• The patient showed a good semaglutide tolerability and tolerable nausea on pramlintide.<br><br><u>Case 3</u>:<br>• The patient was prescribed once-weekly subcutaneous semaglutide (dose: 0.5 mg/week) and pramlintide (dose: 15 mcg pre-meal sc injections, 3 times a day); after 6 months, pramlintide dose was increased to 30 mcg pre-meal sc injections (3 times a day).<br>• Over a 6-month period, the patient experienced a body weight loss of 14.6 kg (body weight: 67.1 kg; -17.9% of total body weight as compared to baseline) and a 5.0% reduction in body fat percentage; |

| | | | |
|---|---|---|---|
| | | | HbA1c value decreased to 7.0% (-0.9%); basal insulin requirements decreased from 23 units/day to 18 units/day; LDL cholesterol values remained unchanged over the 6-month period.<br>• The patient did not experience side effects (including hypoglycemic episodes) with GLP-1 RA and pramlintide combination therapy. |
| Retrospective case series study (Grassi et al. 2024 - Ref. 115) | • 11 patients (9 females and 2 males) older than 18 years with T1D and excess body weight (overweight or obesity), who were on PLGM (predictive low-glucose management) with sensor-augmented insulin pump therapy and had adhered to structured lifestyle modifications and education for weight management. | • 6-month low-dose once-weekly subcutaneous semaglutide (0.5 mg/week; the weekly semaglutide dose was titrated starting from lower doses). | • Mean (±SD) body weight at baseline (T0) was 82.8±10.9 kg and decreased to 76.9±10.6 kg at T3 and to 74.0±11.9 kg at T6 [-5.9 and -2.9 kg; p=0.003 and p=0.037, respectively], corresponding to an absolute reduction of 8.8 kg (-10.6%) from T0 to T6.<br>• At T6, as compared to T0, the proportion of patients with a BMI value >30 kg/m$^2$ significantly decreased from 6/11 to 0/11 (p=0.0124), with two subjects reaching a BMI value <25 kg/m$^2$.<br>• There was a significant reduction in mean (±SD) carbohydrate intake |

| | | | |
|---|---|---|---|
| | • Only patients on sensor-augmented insulin pump therapy were included in the study, in order to obtain structured CGM data access and detailed information on carbohydrate intake and insulin doses.<br><br>• Exclusion criteria: semaglutide use for less than 6 months; patients on MDI insulin therapy; lack of CGM data; history of bariatric surgery.<br><br>• Clinical and 30-day CGM data were extracted before the initiation of semaglutide therapy (T0), and at 3 (T3) and 6 months (T6) after the initiation of semaglutide therapy.<br><br>• Baseline clinical and | | from 137±52 g/day to 94±38 g/day from T0 to T3 (p=0.003), with a slight significant mean (±SD) carbohydrate intake increase to 109±45 g/day at T6 (p=0.01).<br>• The mean (±SD) number of daily meals decreased from 4.5±1.3 at T0 to 3.7±1.0 at T3 (p=0.011), without significant changes observed at T6.<br>• The mean (±SD) TDD of insulin significantly decreased from T0 to T3 (from 45.6±16.8 U/day to 38.7±15.9 U/day; p=0.006) and remained stable at T6 (38.5±16.5 U/day; p=0.929).<br>• No significant change was observed in mean (±SD) basal insulin dose (expressed in U/kg/day) from T0 to T3 and from T3 to T6, whereas mean (±SD) prandial insulin dose significantly decreased from T0 to T3 (from 0.28±0.12 U/kg/day to 0.22±0.10 U/kg/day; p=0.006), with a slight significant increase observed from T3 to T6 (from 0.22±0.10 U/kg/day to 0.24±0.10 U/kg/day; p=0.017). |

| | | |
|---|---|---|
| | demographic characteristics of the study participants: mean (±SD) age, 35.4±9.9 years; mean (±SD) diabetes duration, 19.7±6.7 years; mean (±SD) weight, 82.8±10.9 kg; mean (±SD) BMI, 30.9±3.8 kg/m$^2$; mean (±SD) TIR 70-180 mg/dL, 73.0±11.3%; mean TAR 180-250 mg/dL, 20.4% (SD not shown); mean TAR >250 mg/dL, 4.5% (SD not shown); mean TBR 54-69 mg/dL, 1.9% (SD not shown); mean TBR <54 mg/dL, 0.5% (SD not shown). | • CGM metrics (TIR, TBR and TAR) remained statistically unchanged throughout the 6-month observation period (SD for each mean value was not shown): mean TIR 70-180 mg/dL at 3 and 6 months, 73.4% and 73.3% (p=ns); mean TAR 180-250 mg/dL at 3 and 6 months, 19.4% and 20.1% (p=ns); mean TAR >250 mg/dL at 3 and 6 months, 3.8% and 3.9% (p=ns); mean TBR 54-69 mg/dL at 3 and 6 months, 2.3% and 2.1% (p=ns); mean TBR <54 mg/dL at 3 and 6 months, 0.6% and 0.6% (p=ns).<br><br>Side effects/adverse reactions:<br><br>• 4 out of 11 subjects experienced transitory nausea when initiating or up-titrating the semaglutide dose.<br>• No patients experienced vomiting or any other semaglutide-related side effect.<br>• TBR remained statistically unchanged throughout the 6-month observation period. |

| | | | |
|---|---|---|---|
| Retrospective case-control study (Garg et al. 2024 - Ref. 116) | • 50 semaglutide-treated patients (35 females and 15 males) and 50 computer-matched patients (for age, gender, weight, BMI, ethnicity and diabetes duration; 35 females and 15 males) who were not on semaglutide or any other GLP-1 RA or weight-loss medication.<br>• All patients were affected by long-standing T1D and comorbid overweight or obesity.<br>• All patients included in the study used a CGM device (with or without an insulin pump) and remained on intensive insulin treatment (defined as MDI insulin therapy, insulin pump | • Non-insulin adjunct therapy with once-weekly subcutaneous semaglutide for at least 3 months.<br>• Duration of the observation period: 12 months.<br>• Semaglutide dose was gradually titrated during the first 3 months to induce tolerability.<br>• The mean (±SD) weekly dose of semaglutide dose was 0.63±0.46 mg at 3 months, which increased to 0.78±0.48 mg, 0.86±0.30 mg, and 0.92±0.28 mg at 6, 9, and 12 months, respectively.<br>• Only 4 patients used semaglutide at a dose of 1.7 mg/week intermittently (due | • As compared to controls, semaglutide-treated patients experienced significantly greater reductions in body weight and BMI values, as well as significantly greater percent body weight loss.<br><u>Group comparison at 12 months</u>:<br>• Semaglutide-treated patients experienced a significant mean body weight loss of 15.9 lbs (-7.6% of the baseline body weight and -7.9% of the baseline BMI), while controls showed a mean body weight gain of 2.1 lbs (+1.1% of the baseline body weight).<br>• Mean (±SD) BMI change at 12 months: semaglutide group, -2.65±0.85 kg/m$^2$ [-7.9±2.6%]; control group, +0.12±0.78 kg/m$^2$ [+0.8±2.4%]; p-values regarding comparison of absolute and percent values between the two groups: <0.0001 and ≤0.0001, respectively.<br>• Mean (±SD) BMI change (overall change): |

| | | | |
|---|---|---|---|
| | therapy, or use of a hybrid closed-loop system) throughout the study duration.<br><br>• Inclusion criteria: 1) age between 18 and 80 years; 2) use of semaglutide for at least 3 months; 3) use of a CGM device.<br><br>• Exclusion criteria included: 1) patients prescribed any other GLP-1 receptor agonist (lixisenatide, dulaglutide, exenatide, and liraglutide) or other weight-loss medications; 2) T2D; 3) use of semaglutide for less than 3 months; 4) pregnancy.<br><br><u>Baseline characteristics of the study participants</u>: | to the lack of insurance coverage). | semaglutide group, -1.44±0.79 kg/m$^2$ [-4.2±2.4%]; control group, -0.20±0.75 kg/m$^2$ [-0.33±2.3%]; p-values regarding comparison of absolute and percent values between the two groups: 0.0029 and 0.0029, respectively.<br><br>• Mean (±SD) body weight change at 12 months: semaglutide group, -15.9±5.4 lbs [-7.6±2.6%]; control group, +2.1±5.0 lbs [+1.1±2.4%]; p-values regarding comparison of absolute and percent values between the two groups: <0.0001 and <0.0001, respectively.<br><br>• Mean (±SD) body weight change (overall change): semaglutide group, -8.4±5.1 lbs [-4.0±2.4%]; control group, +0.53±0.48 lbs [+0.2±2.3%]; p-values regarding comparison of absolute and percent values between the two groups: 0.0009 and 0.0012, respectively.<br><br>• Overall change in mean (±SD) HbA1c values: semaglutide |

| | | | |
|---|---|---|---|
| | • Most participants were non-Hispanic White (n=46; 92%; in both arms).<br>• Mean (±SD) diabetes duration: 27±13 years (semaglutide group) and 27±12 years (control group); mean (±SD) age, 42±11 years (semaglutide group) and 42±11 years (control group); mean (±SD) weight, 213±37 lbs (96.9±16.8 kg) [semaglutide group] and 208±42 lbs (94.5±19.1 kg) [control group]; mean (±SD) BMI, 33.5±5.8 kg/m$^2$ (semaglutide group) and 32.7±5.8 kg/m$^2$ (control group); mean (±SD) HbA1c, 7.6±1.2% (semaglutide group) and | | group, -0.60±0.11%; control group, -0.17±0.11%; p=0.0046.<br><br>• In semaglutide-treated subjects, TDD of insulin was higher at baseline and remained higher during the entire study period.<br><br>• There was no group difference in terms of overall changes in insulin doses.<br>• There was no group difference in the basal or prandial insulin dose during the entire study period.<br><br>• The semaglutide group, as compared to the control group, showed significantly greater overall increases in mean (±SD) TIR values [+6.2±1.8% vs. +0.7±1.9%; p-value: 0.0351], as well as significantly greater overall reductions in measures of glycemic variability, namely: mean (±SD) CGM SD of glucose, -5.9±1.5 mg/dL vs. -0.8±1.7 mg/dL (p-value: 0.0232); mean (±SD) CV of glucose, -1.4±0.6% vs. |

| | | | |
|---|---|---|---|
| | 8.2±1.7% (control group); mean (±SD) TDD of insulin, 70.3±28.2 U/day (0.71±0.23 U/kg/day) [semaglutide group] and 57.3±26.4 U/day (0.59±0.23 U/kg/day) [control group].<br><br>• There were no significant differences between the semaglutide group and the control group in baseline HbA1c, body weight, BMI, CGM metrics, and use of diabetes technology.<br><br>• The only characteristic that was significantly different between the semaglutide group and the control group at baseline was the TDD of insulin: as compared to | | -0.8±1.7% (p-value: 0.0340).<br><br>• No significant group difference was observed for overall changes in mean (±SD) TAR and TBR values.<br><br><u>Side effects/adverse reactions</u>:<br><br>• None of the study participants reported any episode of severe hypoglycemia or DKA that required hospitalization. |

| | | | |
|---|---|---|---|
| | subjects in the control group, semaglutide-treated subjects showed a significantly higher TDD of insulin [expressed as both total units/day (p=0.0254) and units per kg body weight/day (p=0.0165)]. | | |
| Case report (Gad and Malik; 2024 - Ref. 122) | • 34-year-old woman with a 23-year history of T1D (long-standing T1D), who presented with weight gain (about 10 kg) over 3 to 4 years, overweight (body weight: 63 kg; BMI: 26.9 kg/m$^2$), an HbA1c value of 8.3%, and increased glycemic variability (CV of glucose: 48.2%).<br>• Treatment regimen at the time of presentation: MDI insulin | • Once-weekly subcutaneous semaglutide was prescribed at a starting dose of 0.25 mg/week for the initial 2 weeks.<br>• The weekly dose of semaglutide was then increased to 0.5 mg for the subsequent 2 weeks; afterwards, the weekly dose of semaglutide was titrated to a maintenance dose of 1.0 mg. | • Semaglutide therapy for 2 months resulted in a body weight reduction of 12 kg (body weight: 51 kg), a percent body fat reduction of 15%, and a visceral fat reduction of 7%.<br>• After 2 months of semaglutide therapy, TAR >250 mg/dL was reduced from 11% to 4%, although this reduction was accompanied by a slight increase of TAR 181-250 mg/dL (from 19% to 22%) and occurred partly at the expense of increases in TBR 54-69 mg/dL (from 8% to 10%) and TBR <54 mg/dL (from 3% to 5%). |

| | | | |
|---|---|---|---|
| | therapy based on the use of basal insulin glargine U300 (at a daily dose of 14 U) plus prandial insulin lispro 100 units/mL (the latter administered at each meal at a dose based on the carbohydrate counting, insulin-to-carbohydrate ratio and insulin sensitivity factor); the patient was using the FreeStyle Libre 2 glucose sensor, and had no evidence of microalbuminuria, retinopathy or neuropathy. | | • TIR 70-180 mg/dL remained unchanged after 2 months of semaglutide therapy (59%).<br>• Glucose Management Indicator (GMI), average glucose and CV of glucose decreased from 6.9% to 6.6%, from 150 mg/dL to 139 mg/dL, and from 48.2% to 44.6%, respectively.<br>• There was a reduction in the doses of insulin glargine (-2 U) and insulin lispro (about -2 U based on carbohydrate counting).<br><br><u>Side effects/adverse reactions</u>:<br><br>• 2-month semaglutide therapy was well-tolerated by the patient. |
| Case report (Gong and Wentworth; 2024 - Ref. 123) | • 27-year-old woman with obesity and long-standing T1D, who was treated with basal-bolus insulin therapy starting from the disease diagnosis (at 10 years of age). | • 11-month intermittent subcutaneous semaglutide therapy.<br><br>• Once-weekly subcutaneous semaglutide therapy was started for the treatment of | • After one month of semaglutide therapy (at a weekly dose of 0.25 mg), the patient's body weight had already decreased to 77 kg (-5 kg) and TIR 3.9-10.0 mmol/L had increased from 9% to 44%.<br>• GMI decreased from 11.4% to |

| | | | |
|---|---|---|---|
| | • Patient's comorbidities: anxiety (treated with monthly psychological counselling in combination with vortioxetine therapy); severe binge eating disorder.<br><br>• Baseline characteristics of the study participants: body weight, 82 kg; BMI, 30.1 kg/m$^2$; HbA1c, 12.9% (117 mmol/mol); GMI, 11.4%; fasting C-peptide, <0.03 nmol/L; fasting blood glucose, 15.2 mmol/L (273 mg/dL); TAR >13.9 mmol/L, 77%; TAR 10.1-13.9 mmol/L, 14%; TIR 3.9-10.0 mmol/L, 9%; TBR 3.0-3.8 mmol/L, 0%; TBR <3.0 mmol/L, 0%.<br><br>• CGM report showed severe | obesity at an initial weekly dose of 0.25 mg, which was then increased to 0.5 mg after 4 weeks.<br><br>• Semaglutide therapy was interrupted after 2 months due to drug shortage.<br><br>• During the subsequent 8 months, the patient used semaglutide therapy intermittently, at an average weekly dose of 0.5 mg. | 7.7%, BMI decreased from 30.1 kg/m$^2$ to 28.4 kg/m$^2$, and TDD of insulin decreased from 97 units to 79 units.<br>• TAR >13.9 mmol/L was 19%, TAR 10.1-13.9 mmol/L was 34%, TIR 3.9-10.0 mmol/L was 44%, TBR 3.0-3.8 mmol/L was 3%, and TBR <3.0 mmol/L was 0%.<br>• There was a reduction in the BES score (from 39 to 3) and in the DASS-21 score (Depression-Anxiety-Stress scales: from 18-10-17 to 6-9-12).<br><br>• After 2 months, the patient had to stop semaglutide therapy due to drug shortage. One month later, she experienced recurrent symptoms of binge eating disorder, weight regain (body weight: 84 kg; BMI: 30.8 kg/m$^2$) and deterioration of glucose control (GMI: 10.2%).<br><br>• During the subsequent 8 months, the patient obtained a sporadic supply of semaglutide and used the medication at |

| | | | |
|---|---|---|---|
| | hyperglycemia that worsened during overnight binges.<br>• The patient scored 39/46 on the Binge Eating Scale (BES) and reported significant weight-related distress contributing to high depression scores on the 21-item Depression Anxiety and Stress Scale (DASS-21). | | an average weekly dose of 0.5 mg.<br>• Despite the intermittent use of semaglutide, the patient experienced remission of binge eating disorder symptoms and improvement of depression, stress and anxiety symptoms.<br>• At 11 months from the initiation of semaglutide therapy, body weight was 69 kg (-13 kg, as compared to baseline), BMI was 25.3 $kg/m^2$, TDD of insulin was 65 units, GMI was 8.6%, HbA1c was 9.9%, TAR >13.9 mmol/L was 35%, TAR 10.1-13.9 mmol/L was 27%, TIR 3.9-10.0 mmol/L was 36%, TBR 3.0-3.8 mmol/L was 2%, TBR <3.0 mmol/L was 0%, BES score was 4, and the DASS-21 score was 5-8-15 (Depression-Anxiety-Stress scales). |
| Retrospective study (Almohareb et al. 2024 - Ref. 117) | • Multicenter retrospective study aimed to assess the safety and efficacy of GLP-1 RAs in T1D patients based on real-world data. | • The majority of patients (n=92; 63.9%) were using semaglutide (once-weekly subcutaneous semaglutide); most of | • Among patients who underwent a follow-up visit within 4-6 months from GLP-1 RA therapy initiation (n=74 patients), mean (±SD) HbA1c value significantly declined from 9.0±1.5% (at baseline) to |

| | | | |
|---|---|---|---|
| | • The study included T1D patients older than 16 years with a confirmed diagnosis of T1DM, who started GLP-1 RA therapy as an add-on treatment to insulin for better diabetes management or for weight loss.<br><br>• Patients excluded from the study: patients with T2D or other types of diabetes mellitus, as well as those for which there were no available data regarding baseline laboratory parameters or follow-up visits after the initiation of GLP-1 RA therapy.<br><br>• The study included 144 T1D patients (n=93 females; 64.6%) who started GLP-1 RA therapy as an | these patients (n=81; 88.0%) used 0.25 mg as the starting weekly semaglutide dose, and 1 mg as the maintenance weekly semaglutide dose (n=74; 80.4%).<br><br>• Of the remaining 52 patients, 49 were using liraglutide and 3 were using dulaglutide.<br><br>• About one-fourth of semaglutide-treated patients decided to discontinue the medication (n=21; 22.8%) before the end of the 18-month follow-up period.<br><br>• Mean (±SD) semaglutide treatment duration: 7.9±3.7 months.<br><br>• Duration of the observation period: | 8.4±1.3% (mean±SD reduction: -0.5±1.0%; p=0.0004), and this was accompanied by significant mean (±SD) reductions in the values of body weight (-3.6±5.0 kg; p<0.0001), BMI (-1.4±1.9 kg/m$^2$; p<0.0001) and daily basal insulin dose (-2.4±9.2 units/day; p=0.0282).<br><br>• Among patients who underwent a follow-up visit within 12-18 months (n=38 patients) from GLP-1 RA therapy initiation, there was a significant mean (±SD) reduction in HbA1c value from baseline of 0.5±0.8% (p=0.0022), and this was accompanied by significant mean (±SD) reductions in the values of body weight (-5.2±6.3 kg; p<0.0001), BMI (-1.9±2.3 kg/m$^2$; p<0.0001) and daily basal insulin dose (-4.1±9.2 units; p=0.0101).<br><br>Side effects/adverse reactions:<br><br>• Only 3 patients (3.2%) in the semaglutide group discontinued the drug due to |

| | | | |
|---|---|---|---|
| | add-on treatment to insulin.<br><br>• Baseline characteristics of the study participants: mean (±SD) age, 33.0±10.1 years; mean (±SD) diabetes duration, 16.5±7.8 years; mean (±SD) body weight, 89.9±16.3 kg; mean (±SD) BMI, 34.0±5.7 kg/m$^2$; mean (±SD) HbA1c, 8.9±1.6%; mean (±SD) fasting blood glucose, 214.5±88.8 mg/dL; mean (±SD) TDD of insulin, 81.2±33.2 units/day (corresponding to 0.9±0.4 units/kg/day); the majority of patients (n=133; 92.4%) were using MDI insulin therapy, whereas only 11 patients (7.6%) | patients were followed until GLP-1 RA therapy was discontinued or a GLP-1 RA was replaced with another medication of the same class, or until 18 months after the initiation of GLP-1 RA therapy.<br><br>• Specific information on changes in HbAc, body weight, BMI and TDD of insulin regarding the semaglutide-treated group was not available. | gastrointestinal side effects (nausea, vomiting or diarrhea), which represented the most frequently cited reasons for GLP-1 RA discontinuation in this subset of patients.<br>• 5 patients in the semaglutide group discontinued the drug for different reasons (two for being pregnant or for planning to become pregnant, one for a reported unspecified intolerance, one for medication noncompliance, and one for the replacement of semaglutide with another GLP-1 RA).<br>• GLP-1 RA therapy did not result in a higher occurrence of severe hypoglycemia or DKA.<br>• During the follow-up period, only one patient had a severe hypoglycemic event (requiring access to the hospital), although it was not specified whether this patient was on semaglutide therapy or on other GLP-1 RAs.<br>• Minor hypoglycemic events (not requiring access to the hospital) were reported by 18 patients (12.5%) during the follow-up |

| | | | |
|---|---|---|---|
| | were on insulin pump therapy; there was a limited number of patients using CGM systems (n=27; 18.8%). | | period, although it was not specified how many of these patients were on semaglutide therapy or on other GLP-1 RAs. |
| Retrospective study (Orrange et al. 2024 - Ref. 118) | • Retrospective chart review of 23 patients with T1D, 86% of whom were affected by overweight or obesity.<br>• The study aimed to evaluate the impact of once-weekly subcutaneous semaglutide on glucose control and weight loss in this population.<br>• Baseline characteristics of the study participants: mean age, 45 years; mean diabetes duration, 25 years; 78% of participants used CGM devices, while | • Study participants were on once-weekly subcutaneous semaglutide for at least 6 months.<br>• Participants received primarily telemedicine-based care during and after the Coronavirus disease 2019 (COVID-19) pandemic (this period limited HbA1c data capture).<br>• Duration of the follow-up period: 7 to 50 months. | • Semaglutide therapy determined a significant weight loss, with patients losing an average of 5% of their baseline body weight (approximately 3.75 kg) during a 22-month follow-up period.<br>• At baseline, 43% of participants were classified as affected by obesity (BMI ≥30 kg/m$^2$).<br>• At the end of the follow-up period (after 9+ months), 2 participants had transitioned from obesity to overweight, while one participant had transitioned from overweight to normal weight.<br>• The majority of participants achieved weight loss with lower weekly semaglutide doses: 6 participants on 0.25 mg, 5 participants on 0.5 mg, 8 participants on 1.0 mg, and only 4 |

| | | | |
|---|---|---|---|
| | 61% of participants used automated insulin delivery (AID) systems.<br>• The study cohort was predominantly composed of non-Hispanic White (91%) and female (83%) participants. | | participants on 2.0 mg.<br>• Semaglutide did not lead to significant improvements in glucose control, as evidenced by the lack of statistically significant changes in GMI and TIR.<br>• TDD of insulin remained relatively unchanged, decreasing slightly from a mean (±SD) value of 47.7±18 units/day (0.56 units/kg/day) at baseline to a mean (±SD) value of 46.9±21.7 units/day (0.61 units/kg/day) at the end of the observation period, with no significant changes being observed during follow-up. |
| Case series (Seetharaman and Cengiz; 2024 - Ref. 139) | • Case series including adolescents and young adults (AYA) with T1D who received GLP-1 RA therapy (as an adjunct therapy to insulin).<br>• Patients were selected based on clinical needs, | • Case series of 8 AYA with T1D who received once-weekly subcutaneous semaglutide or tirzepatide at different doses and for different periods of time. | • Most patients in this case series showed notable improvements in HbA1c (up to -2.2%), TIR (up to +27%), average glucose levels (up to -50 mg/dL), reductions in TDD of insulin (up to -0.67 U/kg/day) and mild-to-moderate weight loss (up to 20.5 kg; except for one patient) after treatment with semaglutide or |

| | | | |
|---|---|---|---|
| | particularly concurrent obesity or suboptimal glucose control.<br><br>• The GLP-1 RAs used in this case series included semaglutide and tirzepatide.<br><br>• This case series included 8 patients. 7 out of the 8 patients had obesity.<br><br>• The remaining patient was an 18-year-old female with T1D for 5 years (managed with Omnipod insulin pump and Dexcom CGM sensor), who had a BMI of 22.9 kg/m$^2$ (body weight: 60.9 kg; 134 lbs) and was prescribed once-weekly subcutaneous semaglutide (at a starting dose of 0.25 mg/week) as | | tirzepatide (used for up to 16 months).<br><br><u>Side effects/adverse reactions</u>:<br><br>• A significant proportion of patients experienced common gastrointestinal side effects of GLP-1 RAs, such as nausea, vomiting and diarrhea, even when these medications were used at low doses.<br>• Gastrointestinal side effects generally improved after a few weeks, with some patients managing them with the use of histamine H2-receptor antagonists and/or antiemetic drugs. Gradual GLP-1 RA dose titration helped minimize these common gastrointestinal side effects.<br>• One patient (a 14-year-old female with a 3-year history of T1D, managed with an Omnipod insulin pump and with a Dexcom CGM sensor) experienced an increase in hypoglycemic episodes, thus requiring a decrease in her daily insulin dose.<br>• There were no reported episodes of DKA. |

| | | | |
|---|---|---|---|
| | an adjunct therapy for postprandial hyperglycemia and appetite regulation. | | • No patients exhibited depression, suicidal thoughts or mood issues. |
| Retrospective study (Cohen et al. 2025 - Ref. 119) | • Ethics committee (Alfred Hospital, Melbourne, Australia) retrospective audit of a tertiary referral clinic to evaluate real-world outcomes in adult patients with T1D who were using adjunctive therapy with GLP-1 RA for two or more visits.<br>• Electronic medical records (from the Baker Heart and Diabetes Institute, Melbourne, Australia) were audited from 2012 onwards, with follow-up ending before the national GLP-1 RA shortage (February 2022). | • Among 1480 adults (aged 18 years or over) with T1D, 30 subjects (2%) were prescribed semaglutide and had one or more follow-up visits while they were still using the medication.<br>• Mean (±SD) follow-up duration: 255±149 days (range: 41-501 days).<br>• Information regarding semaglutide formulations and dosages was not available. | • Mean body weight decreased significantly by 8.2 kg (8.4% body weight reduction; $p<0.0001$).<br>• Mean (±SD) HbA1c decreased by 0.5±1.2% (to 8.0±1.2%) [$p=0.011$].<br>• Five subjects achieved an HbA1c value <7.0% (<53 mmol/mol).<br>• There was a significant increase in mean eGDR by 0.05 (to 2.09±0.019) [$p=0.049$].<br>• Blood pressure values, serum lipid profile and CGM metrics did not change significantly.<br><u>Side effects/adverse reactions</u>:<br>• There were no reported cases of DKA. |

- Estimated glucose disposal rate (eGDR) was used as a biomarker for insulin sensitivity in T1D; eGDR was expressed as the natural log: elnGDR = 3.091 - 0.141 x HbA1c.

- Baseline characteristics of the study participants: mean (±SD) age, 55±13 years; male participants, 40%; mean (±SD) body weight, 97.6±21.8 kg; mean (±SD) BMI, 34.0±4.7 $kg/m^2$; mean (±SD) HbA1c, 8.5±1.1%; mean (±SD) systolic blood pressure, 131±19 mmHg; mean (±SD) diastolic blood pressure, 80±8 mmHg; mean (±SD) elnGDR, 2.04±0.16; all patients had

| | | | |
|---|---|---|---|
| | an HbA1c value >7.0% (>53 mmol/mol)<br><br>• Baseline and follow-up CGM data were available for only 12 subjects; baseline CGM metrics: mean (±SD) TIR, 53±13%; mean (±SD) TAR, 43±12%; mean (±SD) TBR, 4.2±3.2%; mean (±SD) CV of glucose, 38±6%. | | |
| Retrospective chart review (Snell-Bergeon et al. 2025 - Ref. 136) | • Retrospective chart review to assess the safety and efficacy of off-label semaglutide and tirzepatide use in adult patients with T1D over a period of 1 year.<br><br>• Inclusion criteria: age >18 years; prescribed semaglutide or tirzepatide for at least 3 months or frequency | • Patients were started on an initial weekly dose of 0.25 mg for semaglutide and of 2.5 mg for tirzepatide, as per clinical guidelines.<br><br>• The median weekly dose of semaglutide prescribed was 0.5 mg, while the median weekly dose of tirzepatide prescribed | • At 12 months, BMI decreased by a least square mean±standard error (SE) of 3.0±0.5 kg/m$^2$ and 7.5±0.5 kg/m$^2$ in the semaglutide and tirzepatide groups, respectively.<br>• There was no significant change in BMI in the control group at any time point.<br><br>• At 12 months, body weight decreased by a least square mean±SE of 19.2±3.0 lbs (9.1%) and 49.4±3.0 lbs (21.4%) in the semaglutide and tirzepatide groups, respectively. |

| | | | |
|---|---|---|---|
| | matched control not using weight-loss drugs; and use of intensive insulin therapy through MDI, insulin pumps or AID systems.<br><br>• Exclusion criteria: diagnosis of T2D; pregnancy during the study period; and use of any other weight-loss drug.<br><br>• 208 patients (86 using semaglutide, 122 using tirzepatide) were excluded from the study due to missing data and due to the fact that they did not meet inclusion or exclusion criteria.<br><br>• Many visits were conducted via telehealth during the COVID-19 | was 7.5 mg weekly.<br><br>• Two semaglutide users remained at the initial weekly dose of 0.25 mg, while the number of patients reaching the weekly semaglutide doses of 0.5 mg, 1 mg, and 2 mg was 38 (76%), 9 (18%), and 1 (2%), respectively.<br><br>• Two tirzepatide users remained on the initial weekly dose of 2.5 mg weekly, while the number of patients reaching the weekly tirzepatide doses of 5.0 mg, 7.5 mg, 10 mg, 12.5 mg, and 15 mg was 16 (32%), 8 (16%), 14 (28%), 5 (10%), and 5 (10%), respectively.<br><br>• After starting the | • BMI, weight in pounds and weight percentage decreased significantly more in the tirzepatide group than in the control group at all time points from 3 to 12 months, and significantly more in the semaglutide group than in the control group at 6, 9, and 12 months.<br><br>• The reduction in BMI, weight in pounds and weight percentage was significantly greater in the tirzepatide group than in the semaglutide group at all time points.<br><br>• At 12 months, 93% of tirzepatide users, 77% of semaglutide users and 14% of controls had lost 5% of their baseline body weight ($p < 0.0001$), while 87% of tirzepatide users, 47% of semaglutide users and none of the controls had lost 10% or more of their baseline body weight ($p < 0.0001$).<br><br>• At 12 months, there was a significant reduction in the least square mean±SE HbA1c of 0.54±0.14% in |

| | | | |
|---|---|---|---|
| | pandemic, which led to some missing HbA1c and body weight values.<br>• 12 patients who were prescribed tirzepatide had used semaglutide prior to starting tirzepatide and were already included in the semaglutide case cohort; these patients were removed from the tirzepatide cohort to avoid duplication of these participants in the analysis and in line with the inclusion criteria of “no prior weight-loss drug use”.<br>• The retrospective chart review included 100 patients who were prescribed semaglutide (n=50) or tirzepatide (n=50) and | medication, three semaglutide users and one tirzepatide user discontinued the drug due to lack of response.<br>• The majority of patients in all groups (82% of controls, 84% of semaglutide users, and 78% of tirzepatide users) had at least 6 months of available follow-up data.<br>• More than half of controls (58%) and semaglutide users (64%) had at least 9 months of available follow-up data, while just under half of tirzepatide users (48%) had at least 9 months of available follow-up data. | the semaglutide group, and a reduction in the least square mean±SE HbA1c of 0.68±0.16% in the tirzepatide group, with no change observed in HbA1c among controls (-0.04±0.13%).<br>• The reduction in HbA1c was significantly greater in the semaglutide group than in the control group (p=0.006) and in the tirzepatide group than in the control group (p <0.0001) overall.<br>• Although there was no difference in the amount of reduction in HbA1c between semaglutide group and tirzepatide group overall (p=0.175), there was a greater reduction in the tirzepatide group than in the semaglutide group at 6 months.<br>• In the tirzepatide group, TDD of insulin as well as basal and bolus insulin doses (expressed either in U/day or U/kg/day) decreased significantly from baseline and to a |

| | | | |
|---|---|---|---|
| | 50 controls frequency matched for age, sex, BMI, HbA1c and diabetes duration, and who did not receive any weight-loss drugs during the study period.<br>• Data were collected before the initiation of weight-loss drugs (baseline) and for up to 1 year for each patient.<br><br>• Baseline characteristics of the study participants: least square mean±standard error (SE) age, 41±2 years (control group) vs. 42±2 years (semaglutide group) vs. 39±2 years (tirzepatide group); gender (number of male participants), n=28 (14%) [control group] vs. n=30 (15%) [semaglutide | | greater extent than in the semaglutide and control groups at all time points (least square mean±SE TDD at 12 months: tirzepatide group, -26.4±3.8 U/day [-0.13±0.03 U/kg/day]; semaglutide group, -4.1±3.9 U/day [0.02±0.04 U/kg/day]; control group, 3.9±3.9 U/day [0.02±0.04 U/kg/day]; $p<0.05$).<br><br>• With regard to the comparison between semaglutide and control groups in terms of daily insulin doses, only bolus insulin dose at 6 months significantly differed between semaglutide users and controls (least square mean±SE bolus insulin dose at 6 months: semaglutide group, -4.2±2.7 U/day; control group, 3.6±2.3 U/day; $p<0.05$).<br><br>• Changes in BMI, body weight and HbA1c did not differ significantly between groups based on insulin delivery method (MDI insulin therapy, insulin pump/AID). |

| | | | |
|---|---|---|---|
| | group] vs. n=28 (14%) [tirzepatide group]; least square mean±SE diabetes duration: 27±2 years [control group] vs. 27±2 years [semaglutide group] vs. 24±2 years [tirzepatide group]; non-Hispanic White (number of participants): n=90 (45%) [control group] vs. n=90 (45%) [semaglutide group] vs. n=86 (43%) [tirzepatide group]; least square mean±SE BMI: 34.4±0.8 $kg/m^2$ (control group) vs. 33.4±0.8 $kg/m^2$ (semaglutide group) vs. 35.0±0.8 $kg/m^2$ (tirzepatide group); least square mean±SE body weight: 98.9±2.6 kg (control | | • Weight loss remained significantly greater in tirzepatide-treated patients than in semaglutide-treated patients, regardless of the insulin delivery method. |

| |
|---|
| group) vs. 96.7±2.6 kg (semaglutide group) vs. 103.7±2.6 kg (tirzepatide group); least square mean±SE HbA1c: 7.3±0.2% (control group) vs. 7.6±0.2% (semaglutide group) vs. 7.0±0.2% (tirzepatide group*) [*p<0.05 compared with semaglutide group]; least square mean±SE TDD of insulin: 61.6±4.6 U/day (control group) vs. 70.3±5.1 U/day (semaglutide group) vs. 76.5±4.8 U/day (tirzepatide group*) [*p<0.05 compared with the control group]; insulin pump users (number of participants): n=80 (40%) [control |

| | | | |
|---|---|---|---|
| | group] vs. n=64 (32%) [semaglutide group] vs. n=86 (43%) [tirzepatide group]; commercial health insurance (number of participants): n=88 (29%) [control group] vs. n=92 (46%) [semaglutide group] vs. n=94 (47%) [tirzepatide group].<br><br>• Baseline matching characteristics (age, sex, BMI, HbA1c and diabetes duration) were similar between the cases and controls, with no differences observed in matching variables between the cases and controls.<br><br>• Almost all study participants had commercial health insurance, and there were | | |

no group differences in terms of proportion of patients with commercial health insurance.

- At baseline, TDD of insulin and basal daily insulin dose were higher in the tirzepatide group than in the control group, while HbA1c was lower in the tirzepatide group than in the semaglutide group.

- Almost all patients who were prescribed semaglutide (n=36; 72%) and tirzepatide (n=43; 86%) were affected by obesity, while 11 (22%) semaglutide-treated patients and 7 (14%) tirzepatide-treated patients were

| | | | |
|---|---|---|---|
| | affected by overweight.<br><br>• All insulin pump users were using AID systems. | | |
| Randomized, double-blind, crossover trial (Pasqua et al. 2025 - Ref. 105) | • Randomized, double-blind, crossover trial conducted at the Research Institute of the McGill University Health Centre in Montreal (Quebec, Canada).<br><br>• Aim of the study: to evaluate whether once-weekly subcutaneous semaglutide, as compared to placebo, improves glucose control and other non-glycemic outcomes in adults with T1D while using AID.<br><br>• Inclusion criteria: age ≥18 years; diagnosis of T1D for 1 year or longer; use | • Each arm was 15 weeks (with 2 weeks of washout) and the total study duration was 32 weeks.<br>• After the initial visit, at each intervention, participants were titrated up to 1 mg or the maximum tolerated dose of once-weekly subcutaneous semaglutide or placebo over a 11-week dose titration period (0.25 mg weekly for 4 weeks; subsequently, 0.5 mg weekly for 4 weeks; then, 1 mg onward).<br><br>• Of the 28 participants who were randomized, 24 | • As compared to placebo, semaglutide significantly increased TIR, without increasing the TBR.<br>• For the last 28 days of each intervention, during the use of the study's AID system, mean (±SD) TIR 3.9-10.0 mmol/L (TIR 70-180 mg/dL) was 74.2±9.7% for semaglutide vs. 69.4±10.4% for placebo, a significant paired difference of 4.8±7.6 percentage points (p=0.006).<br><br>• During the last 28 days of AID use, there was no difference in mean values of TBR <3.9 mmol/L (TBR <70 mg/dL) (p=0.19) between the interventions.<br><br>• There was a significant difference between the interventions in mean (±SD) values of TAR >10.0 mmol/L (TAR >180 mg/dL): semaglutide, 24.1±10.1% |

| | | | |
|---|---|---|---|
| | of an insulin pump for 3 months or longer; HbA1c of 11% or less; and agreement to use a highly effective method of birth control in subjects of childbearing potential or active avoidance of pregnancy during the study (if applicable).<br>• Exclusion criteria: personal or family history of medullary thyroid cancer or multiple endocrine neoplasia type 2; current or recent (<2 weeks) use of an anti-hyperglycemic agent other than insulin; severe hypoglycemic episode within 3 months; DKA requiring medical attention and intravenous insulin within 6 months; | completed the trial.<br>• Four participants did not complete the trial: two participants because of their desire to use semaglutide therapy after the first intervention rather than continue the study (these participants had assumed they were on semaglutide during the first intervention); one participant because of difficulty complying with the study procedures; one participant because of the side effects related to semaglutide therapy at a weekly dose of 0.5 mg.<br>• Of the 24 participants who completed the trial, the maximum tolerated dose of semaglutide was 0.25 mg | vs. placebo, 29.1±10.6% (paired difference of 5.0±8.0%; p=0.006).<br>• There was a significant difference between the interventions in median values of TAR >13.9 mmol/L (TAR >250 mg/dL): semaglutide, 5.4% [IQR: 2.7, 8.9] vs. placebo, 8.4% [IQR: 4.0, 12.0] (paired difference of 1.7% [IQR: -4.2, 0]; p=0.008).<br>• Mean (±SD) glucose levels and SD of glucose levels were significantly reduced with semaglutide use (8.4±0.9 mmol/L and 3.0±0.6 mmol/L, respectively) as compared to placebo (8.8±1.0 mmol/L and 3.2±0.7 mmol/L, respectively) [p-value for mean glucose=0.02; p-value for SD of glucose=0.01], although the CV of glucose levels was not.<br>• There was a significant reduction with semaglutide, as compared to placebo, in median HbA1c and fructosamine values (placebo-adjusted |

| | | | |
|---|---|---|---|
| | history of acute or chronic pancreatitis or gallbladder disease; planned or ongoing pregnancy or breast-feeding; BMI of 21 kg/m² or less; bariatric surgery within 6 months; any prior adverse reaction to GLP-1 RAs; clinically significant diabetic retinopathy or gastroparesis; regular use of hydroxyurea; any serious medical or psychiatric illness that may interfere with trial participation; failure to comply with the study protocol or research group recommendations; inability to use AID system; inability or unwillingness to comply with safe diabetes | in 2 participants (8.3%), 0.5 mg in 6 participants (25.0%) and 1 mg in 16 participants (66.7%).<br>• The titration period was followed by the use of a research-based AID system for 4 weeks.<br>• The AID system included a Dexcom G6 CGM sensor, an Ypsomed pump, and a Pixel 2 smartphone with an application running the McGill insulin dosing algorithm.<br>• At the end of the 4-week AID use, anthropometric measurements and laboratory testing were performed.<br>• Participants returned to their usual insulin for a 2-week | change: HbA1c, -0.5% [IQR: -0.7, -0.2]; p<0.001; fructosamine, -15 μmol l⁻¹ [IQR: -39, 2]; p<0.001).<br>• The proportion of participants who achieved an HbA1c value <7% was significantly higher with semaglutide use as compared to placebo [semaglutide, n=17 (71%); placebo, n=10 (42%); p=0.002].<br>• Mean (±SD) HDL cholesterol value was significantly lower with semaglutide than placebo (1.29±0.34 mmol/L vs. 1.47±0.36 mmol/L, respectively; placebo-adjusted change: -0.17±0.29; p=0.01), while other blood lipid levels [total cholesterol, triglycerides, LDL cholesterol, non-HDL cholesterol] did not significantly change.<br>• Mean (±SD) body weight and BMI were significantly reduced by 5.3±2.9 kg and 1.9±1.1 kg/m² with semaglutide compared to placebo (p< 0.001).<br>• Mean (±SD) percent body weight reduction was - |

| | | | |
|---|---|---|---|
| | management practices; or any other concern for safety for the participant as per clinical judgment of the investigator.<br><br>• Of the 113 adults with T1D who were initially screened, 28 participants (female participants: n=17; 61%) were recruited and randomized.<br><br>• Baseline characteristics of study participants: mean (±SD) age (n=28), 45±14 years; mean (±SD) diabetes duration (n=28), 28±13 years; mean (±SD) body weight (n=24), 91.3±17.4 kg; mean (±SD) BMI (n=24), 32.3±5.5 kg/m$^2$; mean (±SD) waist circumference (n=24), 101.1±14.3 cm; mean | washout period. After the washout period, the second study drug was started, with identical procedures to the first intervention.<br><br>• The primary endpoint was the percentage of time spent in the target glucose range (3.9-10.0 mmol/L; 70-180 mg/dL) between semaglutide (at maximum tolerated dose) and placebo, during the 4 weeks of AID use.<br><br>• Secondary endpoints included mean glucose level, SD and CV of glucose levels, time spent in hyperglycemia (above 10.0 mmol/L and above 13.9 mmol/L; above 180 mg/dL and above 250 mg/dL) and | 6.7±5.1% with semaglutide and -2.1±3.7% with placebo, constituting a significant -5.1±3.0% relative change from baseline (p< 0.001).<br><br>• Mean (±SD) waist circumference and hip circumference values were both significantly reduced with semaglutide as compared to placebo (placebo-adjusted change: -5.2±4.6 cm and -4.5±3.1 cm, respectively; p<0.001).<br><br>• There were no significant differences in waist-to-hip ratio, heart rate, systolic and diastolic blood pressure values between the interventions.<br><br>• Participants who experienced the greatest weight reduction tended to exhibit the highest glycemic benefits, as supported by the Pearson correlation documenting that the reduction in body weight was significantly correlated with the increase in time in range (Pearson ρ=0.50; p=0.015) and with the reduction in HbA1c (Pearson ρ=0.43; p=0.04). |

| | | | |
|---|---|---|---|
| | (±SD) hip circumference (n=24), 111.8±10.8 cm; mean (±SD) HbA1c (n=28), 7.4±0.8%; median HbA1c (n=24), 7.5% [IQR: 7.1, 7.9]; mean (±SD) daily insulin requirements (n=28), 0.70±0.27 U/kg/day; median total cholesterol (n=24), 4.18 mmol/L [IQR: 3.61, 4.86]; mean (±SD) HDL cholesterol (n=24), 1.46±0.42 mmol/L; mean (±SD) LDL cholesterol (n=24), 2.24±1.03 mmol/L; mean (±SD) non-HDL cholesterol (n=24), 2.90±1.12 mmol/L; median triglycerides (n=24), 1.69 mmol/L [IQR: 0.80, 1.78].<br><br>• At baseline (n=28), 22 participants | time spent in hypoglycemia (below 3.0 mmol/L and below 3.9 mmol/L; below 54 and below 70 mg/dL). | • Median TDD of insulin was significantly reduced by 11.3 units [IQR: -23.6, -4.9] with semaglutide, as compared to placebo (p<0.001); this reduction was mediated by significant reductions in both median basal (-3.5 U [IQR: -9.7, -0.3]; p=0.003) and bolus insulin (-6.2 U [IQR: -14.1, -3.5]; p<0.001).<br><br>• Mean (±SD) weight-based daily insulin requirements (which serve as a marker of insulin sensitivity) were also significantly reduced with semaglutide (0.64±0.28 U/kg/day) as compared to placebo (0.77±0.35 U/kg/day) [paired difference: -0.13±0.16; p=0.002].<br><br>• Median value of total daily carbohydrate intake (as entered into the participants' bolus calculator) was significantly reduced by 36 g [IQR: -55, -15] with semaglutide use as compared to placebo (p<0.001).<br><br>• Median ferritin levels were lower after placebo as |

| | | | |
|---|---|---|---|
| | (79%) were using commercial AID systems, while 7 participants (25%) were affected by overweight (defined by a BMI value ranging between 25.0 and 29.9 kg/m$^2$) and 18 participants (64%) were affected by obesity (defined by a BMI value ≥30.0 kg/m$^2$).<br><br>• Class 1 obesity (BMI: 30.0-34.9 kg/m$^2$): n=11 (39%).<br>• Class 2 obesity (BMI: 35.0-39.9 kg/m$^2$): n=5 (18%).<br>• Class 3 obesity (BMI: ≥40.0 kg/m$^2$): n=2 (7%).<br>• Participants with a BMI lower than 25.0 kg/m$^2$: n=3 (11%).<br><br>• At baseline (n=28), less than one-third of participants (n=8; 29%) | | compared to semaglutide (48 μg/L [IQR: 21, 84] vs. 73 μg/L [IQR: 27, 118], respectively; p=0.007).<br><br>• Three participants had iron deficiency early in the trial: these participants were randomized to placebo first, and then treated with iron supplementation by semaglutide use; when data of these 3 participants were excluded, median HbA1c changes between interventions remained statistically significant (semaglutide, 6.8% [IQR: 6.2, 7.1]; placebo, 7.2% [IQR: 6.5, 7.5]; p<0.001).<br><br>• Median alkaline phosphatase (ALP) values decreased more with semaglutide compared to placebo (58 Units/L [IQR: 51, 66] vs. 63 Units/L [IQR: 53, 77], respectively; p=0.002).<br><br>• Median bilirubin values increased more with semaglutide compared to placebo (10 mmol/L [IQR: 9, 15] vs. 9 mmol/L [IQR: 8, 12], respectively; p=0.02). |

| | | | |
|---|---|---|---|
| | met the HbA1c target (<7%). | | • There were no differences in other laboratory measurements between interventions, including alanine aminotransferase, creatinine, estimated glomerular filtration rate (mL/min/1.73 m$^2$; as per CKD-EPI equation), urine albumin-creatinine ratio, C-reactive protein, Brain natriuretic peptide (NT-proBNP), lipase, and interleukin-6.<br>• 6 out of the 24 participants who completed the trial (25%) had detectable random plasma C-peptide levels at baseline (defined as random plasma C-peptide levels greater than 0.003 nmol/L).<br>• Placebo-adjusted changes in mean (±SD) TIR 3.9-10.0 mmol/L (TIR 70-180 mg/dL), body weight and HbA1c were 6.2±9.7 percentage points, -6.3±5.7 kg and -0.7±0.3%, respectively, in participants with detectable C-peptide levels, as compared to 4.4±7.1 percentage points, -6.4±3.4 kg and -0.4±0.3%, |

| | | | |
|---|---|---|---|
| | | | respectively, in participants with undetectable C-peptide levels.<br><br>• Post-hoc analysis in 12 participants who were on the commercial Control-IQ technology during the last 14 days of the titration period: placebo-adjusted outcomes of insulin use, TIR, TBR and carbohydrate intake were all comparable to the findings observed with the research-based AID system.<br><br>Side effects/adverse reactions:<br><br>• The most common adverse events reported during this trial were gastrointestinal in nature and were mostly mild.<br>• 72 adverse events were reported in 23 (82%) participants with semaglutide, and 14 adverse events were reported in 7 (25%) participants with placebo.<br>• Adverse events occurring in more than 10% of participants included: nausea, loss of appetite, constipation, emesis, diarrhea, gastroesophageal reflux, fatigue, upper |

respiratory tract infection, and lightheadedness.

- Severe hypoglycemic events or DKA were not reported during semaglutide use.
- There was only one episode of severe hypoglycemia with placebo during the titration period, which was related to CGM malfunction.
- Two participants experienced overt episodes of euglycemic ketosis during semaglutide use; these episodes did not lead to acidosis (euglycemic ketosis without acidosis) and were resolved with proper changes in carbohydrate intake and insulin therapy.
- One of the two euglycemic ketosis cases was thought to be related to semaglutide use, while the other case was suspected to be unrelated to semaglutide use since it occurred during a concomitant foodborne illness and insulin pump malfunction.
- In two study participants, progression of clinically stable or regressed retinopathy was incidentally found:

| | | | |
|---|---|---|---|
| | | | one episode of proliferative retinopathy during placebo use, and one non-proliferative sequela during semaglutide therapy; such events were not considered sight-threatening, as per ophthalmology consultation.<br>• There was only one serious adverse event during semaglutide use (a recurrent tibial fracture after a prior surgery), although this was deemed to be unrelated to the study drug. |
| Case report (Da Porto et al. 2024 - Ref. 124) | • 36-year-old woman with a past medical history of autoimmune thyroiditis and gestational diabetes mellitus that required only dietary management.<br>• Clinical presentation: polyuria; recurrent genital infections; unintentional weight loss.<br>• Clinical and laboratory | • 5-year treatment with once-weekly subcutaneous semaglutide, in addition to metformin (at a dose of 1000 mg BID).<br>• The patient was initially treated with metformin (at a dose of 1000 mg twice a day) and basal insulin (insulin glargine U300 at a dose of 10 units/day).<br>• Blood glucose levels | • During the 5-year follow-up period, the patient maintained a good glycemic control until 36 months, when there was a mild increase in Hb1Ac value (7.1%) and semaglutide dose was titrated to 1 mg/week.<br>• The patient exhibited a preserved beta-cell function up to 60 months after the initial diagnosis of LADA.<br>• Anthropometric and laboratory parameters at 60 months: BMI, 21.2 kg/m$^2$; waist circumference, 74 cm; fasting blood glucose, 5.44 mmol/L (98 mg/dL); |

| | | | |
|---|---|---|---|
| | characteristics at the time of presentation: BMI, 20 kg/m$^2$; waist circumference, 76 cm; familiar history of T2D; physically active patient (she played basketball three times a week); random blood glucose value, 315 mg/dL; normal anion gap and bicarbonate values; HbA1c, 12.3% (111 mmol/mol); absence of blood and urinary ketones; positivity of GAD antibodies (162 IU/mL) and antibodies against intracellular epitopes of the tyrosine-phosphatase 2 (43 IU/L); negativity of zinc transporter 8 antibodies (<10 IU/ml).<br>• Genetic testing for MODY (maturity-onset diabetes of | gradually normalized during the first 5 weeks of treatment, although the patient experienced episodes of fasting hypoglycemia.<br>• Basal insulin was therefore interrupted and replaced with once-weekly subcutaneous semaglutide, which was started at an initial weekly dose of 0.25 mg for the first 4 weeks and subsequently titrated to a weekly dose of 0.5 mg.<br>• Semaglutide dose was titrated to 1 mg/week at 36 months. | HbA1c, 5.8%; fasting C-peptide, 1.3 ng/mL; fasting insulinemia, 5.3 µIU/mL; HOMA-IR index, 1.3; peak stimulated C-peptide (during the 180-min MMTT), 4.3 ng/mL. |

| | | | |
|---|---|---|---|
| | the young) was negative.<br><br>• Clinical and laboratory findings led to the diagnosis of LADA, which was associated with a preserved fasting C-peptide (0.65 ng/mL). | | |
| Exploratory single-center, retrospective study (Klein et al. 2024 - Ref. 141) | • 11 adult patients with long-standing T1D and obesity likely related to genetic mutations (“mutation cohort”) vs. 15 adult patients with long-standing T1D and obesity unlikely related to genetic mutations (control group).<br><br>• Adults with a BMI ≥40 kg/m² and a history of early-onset severe obesity were eligible for genetic testing (performed through saliva or blood sample | • Among the 11 patients in the “mutation cohort”, 3 patients (27.3%) used semaglutide and 4 patients (36.3%) used tirzepatide, while the remaining 4 patients used liraglutide (n=1) and dulaglutide (n=3).<br><br>• Among the 15 patients in the control group, 13 patients (86.6%) used tirzepatide, while the remaining 2 patients used liraglutide (n=1) and | • At 6 months from baseline, patients with obesity likely related to genetic mutations, as compared to the control group (patients with obesity unlikely related to genetic mutations), showed a non-significant lower absolute and relative change in body weight (mean±SD: -5.75±9.46 kg vs. -8.65±9.36 kg; p=0.44; -4.78±8.83% vs. -8.57±9.53%; p=0.31), as well as a non-significant lower absolute change in HbA1c (mean±SD: -0.28±0.96% vs. -0.43±0.57%; p=0.64).<br><br>• There were significantly less subjects with obesity likely related to genetic mutations, as |

| | | | |
|---|---|---|---|
| | collection) aimed at screening 79 genes mainly associated with the leptin-melanocortin pathway, Bardet-Biedl syndrome (and related ciliopathies) and genes affecting energy balance.<br><br>• The genetic mutations screened were mainly associated with the functionality of hypothalamic regulatory centers, which represent sites of endogenous GLP-1 action and play a critical role in the regulation of energy intake and expenditure.<br><br>• The majority of patients in the "mutation cohort" were heterozygous for the mutations screened. | exenatide (n=1) [no one used semaglutide].<br><br>• Duration of the observation period: 6 months.<br><br>• Data on dosages of GLP-1 RAs and tirzepatide over time in the two groups were not available.<br><br>• The primary outcome of this study was percentage change in body weight and absolute change in HbA1c at 6 months from baseline across the two groups.<br><br>• Response to incretin therapy was defined based on the achievement of a body weight reduction equal to or greater than 5% at 6 months from | compared to subjects with obesity unlikely related to genetic mutations, who met either HbA1c reduction of ≥0.4% or weight loss of ≥5% at 6 months from baseline (36.36% vs. 80.0%; p=0.04). |

| | | | |
|---|---|---|---|
| | • Adults with T1D who had participated in the "Uncovering Rare Obesity" program (through which eligible subjects were tested for genetic forms of obesity) and used incretin analogs (including GLP-1 RAs and the dual GIP/GLP-1 RA tirzepatide) for the management of obesity were enrolled in this study.<br><br>• Baseline characteristics of the study participants (baseline was defined as the date of initiation of incretin therapies): median age, 39.5 years (IQR: 35.3-44.1) [mutation cohort] vs. 45.8 years (IQR: 34.9-48.0) [control group]; | baseline and/or on the achievement of an HbA1c reduction equal to or greater than 0.4% at 6 months from baseline. | |

| |
|---|
| female sex [n (%)], 5 (45.5%) [mutation cohort] vs. 11 (73.3%) [control group]; non-Hispanic White [n (%)], 9 (81.8%) [mutation cohort] vs. 14 (93.3%) [control group]; median diabetes duration, 12.8 years (IQR: 7.5-26.1) [mutation cohort] vs. 24.0 years (IQR: 15.3-42.7) [control group]; median body weight, 128.2 kg (IQR: 113.0-139.2) [mutation cohort] vs. 109.7 kg (IQR: 92.1-117.3) [control group]; mean (±SD) body weight, 125.92±22.52 kg [mutation cohort] vs. 105.90±21.97 kg [control group] [p=0.01]; median BMI, 43.0 kg/m$^2$ (IQR: |

| |
|---|
| 38.7-43.9) [mutation cohort] vs. 38.7 kg/m$^2$ (IQR: 33.7-41.5); median HbA1c, 7.3% (IQR: 6.9-8.2) [mutation cohort] vs. 7.2% (IQR: 6.9-7.6) [control group]; mean (±SD) HbA1c, 7.58±1.26% [mutation cohort] vs. 7.27±0.65% [control group]; MDI insulin therapy [n (%)], 2 (18.2%) [mutation cohort] vs. 8 (53.3%) [control group]; insulin pump w/o CGM [n (%)], 2 (18.2%) [mutation cohort] vs. 0 (0.0%) [control group]; AID [n (%)], 7 (63.6%) [mutation cohort] vs. 7 (46.7%) [control group]; use of CGM [n (%)], 8 (72.7%) [mutation cohort] vs. 12 (80.0%) [control group]; |

| | | | |
|---|---|---|---|
| | median TDD of insulin, 0.68 units/kg/day (IQR: 0.56-0.89) [mutation cohort] vs. 0.46 units/kg/day (IQR: 0.42-0.64) [control group]; presence of dyslipidemia [n (%)], 5 (45.5%) [mutation cohort] vs. 8 (53.3%) [control group]; presence of retinopathy [n (%)], 4 (36.4%) [mutation cohort] vs. 9 (60.0%) [control group].<br>• There was no patient with cardiovascular disease, neuropathy or nephropathy in both groups. | | |

Abbreviations: AID, Automated insulin delivery; ALP, Alkaline phosphatase; AYA, Adolescents and young adults; BES, Binge Eating Scale; BID, Twice a day; BMI, Body mass index; CGM, Continuous glucose monitoring; CKD-EPI, Chronic Kidney Disease Epidemiology Collaboration; COVID-19, Coronavirus disease 2019; CSII, Continuous subcutaneous insulin infusion; CV, Coefficient of variation; DASS-21, 21-item Depression Anxiety and Stress Scale; DKA, Diabetic ketoacidosis; eGDR, estimated glucose disposal rate; eGFR, estimated glomerular filtration rate; eGFRcys, eGFR based on cystatin C; FGM, Flash glucose monitoring; FMD, Brachial artery flow-mediated dilation; GAD, Glutamic acid decarboxylase; GIP, Glucose-dependent insulinotropic polypeptide; GLP-1, Glucagon-like peptide-1; GMI, Glucose Management Indicator; HbA1c, Glycated hemoglobin; HDL, High-density lipoprotein; HOMA-IR, Homeostasis model assessment of insulin resistance; IQR, Interquartile range; LDL, Low-density lipoprotein; MDI, Multiple daily injection; MMTT, Mixed meal tolerance test; MODY, Maturity-onset diabetes of the young; ns, non-significant; NT-proBNP, Brain natriuretic peptide; OSA, Obstructive sleep apnea; p, p-value; PCOS, Polycystic ovary syndrome; PLGM, Predictive low-glucose management; PR, Peripheral resistance; RA, Receptor agonist; RAs, Receptor agonists; sc, subcutaneous; SD, Standard deviation; SE, Standard

error; SmPC, Summary of Product Characteristics; T1D, Type 1 diabetes; T2D, Type 2 diabetes; TAR, Time above range; TBR, Time below range; TDD, Total daily dose; TIR, Time in range.
