# Supplementary material for: Unveiling the Therapeutic Potential of the Second-Generation Incretin Analogs Semaglutide and Tirzepatide in Type 1 Diabetes and Latent Autoimmune Diabetes in Adults
Source: J Clin Med. 2025 Feb 15;14(4):1303. doi: 10.3390/jcm14041303 (PMC11856673; doi:10.3390/jcm14041303)
Supplement: Supplementary file 1 [file jcm-14-01303-s001.zip › jcm-3374466 - Supplementary Table S2.pdf]

Table S2. Clinical studies investigating the use of tirzepatide in patients with autoimmune diabetes.

Study Design	Study Population	Study Treatment and Duration	Main Findings
Case report (Sharma et al. 2023 - Ref. 129)	• 36-year-old woman with T1D and class 1 obesity (BMI: 32.2 kg/m^2; body weight: 192 lbs) on a closed-loop insulin infusion system (Tandem t:slim X2 insulin pump with Control-IQ technology; Tandem Diabetes Care, Inc., San Diego, CA, USA) and tirzepatide therapy.<br>• The patient experienced DKA after insulin pump infusion set failure as a likely consequence of volume depletion precipitated by side effects of tirzepatide (severe nausea, vomiting and minimal oral intake).	• Tirzepatide was prescribed (off-label) due to the presence of class 1 obesity (BMI: 32.2 kg/m^2; body weight: 192 lbs) associated with poorly controlled diabetes (HbA1c: 9.4%; 79 mmol/mol).<br>• The exact tirzepatide dose and titration scheme were not specified in the manuscript.<br>• Duration of tirzepatide therapy: 3 months.	• At 3 months from tirzepatide initiation, the patient had lost 50 lbs (body weight: 142 lbs; BMI: 25.3 kg/m^2), but reported severe nausea and heartburn related to tirzepatide therapy.<br>• Given the ongoing weight loss, the patient decided to continue using tirzepatide despite the medical advice to stop or decrease the drug dose.<br>• The patient presented to the emergency room 4 days after the last tirzepatide injection, reporting acute worsening of heartburn, nausea, vomiting and minimal oral intake for 3 days; home urine ketone testing was positive.<br>• The patient was alert and afebrile; she had tachycardia, tachypnea, Kussmaul breathing and dry oral mucosa, suggesting volume depletion.<br>• At that time, the patient's TDD of insulin was 30 units, while the patient's TDD of insulin was closer to

70 units before tirzepatide initiation.

- The closed-loop insulin infusion system showed intermittent insulin delivery suspensions due to intermittent hypoglycemic episodes reported by the CGM sensor (Dexcom G6).

- A point-of-care blood glucose testing showed a glucose level of 289 mg/dL, which was comparable to the serum glucose level detected with laboratory tests (322 mg/dL), whereas concurrent CGM reading showed an interstitial glucose level of 40 mg/dL.

- Further laboratory tests showed the following results: hemoglobin, 16.2 g/dL (reference range: 11.2-15.7 g/dL); hematocrit, 45.3% (reference range: 34.1%-44.9%); HbA1c, 7.4% (57 mmol/mol); bicarbonate, 12 mmol/L (reference range: 20-31 mmol/L); sodium, 131 mmol/L (reference range: 136-145 mmol/L); potassium, 5.0 mmol/L (reference range: 3.5-5.1 mmol/L); serum creatinine, 1.00 mg/dL (reference range: 0.55-1.02 mg/dL); glomerular filtration rate, 75 mL/min/1.73 m^2; anion gap, 21 mmol/L (reference range: 6-14

mmol/L); negative urine human chorionic gonadotropin (hCG).

- These results confirmed the presence of high-anion-gap metabolic acidosis: the patient was therefore diagnosed with DKA (despite beta-hydroxybutyrate test was not performed) based on elevated serum glucose values associated with high-anion-gap metabolic acidosis and with a history of positive urine ketones on home urine testing.

- Physical examination and laboratory tests did not show findings suggestive of infection, while volume depletion was attributed to the gastrointestinal side effects of tirzepatide.

- The insulin pump and CGM sensor were removed, and the patient was treated with intravenous insulin and fluid therapy [continuous regular insulin (TDD of insulin: 33 units) plus normal saline].

- Serum glucose values improved (143 mg/dL) and anion gap decreased (8 mmol/L) within 8 hours, while normalization of serum bicarbonate values (24

			mmol/L) occurred within 72 hours.<br>• Afterwards, the patient was first transitioned to MDI insulin therapy with insulin pens (basal insulin glargine plus prandial insulin lispro; TDD of insulin: 24 units) and then to the closed-loop insulin infusion systems (Tandem t:slim X2 insulin pump with Control-IQ technology) with Dexcom G6 CGM at pre-admission settings.<br>• Glucose values remained within the optimal range (TDD of insulin: 37 units) for the subsequent 24 hours and until the outpatient follow-up visit 10 days later, while tirzepatide was definitively discontinued.
Case report (Mendoza and Parsiani; 2023 - Ref. 131)	• 23-year-old woman with long-standing T1D (diagnosed at the age of 10 years) and class 2 obesity (BMI: 38 kg/m^2) who referred to the outpatient clinic due to increasing insulin requirements and insulin resistance.	• 12-week once-weekly subcutaneous tirzepatide therapy.<br>• The patient was prescribed once-weekly subcutaneous tirzepatide	• Within the first month of tirzepatide therapy, the patient experienced a weight loss of 5 pounds.<br>• By week 9, the patient's weight decreased further to 188 pounds (- 7 lbs; BMI: 36 kg/m^2) and her HbA1c decreased to 6.9%.<br>• By week 12, the patient reported a 24% decrease in daily carbohydrate consumption (from an average

	• The patient was using CGM and insulin pump therapy (Omnipod 5) with hybrid closed-loop technology in automated mode (100% of the time), which remained consistent during the subsequent weeks.<br><br>• The patient's last HbA1c value (measured one month before starting tirzepatide therapy) was 7.4%, and her body weight was 195 pounds [BMI: 38 kg/m^2].<br><br>• The patient's body weight had increased by about 40 pounds in the last year.<br><br>• Exercise was limited due to a busy schedule, amounting to less than 10 thousand steps per day.<br><br>• The patient was using an average daily dose of basal and prandial	therapy at an initial weekly dose of 2.5 mg, which was initially titrated to 5 mg after 4 weeks, and to 7.5 mg after the subsequent 4 weeks.<br><br>• Over the course of the 12-week tirzepatide therapy, the patient continued to use metformin at a daily dose ranging from 1000 mg to 2000 mg.	of 119.5 g to 90.9 g), although weight data were not available at this time.<br><br>• At 12 weeks, the TDD of insulin decreased to 57.6 units (daily doses of basal and prandial insulin: 39.8 and 17.8 units, respectively).<br><br>• CGM metrics after 12 weeks of tirzepatide therapy: average glucose, 176 mg/dL (-51 mg/dL); GMI, 7.5% (-1.2%); SD, 46 mg/dL (-26 m/dL); CV, 26.1% (-5.7%); TIR 70-180 mg/dL, 61% (+30%); TAR 181-250 mg/dL, 32% (unchanged as compared to baseline); TAR >250 mg/dL, 7% (-30%); TBR 54-69 mg/dL, 0% (unchanged as compared to baseline); TBR <54 mg/dL, 0% (unchanged as compared to baseline).<br><br>Side effects/adverse reactions:<br><br>• The main side effects of tirzepatide therapy were mild transient nausea (experienced with the 2.5 mg and 7.5 mg weekly doses), transient vomiting (experienced with the 7.5 mg weekly dose, for about 2 days after the drug injection) and transient hypoglycemic episodes (around the third

	insulin of 55.4 units and 26.5 units, respectively (TDD of insulin: 81.9 units).<br>• The month earlier, the patient had started extended-release metformin (titrated to 1000 mg/day) to reduce insulin resistance and insulin requirements and improve glucose control.<br>• CGM metrics at baseline: average glucose, 227 mg/dL; GMI, 8.7%; SD, 72 mg/dL; CV, 31.8%; TIR 70-180 mg/dL, 31%; TAR 181-250 mg/dL, 32%; TAR >250 mg/dL, 37%; TBR 54-69 mg/dL, 0%; TBR <54 mg/dL, 0%.		week on the 2.5 mg weekly dose).<br>• Hypoglycemic episodes were not long enough to affect the patient's TBR and disappeared after the adjustment of the insulin-to-carbohydrate ratios (from 1:4 to 1:6).
Single-center retrospective observational study (Akturk et al. 2024 - Ref. 125)	• 26 adult patients with T1D.<br>• Baseline characteristics of the study participants: 14 participants	• The starting weekly dose of tirzepatide for all patients was 2.5	• Changes from baseline in HbA1c, body weight, TDD of insulin and CGM metrics were expressed as least squares means. There was a significant reduction in HbA1c value by

	(54%) were female; race/ethnicity: non-Hispanic White, 20 (77%); other, 6 (33%); mean (±SD) age, 42±8 years (age range: 28-56 years); mean (±SD) body weight, 108.1±21.2 kg (body weight range: 74.4-158.8 kg); mean (±SD) BMI, 36.7±5.3 kg/m^2 (BMI range: 24.9-44.7 kg/m^2); mean (±SD) HbA1c, 7.3±0.7% (HbA1c range: 6.1-8.6%); mean (±SD) TDD of insulin, 83.9±44.7 IU/day (range: 36-174 IU/day).<br>• Diabetes duration was not specified in the manuscript.<br>• 25 patients (96%) used a CGM sensor, 7 patients (27%) were on MDI insulin therapy, and 19 patients (73%) were on insulin pump therapy.	mg, even though dose adjustments varied among patients and the tirzepatide doses were up-titrated based on the individual glycemic and/or weight loss goals.<br>• Out of 26 patients, 25, 25 and 24 patients were still using tirzepatide at 3, 6, and 8 months, respectively.<br>• At 8 months, 7 patients were on 7.5 mg and 10 mg weekly tirzepatide doses, while the remaining	0.45% at 3 months (p=0.027) and sustained over the 8-month period (-0.59% at 8 months).<br>• There was a significant body weight loss over the 8-month period. The percent change in body weight from baseline was -3.4% at 3 months and -10.5% at 6 months, although no further body weight loss was observed after 6 months.<br>• There was a significant percent change in BMI from baseline at 3 months (-3.35%) and at 6 months (-10.5%).<br>• The TDD of insulin decreased by 21.6 IU/day at 3 months, by 22.9 IU/day at 6 months, and by 24.3 IU/day at 8 months.<br>• There was a significant increase in TIR 70-180 mg/dL by 12.6% at 3 months (p=0.0002), which was sustained over the 8-month period and accompanied by a significant increase in time in tight target range (TITR) 70-140 mg/dL by 10.7% at 3 months (p=0.0016) and 11.5% at 6 months (p=0.0008).<br>• There was a significant reduction in TAR >180 mg/dL by 12.6%

	• Three patients were using MDI insulin therapy at baseline but transitioned to insulin pump therapy during the follow-up.	patients were on weekly tirzepatide doses varying from 5 to 15 mg.<br>• None of the patients were using other oral glucose-lowering medications such as metformin.<br>• Duration of the observation period: 8 months.	at 3 months (p=0.0002).<br>Side effects/adverse reactions:<br>• There was no change in TBR (reported as the percentage of time spent with interstitial glucose values below 70 mg/dL), whereas a severe hypoglycemic episode (n=1) and severe constipation (n=1) led to tirzepatide discontinuation in two patients.<br>• One patient experienced peroneal nerve palsy (foot drop) as a possible consequence of rapid weight loss.<br>• No DKA event was reported during the 8-month observation period.
Retrospective single-center real-world study (Garg et al. 2024 - Ref. 126)	• 62 adult patients with long-standing T1D and overweight/obesity who were prescribed tirzepatide (tirzepatide group).<br>• For the data analysis, Authors used a control group including 37 patients with overweight or obesity who were not using any other	• The starting weekly tirzepatide dose was 2.5 mg, with mean (±SD) weekly tirzepatide dose being 5.6±1.9 mg at 3 months, 8.6±3.0 mg at 6 months, 8.8±2.9	• Body weight and BMI decreased significantly at each time point (3, 6, 9 and 12 months) in the tirzepatide group as compared to the control group.<br>• Mean (±SD) body weight decreased significantly by 21.4±13.0 lbs (percentage of weight loss: 9.6±5.3%) at 3 months (p<0.0001) and by 46.5±28.1 lbs (percentage of weight loss: 18.5±10.9%) at 12 months (p<0.0001) in the tirzepatide group.

	weight-loss medication during the same period and were computer-frequency matched by 5-year age groups, 10-year duration of diabetes groups, gender, BMI groups (BMI 27 to <30 kg/m^2, 30 to <35 kg/m^2, 35 to <40 kg/m^2, and ≥40 kg/m^2) and glucose control groups (HbA1c <7%, 7-8%, and >8%).<br>• Inclusion criteria for this analysis were the following: patients age between 18 and 80 years; use of tirzepatide for at least 3 months; BMI ≥27 kg/m^2; intensive insulin therapy using either MDI insulin therapy or an insulin pump or a hybrid closed-loop system; use of a CGM sensor.	mg at 9 months, and 9.7±3.3 mg at 12 months<br>• The number of patients reaching the weekly tirzepatide doses of 10 mg, 12.5 mg and 15 mg was 16, 6 and 6, respectively.<br>• Duration of the follow-up period: 12 months.	• Mean (±SD) BMI decreased significantly by 3.4±2.0 kg/m^2 at 3 months (p <0.0001) and by 6.5±4.3 kg/m^2 at 12 months (p <0.0001).<br>• In univariate comparisons, mean (±SD) HbA1c decreased significantly at all time points in the tirzepatide group as compared to the control group, particularly by 0.50±0.52% at 3 months (p<0.0001) and by 0.67±0.56% at 12 months (p=0.0041).<br>• Changes in HbA1c remained significantly greater in the tirzepatide as compared to the control group even when adjusted for baseline HbA1c in linear mixed models (p<0.0001).<br>• Mean (±SD) TDD of insulin decreased significantly in the tirzepatide group, as compared to the control group, at all time points, particularly by 26.1±23.5 U/day at 3 months (p=0.0088) and by 22.8±19.7 U/day at 12 months (p=0.0006).<br>• There were significant decreases in mean (±SD) CGM glucose in the tirzepatide group, as compared to the control group, at 3 months (-16.8±17.9 mg/dL vs. -1.6±13.2

	• Exclusion criteria were the following: pregnancy; T2D; missing data or data not available for more than 3 months.<br><br>• Baseline characteristics of the study participants: mean (±SD) age, 40±10 years (tirzepatide group) vs. 41±10 years (control group); mean (±SD) diabetes duration, 24±13 years (tirzepatide group) vs. 27±13 years (control group); gender (% [n] male), 27 (17) [tirzepatide group] vs. 27 (10) [control group]; non-Hispanic White (% [n]), 89 (55) [tirzepatide group] vs. 97 (36) [control group]; mean (±SD) body weight, 228.9±42.9 lbs (tirzepatide group) vs. 208.7±31.9 lbs (control group); mean (±SD) BMI,	mg/dL; p=0.0038), 6 months (-19.1±24.4 vs. -3.3±16.2 mg/dL; p=0.0038) and 12 months (-23.5±29.2 mg/dL vs. -5.6±10.3 mg/dL; p=0.0297).<br><br>• There were significantly greater increases in mean (±SD) TIR 70-180 mg/dL in the tirzepatide group, as compared to the control group, at 3 months (+10.5±10.2% vs. +0.2±6.8%; p=0.0006) and at 6 months (+10.8±14.6% vs. +1.7±7.1%; p=0.0022).<br><br>• There were greater increases in mean (±SD) TIR 70-180 mg/dL in the tirzepatide group than in the control group at 9 months (+9.3±10.5% vs. +1.0±8.9%; p=0.0538) and at 12 months (+12.5±15.0% vs. 4.8±12.8%; p=0.1725), although these changes did not reach statistical significance.<br><br>• There were significantly greater decreases in TAR >180 mg/dL in the tirzepatide group, as compared to the control group, at 3 months (-11.0±10.7% vs. -0.5±7.4%; p=0.0008), at 6 months (-11.8±14.7% vs. -2.3±7.8%; p=0.0022) and at 9 months (-9.3±10.6% vs. +0.7±9.3%; p=0.0245).

	35.6±5.5 kg/m^2 (tirzepatide group) vs. 32.8±3.7 kg/m^2 (control group); mean (±SD) HbA1c, 7.0±0.9% (tirzepatide group) vs. 6.7±0.7% (control group); mean (±SD) TDD of insulin, 76±40 U/day (tirzepatide group) vs. 61±25 U/day (control group).<br><br>• At baseline, gender distribution, mean age, ethnicity, duration of diabetes, and HbA1c were similar in the two groups.<br><br>• Mean body weight, BMI, and TDD of insulin were higher in the tirzepatide group than in the control group.<br><br>• Of the 62 patients in the treated group, 52 patients were using insulin pump/ hybrid closed-loop system, and 10 patients were on	• There was also a greater decrease in mean (±SD) TAR >180 mg/dL in the tirzepatide group, as compared to the control group, at 12 months (-13.5±15.2% vs. -5.1±11.2%; p=0.1268), although this decrease was not statistically significant.<br><br>• There were significantly greater decreases in mean (±SD) CGM SD of glucose in the tirzepatide group, as compared to the control group, at 6 months (-9.5±10.1 mg/dL vs. -2.1±9.9 mg/dL; p=0.0067), at 9 months (-9.9±9.6 mg/dL vs. -1.4±6.4 mg/dL; p=0.0247), and at 12 months (-11.0±8.5 mg/dL vs. -0.8±8.7 mg/dL; p=0.0051), even though no significant difference in CV was observed between the tirzepatide group and the control group at any time point.<br><br><u>Side effects/adverse reactions</u>:<br><br>• TBR <70 mg/dL did not change significantly in either group at any time point, and there was no significant difference in the change in TBR <70 mg/dL between the two groups.

	MDI insulin therapy; similarly, 32 patients and 5 patients were using insulin pump/hybrid closed-loop system in the control group, respectively.		• There were no reported hospitalizations due to severe hypoglycemia or DKA.
Exploratory single-center, retrospective study (Klein et al. 2024 - Ref. 141)	• 11 adult patients with long-standing T1D and obesity likely related to genetic mutations ("mutation cohort") vs. 15 adult patients with long-standing T1D and obesity unlikely related to genetic mutations (control group).<br>• Adults with a BMI ≥40 kg/m² and a history of early-onset severe obesity were eligible for genetic testing (performed through saliva or blood sample collection) aimed at screening 79 genes mainly associated with the leptin-melanocortin pathway, Bardet-Biedl	• Among the 11 patients in the "mutation cohort", 3 patients (27.3%) used semaglutide and 4 patients (36.3%) used tirzepatide, while the remaining 4 patients used liraglutide (n=1) and dulaglutide (n=3).<br>• Among the 15 patients in the control group, 13 patients (86.6%) used tirzepatide, while the	• At 6 months from baseline, patients with obesity likely related to genetic mutations, as compared to the control group (patients with obesity unlikely related to genetic mutations), showed a non-significant lower absolute and relative change in body weight (mean±SD: -5.75±9.46 kg vs. -8.65±9.36 kg; p=0.44; -4.78±8.83% vs. -8.57±9.53%; p=0.31), as well as a non-significant lower absolute change in HbA1c (mean±SD: -0.28±0.96% vs. -0.43±0.57%; p=0.64).<br>• There were significantly less subjects with obesity likely related to genetic mutations, as compared to subjects with obesity unlikely related to genetic mutations, who met either HbA1c reduction of ≥0.4% or weight loss of ≥5% at 6 months from baseline (36.36% vs. 80.0%; p=0.04).

	syndrome (and related ciliopathies) and genes affecting energy balance.<br><br>• The genetic mutations screened were mainly associated with the functionality of hypothalamic regulatory centers, which represent sites of endogenous GLP-1 action and play a critical role in the regulation of energy intake and expenditure.<br><br>• The majority of patients in the "mutation cohort" were heterozygous for the mutations screened.<br><br>• Adults with T1D who had participated in the "Uncovering Rare Obesity" program (through which eligible subjects were tested for genetic forms of obesity) and used incretin analogs (including GLP-	remaining 2 patients used liraglutide (n=1) and exenatide (n=1) [no one used semaglutide].<br><br>• Duration of the observation period: 6 months.<br><br>• Data on dosages of GLP-1 RAs and tirzepatide over time in the two groups were not available.<br><br>• The primary outcome of this study was percentage change in body weight and absolute change in HbA1c at 6 months from baseline across	

	1 RAs and the dual GIP/GLP-1 RA tirzepatide) for the management of obesity were enrolled in this study.<br><br>• Baseline characteristics of the study participants (baseline was defined as the date of initiation of incretin therapies): median age, 39.5 years (IQR: 35.3-44.1) [mutation cohort] vs. 45.8 years (IQR: 34.9-48.0) [control group]; female sex [n (%)], 5 (45.5%) [mutation cohort] vs. 11 (73.3%) [control group]; non-Hispanic White [n (%)], 9 (81.8%) [mutation cohort] vs. 14 (93.3%) [control group]; median diabetes duration, 12.8 years (IQR: 7.5-26.1) [mutation cohort] vs. 24.0 years (IQR: 15.3-42.7) [control	the two groups.<br><br>• Response to incretin therapy was defined based on the achievement of a body weight reduction equal to or greater than 5% at 6 months from baseline and/or on the achievement of a HbA1c reduction equal to or greater than 0.4% at 6 months from baseline.	

group]; median body weight, 128.2 kg (IQR: 113.0-139.2) [mutation cohort] vs. 109.7 kg (IQR: 92.1-117.3) [control group]; mean (±SD) body weight, 125.92±22.52 kg [mutation cohort] vs. 105.90±21.97 kg [control group] [p=0.01]; median BMI, 43.0 kg/m^2 (IQR: 38.7-43.9) [mutation cohort] vs. 38.7 kg/m^2 (IQR: 33.7-41.5); median HbA1c, 7.3% (IQR: 6.9-8.2) [mutation cohort] vs. 7.2% (IQR: 6.9-7.6) [control group]; mean (±SD) HbA1c, 7.58±1.26% [mutation cohort] vs. 7.27±0.65% [control group]; MDI insulin therapy [n (%)], 2 (18.2%) [mutation cohort] vs. 8 (53.3%) [control group]; insulin pump w/o CGM [n (%)], 2 (18.2%) [mutation cohort] vs. 0

	(0.0%) [control group]; AID [n (%)], 7 (63.6%) [mutation cohort] vs. 7 (46.7%) [control group]; use of CGM [n (%)], 8 (72.7%) [mutation cohort] vs. 12 (80.0%) [control group]; median TDD of insulin, 0.68 units/kg/day (IQR: 0.56-0.89) [mutation cohort] vs. 0.46 units/kg/day (IQR: 0.42-0.64) [control group]; presence of dyslipidemia [n (%)], 5 (45.5%) [mutation cohort] vs. 8 (53.3%) [control group]; presence of retinopathy [n (%)], 4 (36.4%) [mutation cohort] vs. 9 (60.0%) [control group].<br><br>• There was no patient with cardiovascular disease, neuropathy or nephropathy in both groups.	

Pilot, single-center, retrospective observational study (Karakus et al. 2024 - Ref. 127)	• 11 adult patients with long-standing T1D who were using an AID system (Tandem t:slim X2 with Control-IQ technology) and who received tirzepatide as a non-insulin adjunct therapy.<br>• Authors analyzed changes in body weight, insulin requirements and markers of glucose control over an 8-month period after the initiation of tirzepatide therapy.<br>• Data were collected at different time intervals (0-2 months, 2-3 months, 3-6 months, and 6-8 months intervals) and compared to baseline.<br>• Three months of CGM data before the first tirzepatide dose were collected as the	• The starting weekly dose of tirzepatide for all patients was 2.5 mg and the doses were up-titrated and adjusted by physicians based on the individual patients' weight loss and/or glycemic goal and adverse drug events.<br>• Duration of the observation period: 8 months.<br>• Of 11 participants, 2 were on 2.5 mg weekly tirzepatide dose, 4 were on 5.0 mg weekly tirzepatide dose, and 5	• There was a significant reduction in TDD of insulin from a median of 73.9 units/day (IQR: 47.6-95.8) at the baseline to 51.7 units/day (IQR: 46.7-66.8) at 0-2 months interval (corresponding to a 30% reduction in TDD of insulin within 2 months after the initiation of tirzepatide therapy), with less pronounced subsequent reductions: median of 46.2 units/day (IQR: 40.9-74.2) at 2-3 months interval; median of 45.3 units/day (IQR: 34.2-73.3) at 3-6 months interval; median of 41.8 units/day (IQR: 32.0-66.4) at 6-8 months interval ($p<0.01$ for all changes in TDD of insulin as compared to baseline).<br>• There was a significant reduction in both median basal insulin and bolus insulin doses within the first 2 months from baseline (31% reduction in basal insulin dose; 43% reduction in bolus insulin dose), and this reduction remained significantly sustained over 8 months: median basal insulin, from 47 units/day (IQR: 28.2-51.8) at baseline to 25.6 units/day (IQR: 20.8-39.0) at 6-8 months interval ($p=0.005$); median bolus insulin, from 31.4

	baseline CGM data.<br>• Baseline characteristics of the study participants: median age, 37 years (min-max: 34-49); sex (female) [n (%)], 7 (63.6%); race/ethnicity (non-Hispanic White) [n (%)], 10 (90.9%); median diabetes duration, 24 years (min-max: 15-35); median body weight, 114.3 kg (IQR: 94.8-129.3); median BMI, 39.6 kg/m² (IQR: 35.6-40.7); median TDD of insulin, 73.9 units/day (IQR: 47.6-95.8); median HbA1c, 7.0% (IQR: 6.7-7.4).	were on 7.5 mg weekly tirzepatide dose at 3 months.<br>• At 6 months, 2 participants were on 5.0 mg weekly tirzepatide dose, 3 were on 7.5 mg weekly tirzepatide dose, 3 were on 10.0 mg weekly tirzepatide dose, one was on 12.5 mg weekly tirzepatide dose, and 2 were on 15.0 mg weekly tirzepatide dose.<br>• At 8 months, 2, 1, 3, 3, and 2 patients were on 5.0, 7.5, 10.0,	units/day (IQR: 19.9-38.3) to 13.0 units/day (IQR: 10.4-24.1) at 6-8 months interval (p=0.002).<br>• The largest percent reduction in median bolus insulin dose was seen within the first 2 months after the initiation of tirzepatide therapy (43%).<br>• TDD of insulin expressed as units/kg/day (n=8) also decreased significantly from a median of 0.53 units/kg/day (IQR: 0.44-0.83) at baseline to a median of 0.40 units/kg/day (IQR: 0.32-0.58) at 6 months from baseline (p=0.017) (corresponding approximately to a 25% reduction as compared to baseline).<br>• There was a significant reduction in the doses of both user-initiated insulin boluses and automated correction insulin boluses during the first 2 months, and this reduction remained significantly sustained over 8 months.<br>• There was a significant reduction in the number of automated correction insulin bolus counts during the 8-month period.

12.5, and 15.0 mg weekly tirzepatide doses, respectively.	• There was no significant change in the number of user-initiated insulin bolus counts during the 8-month period (p=ns for all time points), even though the amount of insulin in such boluses decreased significantly.<br><br>• Carbohydrate entry into the insulin pump decreased during the 8-month period after the initiation of tirzepatide therapy, with mean (±SD) daily carbohydrate consumption declining from 120±67 g to 70±37 g at 0-2 months interval, 81±64 g at 2-3 months interval, 66±89 g at 3-6 months interval, and 45±103 g at 6-8 months interval (p-values not shown).<br><br>• Body weight and BMI (n=9) decreased significantly from a median of 114.3 kg (IQR: 94.8-129.3) at baseline to a median of 105.7 kg (IQR: 95.2-110.2) at 6 months (p=0.015) and from a median of 39.6 kg/m^2 (IQR: 37.1-40.6) at baseline to a median of 36.3 kg/m^2 (IQR: 33.4-38.5) at 6 months (p=0.015), respectively.<br><br>• Median HA1c values decreased from 7.0% (IQR: 6.7-7.4) to 6.3% (IQR: 5.8-7.2) after tirzepatide therapy, although this change

	was not statistically significant (p=0.063).<br><br>• There was a significant increase in TIR 70-180 mg/dL within the first 2 months after the initiation of tirzepatide therapy, and this increase remained significantly sustained throughout the subsequent time intervals: median TIR 70-180 mg/dL, from 69.8% (IQR: 56.9-72.2) at baseline to 75.6% (IQR: 69.9-80.8) at 6-8 months interval (p=0.015).<br><br>• There was no significant change in TBR <70 mg/dL between baseline and all the subsequent analyzed time intervals (median TBR values at baseline, 0-2 months interval, 2-3 months interval, 3-6 months interval, and 6-8 months interval: 0.8%, 0.6%, 0.6%, 0.6%, and 0.7%, respectively; p=ns for all time intervals).<br><br>• Ambulatory glucose profile (AGP) showed improvement in interstitial glucose values throughout the day after tirzepatide therapy, which was more pronounced during daytime and was also accompanied by lower postprandial glucose values.

Retrospective study (Rivera Gutierrez et al. 2024 - Ref. 128)	• Retrospective review of electronic medical records of adults with T1D (≥18 years of age) who were prescribed tirzepatide for the treatment of overweight and obesity at Mayo Clinic (between June 1, 2022, and October 31, 2023).<br>• Inclusion criteria: age ≥18 years; established diagnosis of T1D; use of once-weekly subcutaneous tirzepatide for the treatment of obesity (BMI ≥30.0 kg/m^2) or overweight (BMI ≥27.0 kg/m^2) associated with at least one adiposity-related disease.<br>• Exclusion criteria: use of tirzepatide for less than 3 months; use of other U.S. Food and Drug Administration (FDA)-approved anti-obesity drugs	• All the study participants had used tirzepatide for at least 3 months.<br>• Authors categorized weekly subcutaneous tirzepatide dosages as "low dose" (2.5-5.0 mg), "medium dose" (7.5.10.0 mg) and "high dose" (12.5-15.0 mg).<br>• Maximum tirzepatide dose achieved by study participants: high dose, n=18 (35.3%); medium dose, n=21 (41.2%); low dose,	• At the last follow-up, there was a significant reduction in median TBWL%, which amounted to -8.5% (Q1-Q3: -5.3% to -13.8%) [p<0.0001].<br>• By the last follow-up, 76.5% of patients (n=39) achieved at least 5% of TBWL, while all patients with available data at 12 months of follow-up achieved at least 5% of TBWL. The reduction in TBWL% was significant (p<0.0001) at all time points: 3 months, -6%; 6 months, -9%; 9 months, -10%; 12 months, -12.2%.<br>• At the last follow-up, there was a significant reduction in median HbA1c by 0.9% (Q1-Q3: 0.3-1.1%) [p<0.0001].<br>• At the last follow-up, there was a significant reduction in median HbA1c by 0.9% (p<0.0001).<br>• The improvement in HbA1c values was mirrored by significant changes in CGM metrics among CGM users, namely: a significant increase in median TIR 70-180 mg/dL (from 51.0% at baseline to 69.0% at the last follow-up; p<0.0001); and a significant decrease in median TAR >180

	within 3 months of tirzepatide therapy initiation (semaglutide, liraglutide, phentermine/topiramate extended-release, phentermine, and/or bupropion/naltrexone sustained-release); history of bariatric surgery and/or active malignancy.<br>• Overall, 143 adults with T1D who were prescribed tirzepatide were identified and 51 patients were subsequently included in the study.<br>• 92 patients who did not fulfill inclusion and/or exclusion criteria were excluded from the study (mainly due to tirzepatide insurance denial).<br>• Baseline characteristics of the study participants:	n=12 (23.5%).<br>• Median time of follow-up: 8.0 months (Q1-Q3: 4.0-10.0).<br>• Of the 51 patients included in the study, 15 (29.4%), 11 (21.6%), 13 (25.5%) and 12 (23.5%) had available data up to 3, 6, 9 and 12 months, respectively.<br>• The primary endpoint was total body weight loss percentage (TBWL%) at the last follow-up (last time point at which data were documented or	mg/dL (from 48.0% at baseline to 29.0% at the last follow-up; p<0.0001). There was no significant change in median TBR <70 mg/dL among CGM users: 1.0% at baseline vs. 1.5% at the last follow-up; p=0.60).<br>• At the last follow-up, there was a significant reduction in TDD of insulin (by 25 units; corresponding to 31.6% reduction): median TDD of insulin at baseline: 69 U/day vs. median TDD of insulin at last follow-up: 44 U/day; p<0.0001]; there was a precipitous (and statistically significant) decrease in TDD of insulin (to almost half of the daily insulin requirements) during the first 6 months of follow-up. Similar trends of reduction were also observed (particularly during the first 6 months of follow-up) for total daily basal insulin and total daily bolus insulin doses.<br>• At the last follow-up, tirzepatide therapy was associated with a significant decrease in median values of total cholesterol (160.0 mg/dL at baseline vs. 149.0 mg/dL at the last follow-up; p=0.0009), LDL cholesterol (84.5 mg/dL at baseline vs. 77.0

	median age, 50.0 years (IQR: 39.0-58.0); female participants, n=30 (58.8%); race/ethnicity, White (n=49; 96.1%); median body weight, 111.0 kg (IQR: 93.6-129.0); median BMI, 36.1 kg/m^2 (IQR: 32.0-42.6); prevalence of overweight, 11.8% (n=6); prevalence of class 1 obesity, 25.5% (n=13); prevalence of class 2 obesity, 21.6% (n=11); prevalence of class 3 obesity, 41.2% (n=21); prevalence of hyperlipidemia, n=41 (80.4%); prevalence of hypertension, n=38 (74.5%); prevalence of anxiety, n=28 (54.9%); prevalence of obstructive sleep apnea, n=23 (45.1%); prevalence of depression, n=21 (41.2%); prevalence of gastroesophageal reflux disease, n=17 (33.3%); prevalence of metabolic dysfunction-	tirzepatide therapy was discontinued).<br><br>• Secondary endpoints were the following: TBWL% at 3, 6, 9 and 12 months of tirzepatide therapy; change in HbA1c value; change in TDD of insulin; change in basal and bolus total insulin doses; change in CGM metrics (TIR 70-180 mg/dL, TAR >180 mg/dL, TBR <70 mg/dL) from baseline to last follow-up; and incidence of	mg/dL at the last follow-up; p=0.0057), triglycerides (109.5 mg/dL at baseline vs. 86.0 mg/dL at the last follow-up; p=0.01), alanine aminotransferase (28.0 U/L at baseline vs. 20.0 U/L at the last follow-up; p=0.02), and diastolic blood pressure (80.5 mmHg at baseline vs. 77.0 mmHg at the last follow-up; p=0.046).<br><br>• There was a reduction in median values of systolic blood pressure (130.0 mmHg at baseline vs. 126.0 mmHg at the last follow-up), although it did not reach statistical significance (p=0.10).<br><br><u>Side effects/adverse reactions</u>:<br><br>• Almost one-third of patients (n=15; 29.4%) reported at least one side effect of tirzepatide, with the most common being nausea (n=7; 13.7%).<br>• Only 5.9% of patients (n=3) reported moderate-to-severe gastrointestinal side effects that caused medication discontinuation, whereas 7.8% of patients (n=4) reported moderate gastrointestinal symptoms requiring tirzepatide dose de-escalation.<br>• 7.8% of patients (n=4) reported hypoglycemia, although none

	associated steatotic liver disease, n=6 (11.8%); median duration of diabetes, 24.0 years (IQR: 14.5-34.3); median HbA1c, 7.6% (IQR: 6.8-8.3); median TIR 70-180 mg/dL, 51.0% (IQR: 37.8-73.5); median TAR >180 mg/dL, 48.0% (IQR: 25.5-61.3); median TBR <70 mg/dL, 1.0% (IQR: 1.0-2.0); use of a CGM device, n=46 (90.2%); insulin pump use, 78.4% (n=40; use of closed-loop systems, n=28); median TDD of insulin, 68.6 U/day (IQR: 49.1-102.0); median daily basal insulin dose, 39.0 U/day (IQR: 27.7-57.3); median daily bolus insulin dose, 28.0 U/day (IQR: 18.0-51.7).	adverse events.	required assistance, hospitalization or medication discontinuation [hypoglycemia data were based on self-reported hypoglycemia (that was documented in clinical notes or in patient-provider communications) and on capillary blood or interstitial glucose levels from CGM reports (when available)].<br>• There were no reported cases of DKA.<br>• There were no reported cases of severe hypoglycemia or DKA even when Authors considered patients who were excluded from the statistical analysis for not having at least 3 months of data.
Case report (Ahmed et al. 2024 - Ref. 132)	• 38-year-old man with a 16-year history of T1D presenting with poor glucose control while he	• The patient was prescribed tirzepatide at a starting	• During the first 30 days after the initiation of tirzepatide therapy, the patient's daily glucose levels remained consistently within the range 100-150 mg/dL, with only

	was on MDI insulin therapy (with prandial insulin lispro-aabc plus basal insulin degludec) and was wearing a CGM sensor.<br><br>• The patient's glucose levels ranged between 75 mg/dL and 200 mg/dL, with intermittent hyperglycemic episodes (>200 mg/dL).<br><br>• Patient's comorbidities: hypertension; steatotic liver disease; combined hyperlipidemia; stage 2 chronic kidney disease; renal hyperparathyroidism; diabetic nephropathy; macroalbuminuria.<br><br>• The patient's body weight was 126.5 kg (BMI was not specified in the text), while his systolic blood pressure and diastolic	weekly dose of 2.5 mg.<br><br>• The weekly tirzepatide dose was increased to 5 mg at 30 days from tirzepatide therapy initiation.<br><br>• The weekly tirzepatide dose was then increased to 7.5 mg after about 4 weeks from the initiation of 5-mg weekly tirzepatide therapy.<br><br>• During this period, the patient also experienced a reduction in TDD of insulin (from about 45	a few hypoglycemic episodes.<br><br>• The weekly tirzepatide dose was increased to 5 mg at 30 days from tirzepatide therapy initiation.<br><br>• Afterwards, the daily glucose levels remained consistently within the range 80-140 mg/dL, without significant hyperglycemic episodes and rare hypoglycemic episodes.<br><br>• The weekly tirzepatide dose was then increased to 7.5 mg after about 4 weeks from the initiation of 5-mg weekly tirzepatide therapy.<br><br>• After about 2 months from the initiation of 7.5-mg weekly tirzepatide therapy, the patient interrupted the use of tirzepatide for about 30 days due to a family emergency, thus experiencing a weight gain of 1.5 kg.<br><br>• Subsequently, the patient resumed tirzepatide therapy at a low maintenance weekly dose of 5 mg.<br><br>• At the last follow-up visit, which was performed at 6 months from the initiation of tirzepatide therapy, the patient showed a

	blood pressure values were reported to be around 165 mmHg and 100 mmHg, respectively.<br>• The most recent urine albumin-creatinine ratio (uACR) value at the time of presentation was 700 mg/g.	units/day pre-tirzepatide therapy to about 46 units/day).<br>• After about 2 months from the initiation of 7.5-mg weekly tirzepatide therapy, the patient missed the tirzepatide injections for about 30 days due to a family emergency.<br>• Subsequently, the patient resumed tirzepatide therapy at a low maintenance weekly dose of 5 mg.<br>• Duration of tirzepatide therapy:	body weight of 104.6 kg (-21.9 kg vs. pre-tirzepatide therapy), along with decreased values of HbA1c (5.8%) [HbA1c values prior to tirzepatide therapy were not specified in the text], uACR (117 mg/g) and systolic and diastolic blood pressure (about 130/80 mmHg vs. 165/100 mmHg pre-tirzepatide therapy).

		6 months.	
Case series (Seetharaman and Cengiz; 2024 - Ref. 139)	• Case series including adolescents and young adults (AYA) with T1D who received GLP-1 RA therapy (as an adjunct therapy to insulin).<br><br>• Patients were selected based on clinical needs, particularly concurrent obesity or suboptimal glucose control.<br><br>• The GLP-1 RAs used in this case series included semaglutide and tirzepatide.<br><br>• This case series included 8 patients. 7 out of the 8 patients had obesity.<br><br>• The remaining patient was an 18-year-old female with T1D for 5 years (managed with Omnipod	• Case series of 8 AYA with T1D who received once-weekly subcutaneous semaglutide or tirzepatide at different doses and for different periods of time.	• Most patients in this case series showed notable improvements in HbA1c (up to -2.2%), TIR (up to +27%), average glucose levels (up to -50 mg/dL), reductions in TDD of insulin (up to -0.67 U/kg/day) and mild-to-moderate weight loss (up to 20.5 kg; except for one patient) after treatment with semaglutide or tirzepatide (used for up to 16 months).<br><br><u>Side effects/adverse reactions</u>:<br><br>• A significant proportion of patients experienced common gastrointestinal side effects of GLP-1 RAs, such as nausea, vomiting and diarrhea, even when these medications were used at low doses.<br>• Gastrointestinal side effects generally improved after a few weeks, with some patients managing them with the use of histamine H2-receptor antagonists and/or antiemetic drugs. Gradual GLP-1 RA dose titration helped minimize these common gastrointestinal side effects.

	insulin pump and Dexcom CGM sensor), who had a BMI of 22.9 kg/m^2 (body weight: 60.9 kg; 134 lbs) and was prescribed once-weekly subcutaneous semaglutide (at a starting dose of 0.25 mg/week) as an adjunct therapy for postprandial hyperglycemia and appetite regulation.		• One patient (a 14-year-old female with a 3-year history of T1D, managed with an Omnipod insulin pump and with a Dexcom CGM sensor) experienced an increase in hypoglycemic episodes, thus requiring a decrease in her daily insulin dose.<br>• There were no reported episodes of DKA.<br>• No patients exhibited depression, suicidal thoughts or mood issues.
Retrospective chart review (Snell-Bergeon et al. 2025 - Ref. 136)	• Retrospective chart review to assess the safety and efficacy of off-label semaglutide and tirzepatide use in adult patients with T1D over a period of 1 year.<br>• Inclusion criteria: age >18 years; prescribed semaglutide or tirzepatide for at least 3 months or frequency matched control not using weight-loss drugs; and use of intensive insulin	• Patients were started on an initial weekly dose of 0.25 mg for semaglutide and of 2.5 mg for tirzepatide, as per clinical guidelines.<br>• The median weekly dose of semaglutide prescribed was 0.5	• At 12 months, BMI decreased by a least square mean±standard error (SE) of 3.0±0.5 kg/m^2 and 7.5±0.5 kg/m^2 in the semaglutide and tirzepatide groups, respectively.<br>• There was no significant change in BMI in the control group at any time point.<br>• At 12 months, body weight decreased by a least square mean±SE of 19.2±3.0 lbs (9.1%) and 49.4±3.0 lbs (21.4%) in the semaglutide and tirzepatide groups, respectively.<br>• BMI, weight in pounds and weight percentage decreased significantly more in the tirzepatide group

	therapy through MDI, insulin pumps or AID systems.<br>• Exclusion criteria: diagnosis of T2D; pregnancy during the study period; and use of any other weight-loss drug.<br>• 208 patients (86 using semaglutide, 122 using tirzepatide) were excluded from the study due to missing data and due to the fact that they did not meet inclusion or exclusion criteria.<br>• Many visits were conducted via telehealth during the COVID-19 pandemic, which led to some missing HbA1c and body weight values.<br>• 12 patients who were prescribed tirzepatide had used semaglutide prior to	mg, while the median weekly dose of tirzepatide prescribed was 7.5 mg weekly.<br>• Two semaglutide users remained at the initial weekly dose of 0.25 mg, while the number of patients reaching the weekly semaglutide doses of 0.5 mg, 1 mg, and 2 mg was 38 (76%), 9 (18%), and 1 (2%), respectively.<br>• Two tirzepatide users remained on the initial weekly dose of 2.5 mg	than in the control group at all time points from 3 to 12 months, and significantly more in the semaglutide group than in the control group at 6, 9, and 12 months.<br>• The reduction in BMI, weight in pounds and weight percentage was significantly greater in the tirzepatide group than in the semaglutide group at all time points.<br>• At 12 months, 93% of tirzepatide users, 77% of semaglutide users and 14% of controls had lost 5% of their baseline body weight ($p < 0.0001$), while 87% of tirzepatide users, 47% of semaglutide users and none of the controls had lost 10% or more of their baseline body weight ($p<0.0001$).<br>• At 12 months, there was a significant reduction in the least square mean±SE HbA1c of 0.54±0.14% in the semaglutide group, and a reduction in the least square mean±SE HbA1c of 0.68±0.16% in the tirzepatide group, with no change observed in HbA1c among controls (-0.04±0.13%).

	starting tirzepatide and were already included in the semaglutide case cohort; these patients were removed from the tirzepatide cohort to avoid duplication of these participants in the analysis and in line with the inclusion criteria of "no prior weight-loss drug use".<br>• The retrospective chart review included 100 patients who were prescribed semaglutide (n=50) or tirzepatide (n=50) and 50 controls frequency matched for age, sex, BMI, HbA1c and diabetes duration, and who did not receive any weight-loss drugs during the study period.<br>• Data were collected before the initiation of weight-loss drugs	weekly, while the number of patients reaching the weekly tirzepatide doses of 5.0 mg, 7.5 mg, 10 mg, 12.5 mg, and 15 mg was 16 (32%), 8 (16%), 14 (28%), 5 (10%), and 5 (10%), respectively.<br>• After starting the medication, three semaglutide users and one tirzepatide user discontinued the drug due to lack of response.<br>• The majority of patients in all groups (82% of controls, 84% of	• The reduction in HbA1c was significantly greater in the semaglutide group than in the control group (p=0.006) and in the tirzepatide group than in the control group ($p < 0.0001$) overall.<br>• Although there was no difference in the amount of reduction in HbA1c between semaglutide group and tirzepatide group overall (p=0.175), there was a greater reduction in the tirzepatide group than in the semaglutide group at 6 months.<br>• In the tirzepatide group, TDD of insulin as well as basal and bolus insulin doses (expressed either in U/day or U/kg/day) decreased significantly from baseline and to a greater extent than in the semaglutide and control groups at all time points (least square mean±SE TDD at 12 months: tirzepatide group, -26.4±3.8 U/day [-0.13±0.03 U/kg/day]; semaglutide group, -4.1±3.9 U/day [0.02±0.04 U/kg/day]; control group, 3.9±3.9 U/day [0.02±0.04 U/kg/day]; $p<0.05$).<br>• With regard to the comparison between semaglutide and

	(baseline) and for up to 1 year for each patient.<br><br>• Baseline characteristics of the study participants: least square mean±standard error (SE) age, 41±2 years (control group) vs. 42±2 years (semaglutide group) vs. 39±2 years (tirzepatide group); gender (number of male participants), n=28 (14%) [control group] vs. n=30 (15%) [semaglutide group] vs. n=28 (14%) [tirzepatide group]; least square mean±SE diabetes duration: 27±2 years [control group] vs. 27±2 years [semaglutide group] vs. 24±2 years [tirzepatide group]; non-Hispanic White (number of participants): n=90 (45%) [control group] vs. n=90 (45%) [semaglutide	semaglutide users, and 78% of tirzepatide users) had at least 6 months of available follow-up data.<br><br>• More than half of controls (58%) and semaglutide users (64%) had at least 9 months of available follow-up data, while just under half of tirzepatide users (48%) had at least 9 months of available follow-up data.	control groups in terms of daily insulin doses, only bolus insulin dose at 6 months significantly differed between semaglutide users and controls (least square mean±SE bolus insulin dose at 6 months: semaglutide group, -4.2±2.7 U/day; control group, 3.6±2.3 U/day; $p<0.05$).<br><br>• Changes in BMI, body weight and HbA1c did not differ significantly between groups based on insulin delivery method (MDI insulin therapy, insulin pump/AID).<br><br>• Weight loss remained significantly greater in tirzepatide-treated patients than in semaglutide-treated patients, regardless of the insulin delivery method.

	group] vs. n=86 (43%) [tirzepatide group]; least square mean±SE BMI: 34.4±0.8 kg/m^2 (control group) vs. 33.4±0.8 kg/m^2 (semaglutide group) vs. 35.0±0.8 kg/m^2 (tirzepatide group); least square mean±SE body weight: 98.9±2.6 kg (control group) vs. 96.7±2.6 kg (semaglutide group) vs. 103.7±2.6 kg (tirzepatide group); least square mean±SE HbA1c: 7.3±0.2% (control group) vs. 7.6±0.2% (semaglutide group) vs. 7.0±0.2% (tirzepatide group*) [*p<0.05 compared with semaglutide group]; least square mean±SE TDD of insulin: 61.6±4.6 U/day (control group) vs. 70.3±5.1 U/day (semaglutide group) vs. 76.5±4.8		

	U/day (tirzepatide group*) [*p<0.05 compared with the control group]; insulin pump users (number of participants): n=80 (40%) [control group] vs. n=64 (32%) [semaglutide group] vs. n=86 (43%) [tirzepatide group]; commercial health insurance (number of participants): n=88 (29%) [control group] vs. n=92 (46%) [semaglutide group] vs. n=94 (47%) [tirzepatide group].<br><br>• Baseline matching characteristics (age, sex, BMI, HbA1c and diabetes duration) were similar between the cases and controls, with no differences observed in matching variables between the cases and controls.		

	• Almost all study participants had commercial health insurance, and there were no group differences in terms of proportion of patients with commercial health insurance.<br><br>• At baseline, TDD of insulin and basal daily insulin dose were higher in the tirzepatide group than in the control group, while HbA1c was lower in the tirzepatide group than in the semaglutide group.<br><br>• Almost all patients who were prescribed semaglutide (n=36; 72%) and tirzepatide (n=43; 86%) were affected by obesity, while 11 (22%) semaglutide-treated patients and 7 (14%) tirzepatide-treated patients were	

	affected by overweight.<br><br>• All insulin pump users were using AID systems.		
Post-hoc analysis of SURPASS-2, SURPASS-3, SURPASS-4 and SURPASS-5 clinical trials (Peters et al. 2024 - Ref. 133)	• Post-hoc analysis aiming to assess changes in HbA1c and body weight associated with tirzepatide therapy in GADA (glutamic acid decarboxylase autoantibodies)-positive vs. GADA-negative participants with a clinical diagnosis of T2D.<br><br>• The post-hoc analysis based on pooled data from the aforementioned clinical trials was performed using mixed-model repeated measures from the efficacy analysis set, adjusting for study and baseline covariates including sex, age, BMI, HbA1c and GADA status.	• Study participants were randomly assigned to receive once-weekly tirzepatide (5 mg, 10 mg or 15 mg), once-weekly semaglutide (1 mg) (SURPASS-2), insulin degludec (SURPASS-3), insulin glargine (SURPASS-4), or a volume-matched placebo in a single-dose pen (SURPASS-5).<br><br>• The primary outcome	• Change from baseline in HbA1c and body weight over time was expressed as least squares mean (LSM)±standard error (SE).<br><br>• Both GADA-positive and GADA-negative patients achieved significant reductions from baseline in HbA1c values over time (p<0.001 vs. baseline for both groups at all time points).<br><br>• At week 40/42, both GADA-positive and GADA-negative patients achieved significant HbA1c reductions, with slightly greater reductions in HbA1c observed in GADA-negative participants than in GADA-positive participants (-2.32% vs. -2.11%; estimated between GADA subgroup differences: 95% CI, 0.21% [0.03%-0.39%]; p=0.024).<br><br>• Significant reductions from baseline in HbA1c were observed in tirzepatide-treated GADA-positive patients, regardless of having low or high

	• Key eligibility criteria included the following: adults with T2D (HbA1c ≥7.0% or ≥7.5% to ≤10.5% at screening), a BMI of ≥23 or ≥25 kg/m^2, and stable weight (±5%), stable diabetes treatment during the previous 3 months with metformin at a daily dose ≥1500 mg prior to screening (SURPASS-2 and ± sodium-glucose cotransporter-2 inhibitors in SURPASS-3), 1 to 3 oral glucose-lowering medications, which could include only metformin, sodium-glucose co-transporter-2 inhibitors (SGLT-2is), and sulfonylurea (SURPASS-4), or stable doses of once-daily insulin glargine (at a dose greater than 0.25 U/kg/day or greater than 20 U/day)	of this post-hoc analysis was change from baseline in HbA1c in tirzepatide-treated patients from SURPASS-2 through SURPASS-5 diagnosed with apparent T2D and who were positive for GADA vs. negative for GADA.<br><br>• Secondary outcomes were change from baseline in body weight in GADA-positive vs. GADA-negative participants.<br><br>• Among those	GADA levels over time: at week 40/ 42, HbA1c reductions were -2.17% in GADA-positive participants with low GADA levels and -2.01% in GADA-positive participants with high GADA levels.<br><br>• Tirzepatide-treated GADA-positive patients from SURPASS-2 and SURPASS-4 achieved improvements in glucose control irrespective of baseline fasting C-peptide levels.<br><br>• Both GADA-positive and GADA-negative patients showed significant reductions from baseline in body weight over time ($p<0.001$; for both groups at all time points).<br><br>• At week 40/42, there was no significant difference in body weight reductions between GADA-negative patients and GADA-positive patients (-9.6 kg [-10.2%] vs. -9.2 kg [-10.4%]; estimated treatment differences: 95% CI, 0.38 kg [-0.99 to 1.75 kg]; p=0.588).<br><br>• The significant reductions from baseline in body weight were observed in tirzepatide-treated GADA-positive patients regardless of having low or

	with or without metformin (at a daily dose ≥1500 mg) for 3 months prior (SURPASS-5). LADA as a subgroup was not investigated in these trials. SURPASS-1 was excluded from this analysis since GADA were not measured in this trial.<br>• C-peptide, HOMA2-IR, and HOMA2-B (both computed with C-peptide) were evaluated.<br>• Insulin use throughout the duration of each trial and hypoglycemic events by GADA status were also assessed.<br>• Baseline characteristics of the study participants (3791 participants): 120 GADA-positive participants and 3671 GADA-negative participants). Mean (±SD) age,	with detectable GADA levels, treatment outcomes in tirzepatide-treated GADA-high level vs. GADA-low level participants were measured.	high GADA levels over time: at week 40/42, body weight reductions with tirzepatide were -9.1 kg (-9.8%) in GADA-positive patients with low GADA levels and -9.5 kg (-11.0%) in GADA-positive patients with high GADA levels.<br>• A pooled analysis of SURPASS-2 and SURPASS-4 clinical trials showed that baseline HOMA2-B (homeostasis model assessment of beta-cell function; calculated with fasting C-peptide) increased significantly from baseline over time in both GADA-positive and GADA-negative patients during tirzepatide therapy (by 62.4% and 97.0%, respectively; p<0.001; for both subgroups), with improvement in HOMA2-B being significantly greater among GADA-negative patients than in GADA-positive patients at week 40/52 (estimated difference vs. GADA-positive patients: 95% CI, 21.3% [7.6%-36.8%]; p=0.002).<br>• HOMA2-IR (homeostasis model assessment of insulin resistance; calculated with fasting C-peptide) decreased significantly from baseline

	59.5±10.92 years (GADA-positive patients) vs. 58.9±10.19 years (GADA-negative patients); sex [female; n (%)], 55 (45.8%) [GADA-positive patients] vs. 1706 (46.5%) [GADA-negative patients]; mean (±SD) duration of diabetes, 10.1±7.03 years (GADA-positive patients) vs. 9.8±7.02 years (GADA-negative patients); mean (±SD) HbA1c, 8.5±0.94% (GADA-positive patients) vs. 8.3±0.96% (GADA-negative patients); mean (±SD) fasting serum glucose, 172.5±50.01 mg/dL (GADA-positive patients) vs. 171.7±50.95 mg/dL (GADA-negative patients); mean (±SD) body weight, 88.6±18.35 kg (GADA-positive patients) vs. 93.3±20.64		in both GADA-positive and GADA-negative patients during tirzepatide therapy by 27.2% and 16.9%, respectively ($p <0.001$, for both subgroups), with improvement in HOMA2-IR being significantly greater among GADA-positive patients than in GADA-negative patients at week 40/52 (estimated difference vs. GADA-negative patients: 95% CI, 14.3% [1.9%-28.2%]; $p=0.023$).<br><br>Side effects/adverse reactions:<br><br>• Hypoglycemia was reported in 151 out of 3678 GADA-negative patients as compared to 12 out of 120 GADA-positive patients (6 patients with high GADA levels and 6 patients with low GADA levels); yet, 7 of these 12 GADA-positive patients were on insulin therapy, while none of these patients were concomitantly using sulfonylureas.<br>• There was no reported case of severe hypoglycemia in GADA-positive patients.

	kg (GADA-negative patients); mean (±SD) BMI, 32.2±6.08 kg/m² (GADA-positive patients) vs. 33.6±6.27 kg/m² (GADA-negative patients); use of oral glucose-lowering medications [n, (%)], 117 (97.5%) [GADA-positive patients] vs. 3614 (98.4%) [GADA-negative patients]; mean (±SD) total cholesterol, 169.0±37.82 mg/dL (GADA-positive patients) vs. 170.6±42.62 mg/dL (GADA-negative patients); mean (±SD) HDL cholesterol, 45.2±11.62 mg/dL (GADA-positive patients) vs. 43.8±11.33 mg/dL (GADA-negative patients); mean (±SD) LDL cholesterol, 90.2±32.81 mg/dL (GADA-positive patients)		

vs. 90.5±34.76 mg/dL (GADA-negative patients); mean (±SD) triglycerides, 169.2±101.28 mg/dL (GADA-positive patients) vs. 188.1±131.38 mg/dL (GADA-negative patients); mean (±SD) SBP (systolic blood pressure), 130.7±15.90 mmHg (GADA-positive patients) vs. 132.5±14.38 mmHg (GADA-negative patients); mean (±SD) DBP (diastolic blood pressure), 77.7±8.63 mmHg (GADA-positive patients) vs. 79.1±9.27 mmHg (GADA-negative patients); mean (±SD) pulse rate, 74.8±9.60 bpm (GADA-positive patients) vs. 74.3±10.25 bpm (GADA-negative patients).

Case report (Suzuki; 2024 - Ref. 134)	• 67-year-old man affected by mitochondrial diabetes mellitus (MDM), a rare form of diabetes mellitus caused by mutations in mitochondrial DNA and often associated with other comorbidities such as GAD antibody positivity.<br>• The patient was affected by MDM with a mutation at position 3271.<br>• The patient also had a past history of transient GAD antibody positivity and autoimmune pancreatitis, which went into spontaneous remission.<br>• The patient, who had a history of treatment with oral glucose-lowering medications for several years, experienced poor glycemic control after pancreatitis.	• 5-month tirzepatide therapy (the exact tirzepatide dose and titration scheme were not specified).	• After 5 months of tirzepatide therapy, HbA1c decreased from 7.0% to 6.4% and serum C-peptide value decreased from 1.04 ng/mL to 0.89 ng/mL.<br>• The decrease in serum C-peptide values was interpreted as a likely consequence of the reduction in insulin resistance.<br>• Other notable findings observed after tirzepatide therapy were a body weight loss of 3.3 kg and discontinuation of glimepiride.<br>• GAD antibody remained negative and autoimmune pancreatitis did not relapse over the course of tirzepatide therapy.

- Baseline body weight and BMI values were not specified in the manuscript.

Abbreviations: 95% CI, 95% Confidence interval; AGP, Ambulatory glucose profile; AID, Automated insulin delivery; AYA, Adolescents and young adults; BMI, Body mass index; CGM, Continuous glucose monitoring; COVID-19, Coronavirus disease 2019; CV, Coefficient of variation; DBP, Diastolic blood pressure; DKA, Diabetic ketoacidosis; FDA, U.S. Food and Drug Administration; GAD, Glutamic acid decarboxylase; GADA, Glutamic acid decarboxylase autoantibodies; GIP, Glucose-dependent insulinotropic polypeptide; GLP-1, Glucagon-like peptide-1; GMI, Glucose Management Indicator; HbA1c, Glycated hemoglobin; hCG, Human chorionic gonadotropin; HDL, High-density lipoprotein; HOMA2-B, Homeostasis model assessment of beta-cell function; HOMA2-IR, Homeostasis model assessment of insulin resistance; IQR, Interquartile range; LDL, Low-density lipoprotein; LSM, Least squares mean; MDI, Multiple daily injection; MDM, Mitochondrial diabetes mellitus; ns, non-significant; p, p-value; Q1-Q3, Interquartile range; RA, Receptor agonist; RAs, Receptor agonists; SBP, Systolic blood pressure; SD, Standard deviation; SE, Standard error; SGLT-2is, Sodium-glucose cotransporter-2 inhibitors; T1D, Type 1 diabetes; T2D, Type 2 diabetes; TAR, Time above range; TBR, Time below range; TBWL%, Total body weight loss percentage; TDD, Total daily dose; TIR, Time in range; TITR, Time in tight range; uACR, Urine albumin-creatinine ratio.
